# New material of *Epiaceratherium* and a new species of *Mesaceratherium* clear up the phylogeny of early Rhinocerotidae (Perissodactyla)

**DOI:** 10.1098/rsos.200633

**Published:** 2020-07-15

**Authors:** Jérémy Tissier, Pierre-Olivier Antoine, Damien Becker

**Affiliations:** 1 JURASSICA Museum, Porrentruy, Jura Canton, Switzerland; 2 Department of Geosciences, University of Fribourg, Fribourg, Fribourg Canton, Switzerland; 3 Institut des Sciences de l'Évolution, Université de Montpellier, Montpellier, France

**Keywords:** *Epiaceratherium*, *Mesaceratherium*, new species, Rhinocerotidae, Oligocene, phylogeny

## Abstract

Reduction of the anterior dentition (i.e. incisors and canines) is a major adaptative trait of the Rhinocerotidae among Perissodactyla. However, the corresponding evolutionary sequence was lacking a robust phylogenetic frame to support it thus far. Here, we describe a new Oligocene species of Rhinocerotinae, *Mesaceratherium* sp. nov. from the Swiss locality of Bumbach (MP25 reference level). In addition, we identify the only known complete mandible of *Epiaceratherium magnum*, an early-branching rhinocerotid, as well as one of the earliest European rhinoceroses. We also compute a parsimony analysis based on morpho-anatomical characters to assess their phylogenetic position and elucidate the early evolution of the Rhinocerotidae. Our results allow to propose a new scenario for the reduction of the anterior dentition in which upper and lower dentitions would have undergone distinct evolutionary trajectories.

## 1. Introduction

Rhinocerotoidea have a relatively recent history in Western Europe, compared to their long-lasting history in Asia and North America, where they developed since the Early and early Middle Eocene, respectively [1,2]. Indeed, they first appear in Western Europe after the Eocene–Oligocene transition, and the related Grande Coupure event [3–6]. Five genera of rhinocerotoids, belonging to three distinct families (Hyracodontidae, Amynodontidae and Rhinocerotidae), are known in Western Europe during the Early Oligocene interval: the eggysodontid *Eggysodon*, the amynodontid *Cadurcotherium* and the rhinocerotids *Epiaceratherium*, *Molassitherium* and *Ronzotherium*. Among these three families, only the Rhinocerotidae survived the Oligocene–Miocene transition and have living representatives, in Africa and Asia [7].

The first appearance of Rhinocerotidae in Europe is mostly explained by dispersals from Asia, documenting the Grande Coupure event [1,4,8–10]. Indeed, *Epiaceratherium* and *Ronzotherium* may have close Late Eocene relatives in Asia [1,11,12]. In Europe, *Epiaceratherium* is only known from a few Early Oligocene localities [5,10,12–15] contrary to *Ronzotherium* which is a conspicuous element of mammalian assemblages throughout the Oligocene epoch [16,17]. *Epiaceratherium bolcense* Abel, 1910 [18] is documented by numerous remains from the earliest Oligocene locality of Monteviale in Italy [13,15] but it has never been recognized anywhere else. *Epiaceratherium magnum* Uhlig, 1999 [14] is mostly represented by isolated dental and postcranial remains from several Early Oligocene localities of Germany, France, Switzerland and Czech Republic, i.e. across the Molasse Basin [5,10,14]. Later on, several remains originally referred to as *Epiaceratherium* aff. *magnum* by Uhlig [14] were assigned to *Molassitherium delemontense* Becker & Antoine, 2013 [19]. The type species of *Molassitherium* Becker & Antoine, 2013 [19] is *Molassitherium albigense* (Roman, 1912) [20], from the late Early and early Late Oligocene of France and the Iberian Peninsula [19,21].

The first occurrence of *Mesaceratherium* Heissig, 1969, another European rhinocerotid, is recorded during the Late Oligocene [16]. The type species *M. gaimersheimense* Heissig, 1969 [16] was first discovered in the south German locality of Gaimersheim, along with *Ronzotherium*, but has now been identified in France and Switzerland as well [22,23]. Two other species are reported from the earliest Miocene: *M. paulhiacense* (Richard, 1937) [24] from France and *M. welcommi* Antoine & Downing, 2010 [25] from Pakistan.

Here, we describe an unpublished mandible (NMB.O.B.928) from the ‘middle Oligocene’ Rheinbetts locality (Basel Canton, Switzerland) and newly restored mandibular and postcranial remains from the Bumbach locality (MP25 reference level; Bern Canton, Switzerland). We also compute a parsimony analysis based on morpho-anatomical features and including well-documented early Holarctic rhinocerotids as terminals, in order to assess their phylogenetic affinities and, more broadly, to enlighten the early evolutionary history of the Rhinocerotidae. This phylogenetic framework further allows for inferring the evolution of one of the major adaptative traits of this group, i.e. their anterior dentition (incisors and canines).

## 2. Material and methods

### 2.1. Specimens

The newly described mandible NMB.O.B.928 from Rheinbetts is housed in the Naturhistorisches Museum Basel, Switzerland (NMB), while the specimens from Bumbach attributed to *Molassitherium albigense* are housed in the Naturhistorisches Museum der Burgergemeinde Bern, Switzerland (NMBE). The abbreviations of other institutions are: BSPG, Bayerische Staatssammlung für Paläontologie und Geologie, Munich (Germany); DGMV, Department of Geology and Minerals of Vietnam; MJSN, JURASSICA Museum of Porrentruy (Switzerland); MHNT, Muséum d'histoire naturelle de Toulouse (France); FSL, Université Claude-Bernard-Lyon-I (France).

### 2.2. Surface scanning

Specimens have been scanned with a structured-light surface scanner (Artec Space Spider, Artec Group) and the three-dimensional models were reconstructed using the Artec Studio 13 Professional software. These three-dimensional models are available in Tissier *et al.* (2020) [26].

### 2.3. Terminology

The morpho-anatomical characters described here follow the terminology of Antoine [27]. The dental abbreviations are as follows: c/C, lower/upper canine; d/D, lower/upper decidual tooth; i/I, lower/upper incisor; m/M, lower/upper molar; p/P, lower/upper premolar. Dental measurements were taken according to Uhlig [14]. The postcranial abbreviations are as follows: Mc, metacarpal; Mt, metatarsal. Measurements are given in millimetres, and measurements in parentheses are estimated. Abbreviations used for measurements are as follows: ant, anterior; APD, anteroposterior diameter; dia, diaphysis; dist, distal; H, height; post, posterior; prox, proximal; TD, transverse diameter; W, width.

### 2.4. Characters matrix and phylogeny

The character matrix is available in electronic supplementary material, S1 and is based on Antoine [27] with six additional characters:

— 283: p3, lingual branch of the paralophid: 0, developed; 1, reduced
— 284: p3–4, anterolingual cingulid: 0, stopping at the anterior valley or absent; 1, joining metaconid
— 285: P2, metacone fold: 0, strong; 1, weak or absent
— 286: P3–4, metacone fold: 0, strong; 1, weak or absent
— 287: M1–2, parastyle: 0, long; 1, short
— 288: I1, shape = 0, spatulate; 1, conical and pointed; 2, chisel (ordered)
Parsimony analyses were computed with the software PAUP* v. 4.0a (build 167) [28]. All characters were set as ordered, except characters 72, 94, 102, 103, 140, 187 and 190, which do not form morphoclines, and all characters have equal weights.

We modified characters 2 and 3 from the original matrix of Antoine [27] as follows:

— 2: Maxilla: foramen infraorbitalis: 0, above P1–2; 1, above P3; 2, above P4; 3, above molars
— 3: Nasal notch: 0, above P1–2; 1, above P3; 2, above P4–M1

Several analyses were performed to assess the identification of the mandible NMB.O.B.928 from Rheinbetts and the material from Bumbach. We tested different sets of terminals, adding taxa by incrementation, or by merging some specimens into a single terminal. If, in the resulting trees, several specimens were grouped into a single clade with the holotype specimen, we merged them together. When merging the scores of these terminals, the differences in character states are considered as polymorphism in the new coding. During the first analysis, the taxonomic sample included two non-rhinocerotid perissodactyls as outgroups (*Tapirus terrestris* and *Hyrachyus eximius*) and the ingroup consisted of *Epiaceratherium naduongense*, *Epiaceratherium bolcense* and *Molassitherium delemontense*, as well as specimens attributed to *M. albigense*, specimens from Bumbach (scored in a single terminal) and the mandibular specimen from Rheinbetts (NMB.O.B.928). We first selected these taxa to test our *a priori* referrals to these respective genera (*Epiaceratherium* and *Molassitherium*). New terminals were then added consecutively, documenting other taxa to which they could be referred, especially within *Mesaceratherium* and *Pleuroceros*. Other taxa were added to test the monophyly of *Epiaceratherium* and *Molassitherium* and, more broadly, to better understand the early evolutionary steps of Rhinocerotidae, as well as to stabilize the topology of the tree and to test its robustness.

A branch-and-bound search algorithm was first used for the analyses encompassing 12 to 16 terminals, after which the heuristic algorithm was faster and almost as efficient. The addition sequence was set to ‘furthest’ during the branch-and-bound search. We used a random addition sequence of 1000 replicates and held 100 trees at each step during the heuristic search with a TBR swapping algorithm with no reconnection limit and swapping on all trees.

### 2.5. Nomenclatural act

The electronic edition of this article conforms to the requirements of the amended International Code of Zoological Nomenclature, and hence the new names contained herein are available under that Code from the electronic edition of this article. This published work and the nomenclatural acts it contains have been registered in ZooBank, the online registration system for the ICZN. The ZooBank LSIDs (Life Science Identifiers) can be resolved and the associated information viewed through any standard web browser by appending the LSID to the prefix http://zoobank.org/. The LSID for this publication is: urn:lsid:zoobank.org:pub:C4732CCF-996F-48D1-AC33-C1E733CBDFD9. The electronic edition of this work was published in a journal with an ISSN, and has been archived and is available from the following digital repositories: CLOCKSS, LOCKSS, Portico and PubMed Central.

**Systematic palaeontology**

**Perissodactyla** Owen, 1848 [29]

**Rhinocerotoidea** Owen, 1845 [30]

**Rhinocerotidae** Gray, 1821 [31]

***Epiaceratherium*** Abel, 1910 [18]

**Type species:** *Epiaceratherium bolcense* Abel, 1910 [18]

**Other species:** *Epiaceratherium magnum* Uhlig, 1999 [14]; *Epiaceratherium delemontense* comb. nov. (Becker & Antoine, 2013) [19]; *Epiaceratherium naduongense* Böhme *et al*., 2013 [12].

**Diagnosis:** Stem rhinocerotine lacking i3 and a lower canine, with a wide postfossette on P2–P4, a protoloph usually constricted on M1–M2, a straight posterior half of the ectoloph on M1–M2, and a posterior valley usually closed on p2.

**Stratigraphical distribution:** late Middle Eocene to Early Oligocene (South Asia) and Early to early Late Oligocene (Europe).

**Geographical distribution:** From East to West**,** Northern Vietnam, Pakistan, Czech Republic, Northern Italy, Germany, Switzerland and France.

*Epiaceratherium magnum* Uhlig, 1999 [14]

**Emended diagnosis:** large species of the genus with a horizontal mandibular symphysis with divergent i2, a metacone fold weak or absent on P2, a crista sometimes present on P3, a metaloph directed posterolingually on P3–4, a crochet usually present on upper molars, a crista usually absent on upper molars, a V-shaped ectolophid groove developed until the neck on lower cheek teeth, a single-rooted d1, an anteroposterior diameter/height ratio inferior to 0.65, and a nearly straight caudal border of the astragal trochlea. The skull is unknown.

**Type material:** Left M1 (BSPG-1972-XI-1930), M2 (BSPG-1972-XI-1930) and M3 (BSPG-1972-XI-1930).

**Type horizon and locality:** Fissure filling of Möhren 13 near Treuchtlingen (Franken Alb, Bavaria, Germany), possibly MP22 (Early Oligocene) based on faunal comparison.

**Figure 1.**
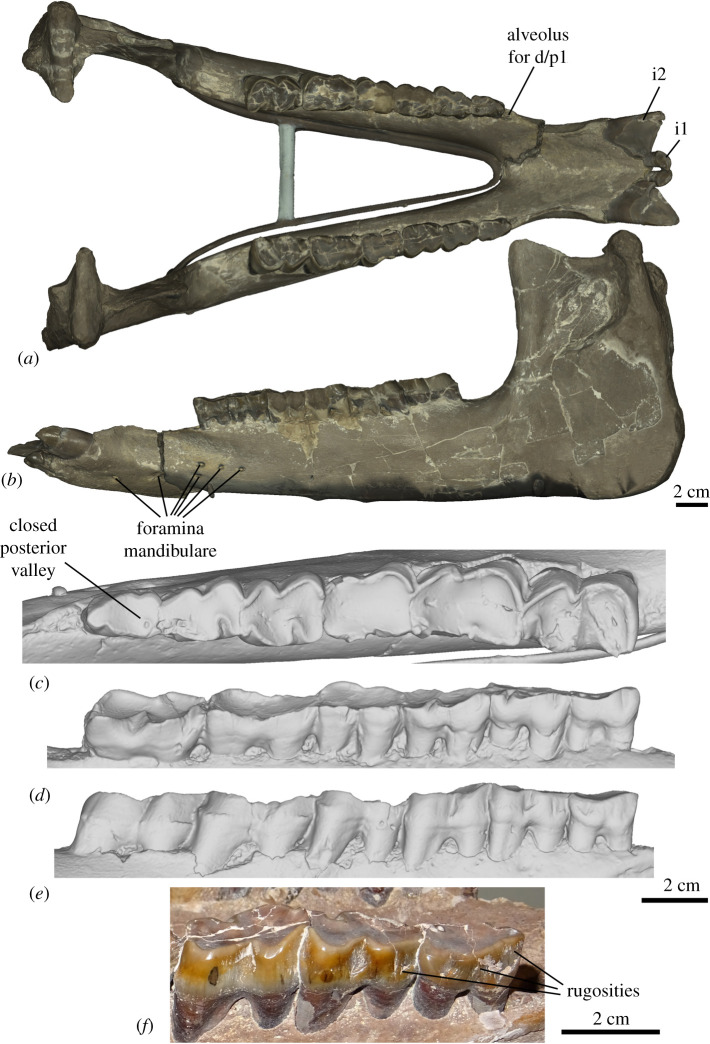
Three-dimensional models of the mandible of the Oligocene rhinocerotid *Epiaceratherium magnum* NMB.O.B.928, from Rheinbetts, Switzerland. (*a*) Occlusal view with texture; (*b*) lateral view with texture; (*c*) close-up occlusal view of the right cheek teeth series, with p2–m3; (*d*) same, in lingual view (note the distolingually twisted wear); (*e*) same, in labial view. (*f*) close-up view of the labial rugosities of the lower premolars. Scale bars equal 2 cm.

**Additional referred material:** NMB.O.B.928 (figure 1), a sub-complete mandible from the ‘Molasse Alsacienne’ formation of Rheinbetts (Basel Canton, Switzerland), dated from the ‘Middle Oligocene’.

**Stratigraphical distribution:** Early to early Late Oligocene.

**Geographical distribution:** Germany, France and Switzerland. *Epiaceratherium* cf. *magnum* was described in lower Oligocene deposits of Pakistan [1].

**Description.** The mandible NMB.O.B.928 (figure 1) is both well preserved (left ramus) and partly reconstructed (right ramus). The angle between the symphysis and the *corpus mandibulae* is particularly open, as the symphysis is almost horizontal. The symphysis is wide and without ventral keel. The dorsolateral borders of the symphysis, constricted, form sharp and acute dorsal ridges, between incisors and cheek teeth. The posterior edge of the symphysis is located at the level of p2. There are six *mental foramina* on each side: the two most anterior are large and ventral, whereas four others are more lateral and smaller. On the left side, the four smaller foramina are located below p1–2, whereas on the right two are located below p2–3 and other ones are anterior to p1. On the medial side of the *corpus mandibulae*, the groove for the mylohyoid nerves and vessels is well marked and extends from m1/2 to the ramus. The ventral edge of the corpus is completely straight in lateral view. The ramus is reconstructed on the right side, but partly preserved on the left. It is vertical, with a well-developed coronoid process. The large *foramen mandibulare* opens slightly below a hypothetical horizontal line formed by the teeth neck.

The lower dental formula is i1, i2, d/p1–m3. The premolar series is long compared to the molars (Lp3–4/Lm1–3 > 0.5). The cement is globally absent on cheek teeth. The first incisors are typically incisor shaped and well developed, with a clear neck and a thin lingual cingulid. They are partly worn by contact with upper teeth (and food) and almond shaped in occlusal outline. The very large second incisors are tusk-like and slightly divergent. They are also very worn, without a clear neck. The wear surface is triangular, with a much more worn medial side. There is no cingulid.

The p1 is absent on both sides, but there are small alveoli, anterior to p2, for a small single-rooted d/p1. There are very thin and smooth external rugosities on the ectolophid of p2–3. The ectolophid groove of p2–m3 is angular but vanishing above the neck. On p3, this groove is very oblique, and becomes almost horizontal above the neck. The trigonid of the cheek teeth is angular and forms an acute angle. The entoconid and metaconid are not constricted. The posterior valley of p2 is lingually closed, while on p3–4 it is very narrow and V-shaped, in lingual view. The lingual cingulid is completely absent on p2–m3. The labial cingulid is only partly present on m2–3, but very weak. The paralophid of p2 is developed, isolated and spur-like. The hypolophid of the lower molars is oblique. There is no lingual groove on the entoconid of m3 table 1.

**Table 1.** Measurements of the lower teeth of NMB.O.B.928 compared with other species of *Epiaceratherium*. Measurements are given in millimetres and are presented as length (or APD for incisors)/width (or TD for incisors)/height (for incisors only).

teeth	*E. magnum* NMB.O.B.928	*E. magnum* [14]	*E. bolcense* (NMB.I.O.23)	*E. naduongense* (DGMV SAU-08)
**i1**	13–12.7/9.3–8.6/10–9.5	11–14/9–12/x–x		11/8
**i2**	27.6–27.7/17–16.5/x–x	23–24/14–15.5/x–x		25/17
**p2**	20–21.5/13–14	18.5–22/12–14.5	18.5/10.7	22/13
**p3**	24–24.5/18.5–17.5	24–27.5/16–20	21.1–20.5/14–14.5	27/18
**p4**	26.5–26/x-20	25–29/18–22.5	22.5/16.1	27/21.5
**m1**	25.5–27.5/20.5–20.5	27–31.5/21–25	24.6/(18.3)	31/22.5
**m2**	34–35.5/24–24	31.5–36/22–26.5	28.2/19.3	35/24
**m3**	36–37/21–23	31–36.5/21–24.5		37/24

**Comparisons.** The mandible NMB.O.B.928 can be unambiguously attributed to a rhinocerotoid, based on the typical shape of the lower cheek teeth (ectolophodont cheek teeth with a *cristid obliqua* directed towards the protoconid). An attribution to the Amynodontidae, Eggysodontidae, Hyracodontidae or Paraceratheriidae can be excluded based on its anterior dentition, due to the tusk-shaped second lower incisor and the absence of third lower incisor and lower canine [32]. The mandible can be further referred to a small to middle-sized rhinocerotid, thus excluding large-sized European Oligocene rhinoceroses such as *Ronzotherium* or *Diaceratherium* [4,16,17,22].

Six genera of small to medium-sized Rhinocerotidae are known in Europe during the Oligocene–Early Miocene. *Pleuroceros*, *Plesiaceratherium* and *Protaceratherium* (*sensu* [19]) are all reported from the ?latest Oligocene to the Early Miocene [33]. *Pleuroceros* differs from NMB.O.B.928 in having a smooth and U-shaped external groove on lower cheek teeth (angular and V-shaped on NMB.O.B.928) and a continuous lingual cingulid on lower premolars (absent on NMB.O.B.928) [25]. *Plesiaceratherium* differs from the studied specimen in having premolars with a labial cingulid high above the base (absent on NMB.O.B.928), with shallow ectolophid groove (angular and V-shaped on NMB.O.B.928) [34]. *Protaceratherium* differs by the reduced first lower incisors (strong on NMB.O.B.928) and the presence of cingulid on the lower cheek teeth (mostly absent on NMB.O.B.928) [35]. Among Oligocene taxa, *Mesaceratherium* differs by its very upraised mandibular symphysis (horizontal on NMB.O.B.928) and the usual presence of lingual cingulid on the lower cheek teeth (absent on NMB.O.B.928) [16]. *Molassitherium albigense* (lower dentition unknown for *M. delemontense*) differs by the strong cingulid on the lower cheek teeth (mostly absent on NMB.O.B.928) [17]. The lower teeth of *Molassitherium delemontense* are very poorly known [19,36] and very similar, but they differ in having a slightly more oblique hypolophid on m1. *Molassitherium* cf. *delemontense* from Nuceto [36] have a more constricted paralophid on p2. To sum up, the mandible can be assigned to *Epiaceratherium*, as supported by our phylogenetic analyses. Both entities share the absence of i3 and c, the absence of lingual cingulid on the lower premolars, as well as external rugosities on the ectolophid of the premolars and the usually closed posterior valley on p2, which are diagnostic characters shared by all species of the genus [12]. Within this genus, *E. naduongense* and *E. bolcense* differ by the biradiculate d/p1 whereas it is uniradiculate on NMB.O.B.928, as in *E*. *magnum* [12,14]. *Epiaceratherium bolcense* further differs by the constriction of the metaconid on the cheek teeth [13], whereas *E. naduongense* differs by a slightly more convex ventral border of the mandible and a slightly more upraised symphysis [12]. Therefore, we refer to the mandible NMB.O.B.928 as *Epiaceratherium magnum*.

**Rhinocerotinae** Gray, 1821 [31]

***Mesaceratherium*** Heissig, 1969 [16]

**Type species:** *Mesaceratherium gaimersheimense* Heissig, 1969 [16]

**Other species:** *Mesaceratherium paulhiacense* (Richard, 1937) [24]; *Mesaceratherium welcommi* Antoine & Downing, 2010 [25]; *Mesaceratherium tschani* sp. nov.

**Diagnosis:** Medium-sized hornless rhinocerotine, with a strong paracone fold on M1–M2, a posterior McIII-facet on McII, no posterior MtII-facet on MtIII, with slender limbs, a transverse metaloph on P2, a lingual cingulid on lower premolars, and a curved magnum-facet on McII.

**Stratigraphical distribution:** MP25–MN3 (emended from [22]).

**Geographical distribution:** France, Switzerland, Germany, Pakistan [16,22–25,32,37].

*Mesaceratherium tschani* sp. nov.

urn:lsid:zoobank.org:act:C3E9079B-5DE8-4C50-9C78-ACE7DEF8C2DB

**Diagnosis:** Differs from all other assigned species by a ramus of the mandible inclined forward, a developed external groove on the lower cheek teeth, the absence of labial cingulid on the lower molars, radius and ulna with a marked contact on the diaphysis and the presence of a posterior expansion of the pyramidal facet on the unciform. Skull and upper dentition unknown.

Further differs from *M. gaimersheimense* in having a posterior border of the mandibular symphysis at the level of p2, a contact between the pyramidal and McV facets on the unciform, a keeled anterior side on the lunate and a pentagonal uniform facet on the McIV.

Differs from *M. welcommi* by a posterior border of the mandibular symphysis at the level of p2, lower cheek teeth without a constricted entoconid, an ectolophid groove deep to angular and not interrupted above the neck, an angular and acute trigonid, a rounded distal border of the lunate and a magnum-facet does not reach the anterior side of the lunate.

Differs from *M. paulhiacense* by the large pyramidal-facet on the unciform, the contact between the McV and pyramidal facets on the unciform, the circular posterior facet for the McIV on the McIII and the small-sized McV facet on the McIV.

**Etymology:** From the last name of the original person who discovered these specimens, Gottlieb Tschan, from Merligen (Bern Canton, Switzerland).

**Type locality and horizon:** Bumbach (Bern Canton, Switzerland), Swiss reference level for the MP25 biozone (early Late Oligocene).

**Type material:** Fragmented mandible NMBE5033614 with left p3 and m3 and right p1–3 and labial sides of p4–m2.

**Figure 2.**
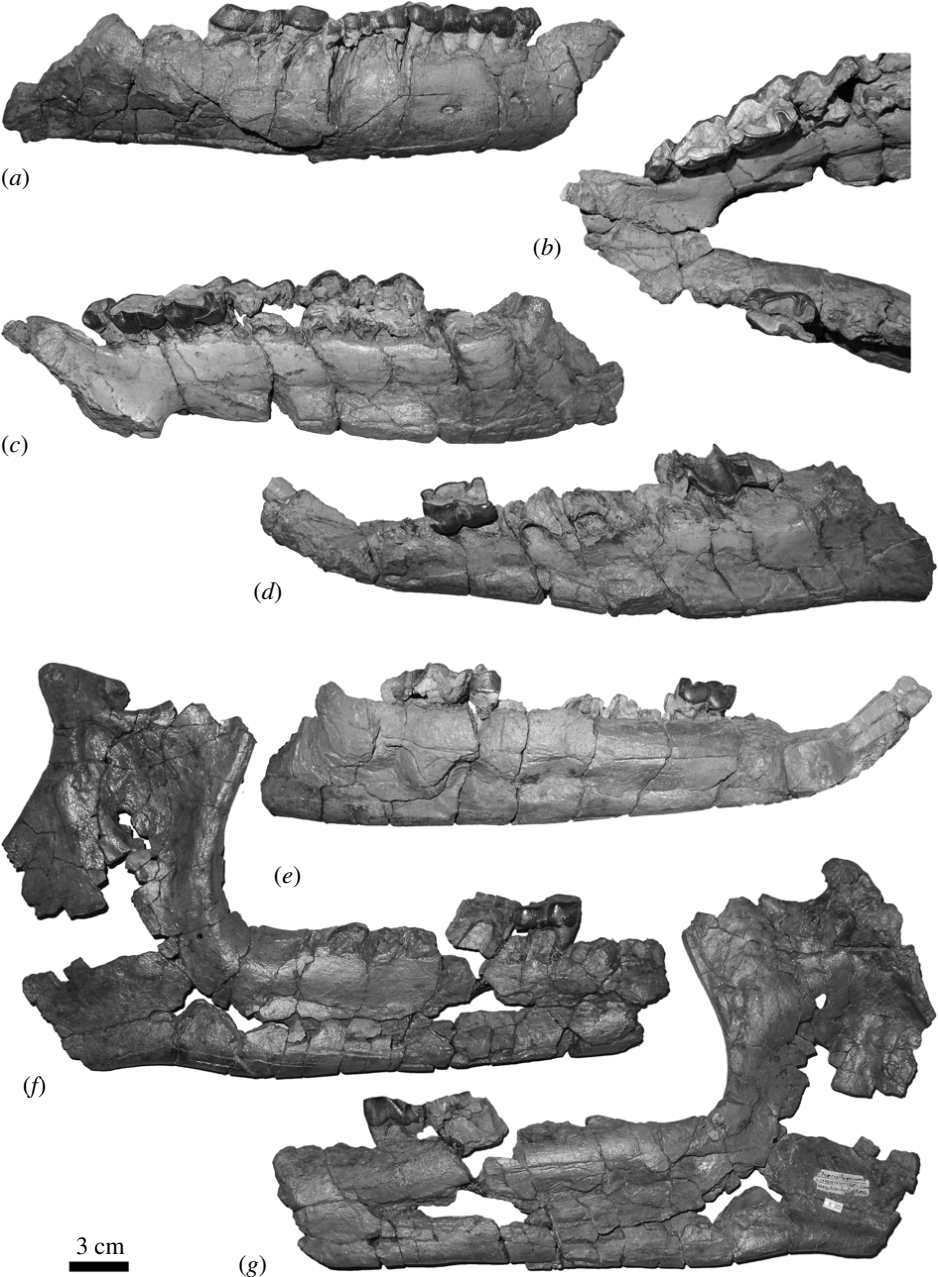
Mandibles of *Mesaceratherium tschani* sp. nov. from Bumbach (Switzerland; MP25). (*a*–*e*) mandible NMBE5033614; (*f*,*g*) mandible NMBE5033615. Scale bar equals 3 cm.

**Figure 3.**
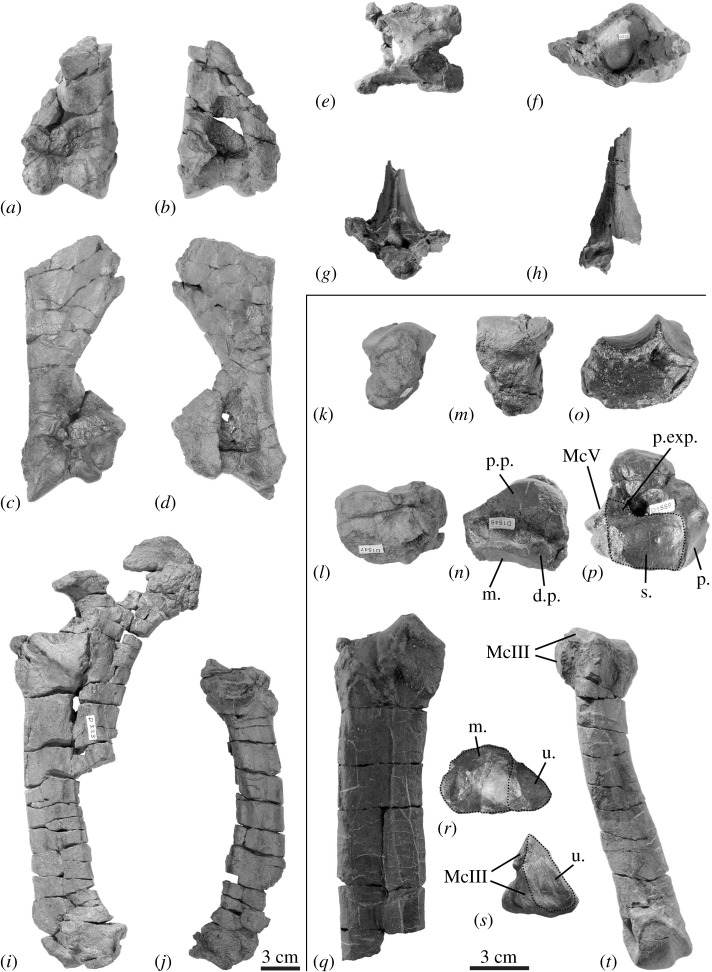
Postcranial remains of the Oligocene rhinocerotine *Mesaceratherium tschani* sp. nov. from Bumbach (Switzerland; MP25). (*a*,*b*) right humerus NMBE5033619; (*c*,*d*) left humerus NMBE5033620; (*e*,*f*) atlas NMBE5033616; (*g*) C: thoracic vertebra NMBE5033617; (*h*) thoracic vertebra NMBE5033618; (*i*) left radius-ulna NMBE5033621; (*J*) right radius NMBE5033622; (*k*,*l*) fragment of left scaphoid NMBE5033628; (*m*,*n*) left lunate NMBE5033627; (*o*,*p*) right unciform; NMBE5033623; (*q*,*r*) right McIII NMBE5033613; (*s,t*): left McIV NMBE5014497. d.p., distal pyramidal facet; m, magnum facet; McIII, McIII facet; McV, McV facet; p., pyramidal facet; p. exp., posterior expansion of the pyramidal facet; p.p., posterior pyramidal facet; s, semilunate facet; u, unciform facet.

**Other referred material:** Right mandible with p4–m1 (NMBE5033615); atlas (NMBE5033616); fragments of thoracic vertebra (NMBE5033617; NMBE5033618); distal fragments of right and left humeri (NMBE5033619, NMBE5033620); right radius (NMBE5033622); left radius-ulna (NMBE5033621); left semilunate and scaphoid (NMBE5033627, NMBE5033628); right unciform (NMBE5033623); proximal fragment of right McIII (NMBE5033613); left McIV (NMBE5014497); all specimens from Bumbach (Bern Canton, Switzerland), dated from MP25 (figures 2 and 3).

**Description:** The material from Bumbach is poorly preserved, sliced and crushed. Both sides are preserved on the mandible NMBE5033614 (figure 2*a–e*). Only p3 and a part of m3 are preserved on the left side, whereas p1–3 and labial parts of p4–m2 are preserved on the right side. The symphysis is broken, but it is nonetheless upraised compared with the *corpus mandibulae*. The symphysis is quite wide, and its posterior border is located at the level of p2. As on the previously described mandible NMB.O.B.928 from Rheinbetts, there are several mental foramina on each side. On the right side, two large ones are at the level of p1/2 and p3 and a much smaller one is between p1 and i2. On the left side, three large foramina are at the level of p1/2 and a smaller one below p2. However, the symphysis is very badly preserved, and some parts are missing. The lingual groove for the mylohyoid nerves and vessels seems to be well marked on the corpus, but it could also be an artefact from the restoration. The ventral base of the *corpus mandibulae* is straight. On the other mandible available, NMBE5033615 (figure 2*e,f*), only the right part is preserved, with p4 and a part of m1. The symphysis is broken, but the ramus is preserved, and it is inclined forward and upward. The coronoid apophysis is broken and the *foramen mandibulare* is located below the teeth neck. The premolar series seems long compared to the molar series, but it is difficult to estimate precisely the exact length of the latter. Cement is completely absent. Only the root of one i2 is partly preserved on NMBE5033614 (figure 2*a*–*e*). Based on the fragmentary and heavily crushed remaining part of the symphysis, i1s were probably absent. The third incisor and the canine are also absent. The p1 is present, large and single-rooted. There are very weak vertical rugosities on the anterior part of the ectolophid of p2–3. The ectolophid groove of the cheek teeth is deep to angular and does not vanish above the neck on m2 but does on premolars. The trigonid is angular and forms an acute angle in occlusal view. The opening of the posterior valley is acute on premolars, and the lingual cingulid is very weak, but present below the anterior valley. Labial cingulid is present on the trigonid, and below the ectolophid groove. The paralophid of p2 is curved, without constriction, and developed. The posterior valley of p2 is lingually open. The labial cingulid is present on the trigonid of m3. No other characters are visible on the molars.

The postcranial elements are slender and small-sized (figure 3 and tables 2–4). The atlas NMBE5033616 (figure 3*e*,*f*) is partly preserved, lacking the processus transverse (Ltot: (115.5); Htot: 65.0; H *foramen vertebrale*: 47.0; W condylar facets: 83.5). The rachidian canal has a mushroom-like outline. The alar notches are seemingly absent, while the *foramen vertebrale lateralis* and the *foramen transversarium* are present. They are hidden by the axis-facets in lateral view. The condylar facets are kidney-shaped, while the axis facets are transversally straight. The dimensions of the body (APD: 31.0; TDant: 35.5; TDpost: 37.0; Hant: 40.0; Hpost: 40.0) and the vertebral foramen (TD: 22.0; H: 23.0) of the thoracic vertebra NMBE5033617 (figure 3*g*) fit well those of the atlas NMBE5033616. Only the spinous process is observable from the deformed and incomplete thoracic vertebra NMBE5033618 (figure 3*h*).

**Table 2.** Measurements of the lower teeth of the Oligocene rhinocerotine *Mesaceratherium tschani* sp. nov. from Bumbach, compared with other *Mesaceratherium* species. Measurements are given in millimetres and are presented as length/width.

teeth	*Mesaceratherium tschani* sp. nov. NMBE5033615	*Mesaceratherium tschani* sp. nov. NMBE5033614	*Mesaceratherium gaimersheimense* (from Gaimersheim)	*Mesaceratherium gaimersheimense* (from Thézels)	*Mesaceratherium welcommi*
**p1**		16/8.5	14/x	15.3–17/8–9.6	
**p2**		23/17.5	22–23/12–14	20.6–23.6/11.8–15.5	
**p3**		27.5–27.5/19.5–21.5	27–30.5/18.5–20.5	27–30.3/19.4–22	25/22.5
**p4**	31.5/21	31/x	28–30.5/20–22.5	27.6–33/21–23.9	32/30.5–32
**m1**			29–34.5/22–26.5	30–33.7/20.5–24	31–41/27.5–33
**m2**		(>30)/x	32–36.5/21.5–26	31.5–38/23–26	39.5–49.5/29.5–32
**m3**		(>35)/(25)	34/22.5	33–39/21.3–22.7	46.5–53.5/26–27.5

**Table 3.** Measurements of the long bones and metacarpals of the Oligocene rhinocerotine *Mesaceratherium tschani* sp. nov. from Bumbach compared with other *Mesaceratherium* species, in millimetres.

Postcranials	L	TDprox	APDprox	TDdia	APDdia	TDdist	APDdist
**Humerus** NMBE5033619 (*Mesaceratherium tschani* sp. nov.)				39.5	44	81	75.5
**Humerus** NMBE5033620 (*Mesaceratherium tschani* sp. nov.)				38	41	88	(66.5)
**Humerus** from Thézels (*Mesaceratherium gaimersheimense*)	298.1	109.8		39.2–49.9	44.7–49.4	82.2–99.1	68–73.2
**Radius** NMBE5033622 (*Mesaceratherium tschani* sp. nov.)	258.0	66.0	32.0	42.0	22.0	52.0	39.0
**Radius** NMBE5033621 (*Mesaceratherium tschani* sp. nov.)	238.5	>57.0		40.5	25.0		>37.0
**Radius** from Thézels (*Mesaceratherium gaimersheimense*)		60.9–70	34.7–38.2			70.8	
**Radius** from the Bugti Hills (*Mesaceratherium welcommi*)						80	(53)
**Ulna** NMBE5033621 (*Mesaceratherium tschani* sp. nov.)	326.0	40.5	109.5	18.0	37.0		
**Ulna** The4338 from Thézels (*Mesaceratherium gaimersheimense*)			112.6	25.2	46.5		
**McIII** NMBE5033613 (*Mesaceratherium tschani* sp. nov.)	>133.0	(42.0)	>27.0	33.5	12.0		
**McIII** from Thézels (*Mesaceratherium gaimersheimense*)	153–172.9	32.1–40.5	22.3–32	30.2–37.8	11.8–15	40.3–50.7	25–29.5
**McIII** from Laugnac (*Mesaceratherium paulhiacense*)	(192)	(28)	(40)	(39)	(16)	(45)	(33)
**McIV** NMBE5014497 (*Mesaceratherium tschani* sp. nov.)	135.0	29.5	27.0	19.5	11.0	23.5	24
**McIV** The4117 from Thézels (*Mesaceratherium gaimersheimense*)		23	31.7				
**McIV** from Laugnac (*Mesaceratherium paulhiacense*)	(153)	(31)	(39)	(24)	(17)	(35)	(32)

**Table 4.** Measurements of the carpal bones of the Oligocene rhinocerotine *Mesaceratherium tschani* sp. nov. from Bumbach compared with other *Mesaceratherium* species, in millimetres.

Postcranials	APD	TD	H
**Scaphoid** NMBE5033628 (*Mesaceratherium tschani* sp. nov.)	43	32.5	33.5
**Scaphoid** from Gaimersheim (*Mesaceratherium gaimersheimense*)	53.9	34.5	42.6
**Scaphoid** from Thézels (*Mesaceratherium gaimersheimense*)	55.5–62.1	30.8–35.3	48.6–54.7
**Scaphoid** from the Bugti Hills (mean) (*Mesaceratherium welcommi*)	63	41.7	64
**Scaphoid** from Laugnac (*Mesaceratherium paulhiacense*)	(56)		(59)
**Unciform** NMBE5033623 (*Mesaceratherium tschani* sp. nov.)	48.8	45.5	32
**Unciform** BSPG-1952-II from Gaimersheim (*Mesaceratherium gaimersheimense*)	50.5	47	37.5
**Unciform** from Thézels (*Mesaceratherium gaimersheimense*)	60.4–65.4	42.6–50.9	34.8–42.8
**Unciform** from the Bugti Hills (mean) (*Mesaceratherium welcommi*)	63	59.5	54.5
**Unciform** from Laugnac (*Mesaceratherium paulhiacense*)	(47)	(47)	(54)
**Lunate** NMBE5033627 (*Mesaceratherium tschani* sp. nov.)	>40.0	30.0	36.0
**Lunate** BSPG-1952-II from Gaimersheim (*Mesaceratherium gaimersheimense*)	39.6	29.2	31.1
**Lunate** from Thézels (*Mesaceratherium gaimersheimense*)	57.9–67.9	32.6–40.6	40.8–44.7
**Lunate** from the Bugti Hills (*Mesaceratherium welcommi*)	61.5	(>36)	50
**Lunate** from Laugnac (*Mesaceratherium paulhiacense*)	(53)	(38)	(48)

Two distal fragments of humerus (NMBE5033619 and NMBE5033620; figure 3*a*–*d*) are referred to this taxon. The diaphysis is slender. The humeral crest forms a right dihedron with the epicondylar crest. The *fossa olecrani* is narrow and high. The trochlea is poorly constricted in its median part (egg cup *sensu* [27]). The medial lip is more developed. The lateral epicondyle is rather low, moderately developed and bearing a shallow distal gutter. There is no scar (synovial fossette) on the anteroproximal part of the trochlea.

The radius is represented by two deformed specimens, but nearly complete. NMBE5033621 (figure 3*i*) includes also the proximal part of the ulna. The latter is not completely in anatomical connection, the radius being rotated by about counter-clockwise 90^o^ and the effective welding is due to taphonomical process. However, it shows clear synostosis traces resulting from a contact radius/ulna. From the radius NMBE5033621 (figure 3*j*), the anterior border of the proximal articulation is straight, the medial border of the diaphysis is rather straight, the *m. extensor carpi* groove is wide and deepened by the strong *tuberculum dorsale* lying beside it, and the posterior expansion of the scaphoid-facet is low. On NMBE5033622, the proximal ulna-facets are clearly separate and the insertion of the *m. biceps brachii* is shallow. From the ulna, the olecranon is thick, forming a rather closed angle with the diaphysis. The posterior tip of the olecranon (insertion of the *m*. *triceps brachii*) is salient with respect to the distal border of the process. By its dimensions, the humeral cochlea fits the distal trochlea of the humerus NMBE5033620.

The carpus is slender. Both the left scaphoid (NMBE5033628; figure 3*k*,*l*) and lunate (NMBE5033627; figure 3*m*,*n*) are posteriorly incomplete but they fit together with the radius-ulna (NMBE5033621) and belong probably to the same individual. The posteroproximal facet of the scaphoid for the lunate is lacking, but the two bones are posteroproximally in contact. The trapezium-facet and the magnum-facet are not visible from the scaphoid. The lunate has no ulna-facet. The anterior side is smooth, with a rounded distal border. The magnum-facet does not reach the anterior side. In lateral view, the distal pyramidal-facet is elliptic and anteriorly elongated by a thin band stretching to the anterior border. The unciform-facet is sagittally elongated.

The unciform NMBE5033623 (figure 3*o*,*p*) is well preserved. The proximal facets are separated by an acute ridge. The semilunate-facet roughly outlines a quarter-circle nearly flat. The pyramidal-facet is larger, slightly concave transversally and regularly convex sagittally. There is a wide posterolateral expansion to this facet, which connects it to the McV-facet. The distal facets (for the magnum, McIII and McIV) are not distinct, except the most lateral one, for the McV. In anterior view, the laterodistal border of the bone is straight (McV-facet) while the rest of the distal border is rounded. This McV-facet is subvertical pointing to a tridactyl manus.

The proximal fragment of McIII NMBE5033613 (figure 3*q*,*r*) points to a very slender bone. In dorsal view, the lateral articular facets fit with the McIV NMBE5014497, which leads us to assign it to the same individual. The insertion of the *m. extensor carpalis* is salient and the magnum-facet is largely visible in anterior view. The latter is regularly concave transversally and separate from the unciform-facet by a sharp ridge (90° angle). The proximal end is wide, due to the strong lateral development of the unciform-facet. The latter is wide, slightly sagittally convex and triangular. The surfaces for the *m. interossei* are restricted to the proximal half of the diaphysis on the medial and lateral sides. The McIV-facets are well developed and separate. The anterior one is elliptic and the posterior one is circular. It is distally displaced with respect to the proximal end of the bone. The diaphysis is straight and anteroposteriorly flattened. The McII-facet is not observable on the available specimen.

The McIV NMBE5014497 (figure 3*s*,*t*) is slender (TDdia/L = 0.144). Its proximal facet is pentagonal and sagittally elongated in proximal view. On the medial side, the McIII-facets are separated by a deep groove. The anterior one is elliptic and subhorizontal, while the posterior one is circular and lower. The diaphysis is curved laterally, at the proximal third of the bone. The *m. interosseus* is restricted to the proximal half of the diaphysis. The diaphysis is slightly sagittally flattened. The intermediate relief is high and acute. The medial lip is lower than the lateral one. The latter is transversally concave while the former is flat. In lateral view, the proximal end bears a small articular McV-facet.

**Comparisons.** As for the specimen from Rheinbetts, these specimens from Bumbach can be unambiguously attributed to a rhinocerotid, based on the typical shape of the lower cheek teeth (ectolophodont cheek teeth with a *cristid obliqua* directed towards the protoconid) and the anterior dentition (absence of i3 and c, and tusk-shaped i2). It can also be further referred to a small to medium-sized rhinocerotid, thus excluding large-sized European Oligocene rhinoceroses such as *Ronzotherium* or *Diaceratherium* [4,16,17,22].

Among the six genera of medium-sized Rhinocerotidae known in Europe during the Oligocene–Early Miocene interval, *Pleuroceros* differs by a nearly horizontal mandibular symphysis (upraised on NMBE5033614), a smooth and U-shaped external groove on lower cheek teeth (deep to angular on NMBE5033615 and NMBE5033614) and a continuous lingual cingulid on lower premolars (reduced on NMBE5033615 and NMBE5033614) [25]. *Plesiaceratherium* differs by premolars with shallow ectolophid groove (deep to angular on NMBE5033615 and NMBE5033614) [27,34]. *Protaceratherium* differs by the narrower symphysis in dorsal view (wider on NMBE5033614), the smoother ectolophid groove of the cheek teeth (deep to angular on NMBE5033615 and NMBE5033614) and the stronger cingulid (usually absent on NMBE5033615 and NMBE5033614) [19,35]. *Molassitherium albigense* (lower dentition unknown for ‘*M.*’ *delemontense*) differs by the much stronger cingulid on the lower cheek teeth (usually absent on NMBE5033615 and NMBE5033614) [17,19]. *Epiaceratherium* differs by a posterior valley usually closed on p2 (open on NMBE5033614) and the absence of lingual cingulid on the lower premolars (present on NMBE5033614) [12]. In agreement with our phylogenetic results, we refer the material from Bumbach to *Mesaceratherium*. *Mesaceratherium* shares with these specimens a lingual cingulid on lower premolars, an angular ectolophid groove of the cheek teeth and a rather upraised mandibular symphysis [16,22,25,37]. Furthermore, the postcranial remains from Thézels assigned to *M. gaimersheimense* also share with the specimens from Bumbach the absence of scar on the trochlea, the egg-cup-shaped trochlea and the high *fossa olecrani* of the humerus, the straight anterior border of the proximal articulation and medial border of the epiphysis of the radius, the closed angle between the olecranon of the ulna and the diaphysis, the triangular facet for the unciform on the McIII, and the overall similar dimensions (tables 3 and 4) but differ by other characters, such as a possibly tetradactyl manus, the remote pyramidal and McV facets on the unciform or the keeled anterior side of the lunate [22]. Therefore, based on this unique combination of characters, we refer to these specimens from Bumbach as *Mesaceratherium tschani* sp. nov.

## 3. Results

### 3.1. Phylogeny

The different sets of taxonomic samples used during the phylogenetic analyses and their results are reported in table 5. The resulting trees of each analysis are presented in electronic supplementary material, S2.

**Table 5.** Taxonomic sample used for each analysis, with details on the search algorithms used and a summary of corresponding results. The resulting most parsimonious trees, or consensus trees are available in electronic supplementary material, S2, where page numbers correspond to the analysis number in the first column.

analysis	search algorithm	taxonomic samples	results
1	branch-and-bound	MHNT-PAL-2010-18-1; FSL-8543; FSL-8486; FSL-8544; specimens from Bumbach (one single terminal), NMB.O.B.928	all specimens from Moissac and St-Henri/St-André can be referred to *Molassitherium albigense*. NMB.O.B.928 should be referred to *Epiaceratherium.*
2	branch-and-bound	Addition of *Mesaceratherium gaimersheimense* and *M. welcommi*	all specimens from Moissac and St-Henri/St-André can be referred to *Molassitherium albigense*. NMB.O.B.928 should be referred to *Epiaceratherium magnum.*
3	branch-and-bound	Addition of *Pleuroceros pleuroceros* and *P. blanfordi*	all specimens from Moissac and St-Henri/St-André can be referred to *Molassitherium albigense*. NMB.O.B.928 can be referred to *Epiaceratherium magnum*. Bumbach closer to *Pleuroceros*/*Mesaceratherium.*
4	branch-and-bound	**MHNT-PAL-2010-18-1,** **FSL-8543, FSL-8486 and FSL-8544 merged (=*M. albigense*)**	*M. delemontense* not sister group of *M. albigense*. Bumbach closer to *Pleuroceros*/*Mesaceratherium.*
5	branch-and-bound	**NMB.O.B.928 and *Epiaceratherium magnum* merged**	*M. delemontense* not sister group of *M. albigense*. Bumbach closer to *Pleuroceros*/*Mesaceratherium.*
6	branch-and-bound	Addition of *Uintaceras radinskyi*	*M. delemontense* not sister group of *M. albigense*. Bumbach closer to *Pleuroceros*/*Mesaceratherium.*
7	branch-and-bound	Addition of *Teletaceras radinskyi*	*M. delemontense* not sister group of *M. albigense*.
8	branch-and-bound	Addition of *Penetrigonias dakotensis*	*M. delemontense* not sister group of *M. albigense.*
9	branch-and-bound	Addition of *Trigonias osborni*	*M. delemontense* not sister group of *M. albigense.*
10	heuristic	addition of *Amphicaenopus platycephalus*	*M. delemontense* not sister group of *M. albigense*. Bumbach closer to *Pleuroceros*/*Mesaceratherium.*
11	heuristic	addition of *Subhyracodon occidentalis*	*M. delemontense* not sister group of *M. albigense*.
12	heuristic	addition of *Protaceratherium minutum*	*Epiaceratherium* monophyletic (including *M. delemontense*) sister-group of *Teletaceras* and *Uintaceras*. specimens from Bumbach sister-group of *Protaceratherium* and *Pleuroceros*
13	heuristic	addition of *Diceratherium armatum*	specimens from Bumbach sister-group of *Mesaceratherium*
14	heuristic	addition of *Ronzotherium filholi*	
15	heuristic	addition of *Aceratherium incisivum*	Bumbach sister-group of *Aceratherium*
16	heuristic	addition of *Teleoceras fossiger*	Bumbach sister-group of *Aceratherium*
17	heuristic	addition of *Brachypotherium brachypus*	Bumbach sister-group of *Mesaceratherium*
18	heuristic	addition of *Prosantorhinus douvillei*	Bumbach sister-group of *Mesaceratherium*
19	heuristic	addition of *Alicornops simorrense* and *Plesiaceratherium platyodon*	
20	heuristic	addition of *Lartetotherium sansaniense*	Bumbach sister-group of *Mesaceratherium*
21	heuristic	addition of *Mesaceratherium paulhiacense*	*Mesaceratherium* monophyletic, including Bumbach

Analyses 1–5 were done in order to test different hypotheses:

(1) whether the specimens from St-Henri/St-André and Moissac (described by Lihoreau *et al*. [38]) all belonged to *Molassitherium albigense*, which our analyses have confirmed;
(2) whether *Molassitherium* and *Epiaceratherium* were monophyletic in their previous acceptation; however, here the results cannot support their monophyly, but a larger taxonomic sampling is necessary to support this claim;
(3) whether the specimen NMB.O.B.928 from Rheinbetts could be confidently referred to *Epiaceratherium magnum*, which has been confirmed by the analyses;
(4) whether the specimens from Bumbach could be referred to *Molassitherium* (as suggested by Scherler *et al*., 2013: Online Resource 1), which has been partly refuted by the results.

Thus, more taxa were needed to test at least two hypotheses: monophyly of *Molassitherium* and *Epiaceratherium* and refined taxonomic assignment of the specimens from Bumbach. To test them, we added six early-branching genera of Rhinocerotidae (*Uintaceras, Teletaceras, Penetrigonias, Trigonias, Amphicaenopus* and *Subhyracodon*) in order to stabilize the tree base, through the analyses 6–11. Based on the results of these analyses, it became clear that *Molassitherium* as originally refined was not monophyletic. However, no referral to known species can be proposed for the specimens from Bumbach based on these analyses.

**Figure 4.**
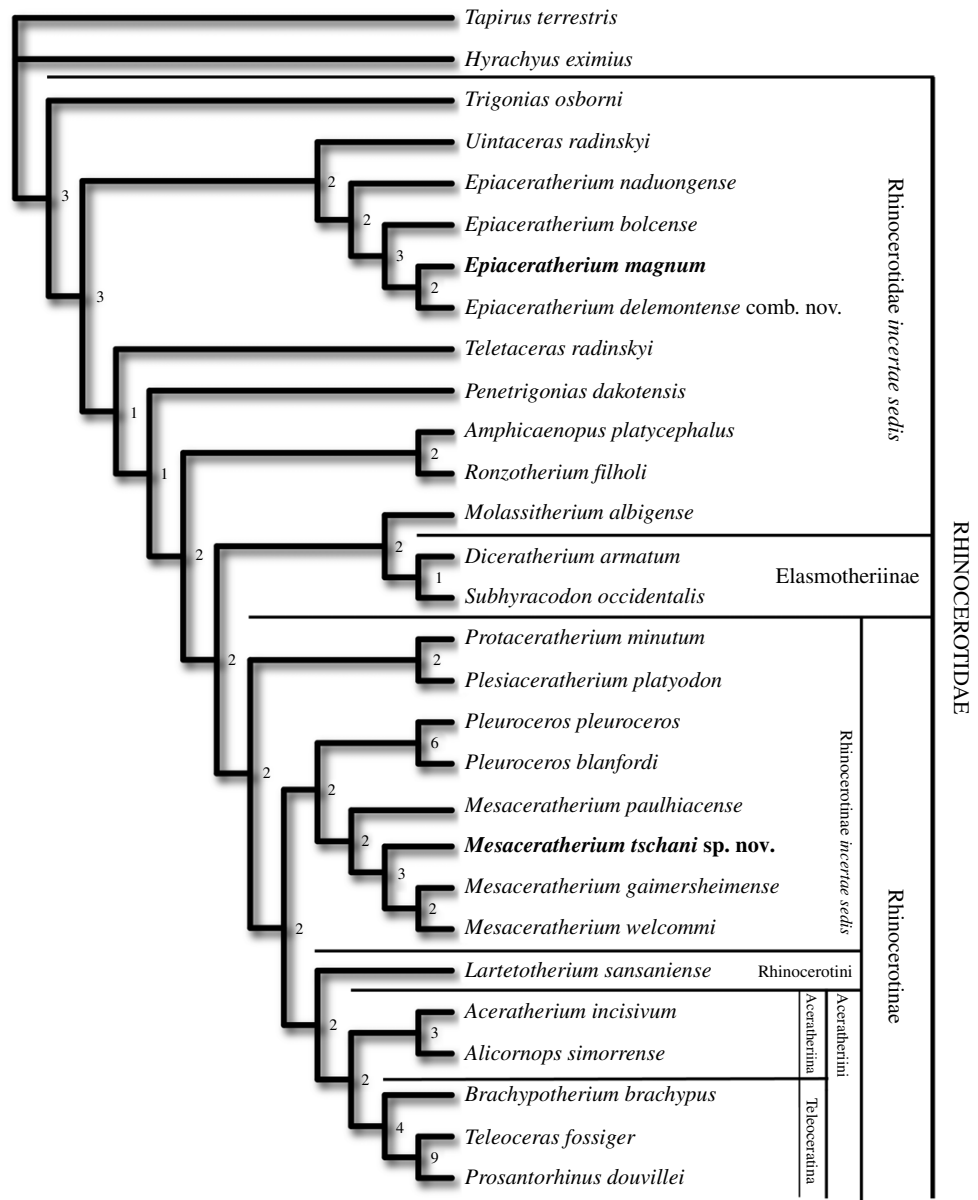
Single most parsimonious tree of the Rhinocerotidae using the heuristic search in PAUP* v. 4.0a (build 167) with a matrix of 288 morphological characters and 28 terminals, with *Tapirus terrestris* and *Hyrachyus eximius* as outgroups. Tree length = 1099 steps, RI = 0.43, CI = 0.3. Bremer support values are reported at the nodes. Newly described specimens from Rheinbetts and Bumbach are attributed to *Epiaceratherium magnum* and *Mesaceratherium tschani* sp. nov., respectively.

We then added several more derived rhinocerotid taxa to further test the non-monophyly of *Molassitherium* and get a stable placement for the specimens from Bumbach, which tended to branch close to *Pleuroceros* and/or *Mesaceratherium* without clear preference. Thus, during analyses 12 to 20, nine new genera were added, comprising Oligocene to Miocene genera of Rhinocerotidae, representing two subfamilies: Elasmotheriinae and Rhinocerotinae (comprising Rhinocerotini and Aceratheriini [Aceratheriina + Teleoceratina]) as well as *Ronzotherium*, an early large-sized rhinocerotid. Finally, the lesser known species *Mesaceratherium paulhiacense* was added to further test the monophyly of *Mesaceratherium*, and the placement of the specimens from Bumbach. Based on these results, these specimens are attributed to *Mesaceratherium tschani* sp. nov. This is the placement resulting from most of our analyses, including the most comprehensive ones, and for which the topology of tree is the most stable. Thus, adding more taxa would now be unnecessary, although it could lead to new topologies. Indeed, our identifications would probably not drastically change as they are well supported by numerous characters. The definitive tree (single most parsimonious tree) resulting from the most complete taxonomical sampling is presented in figure 4.

**Figure 5.**
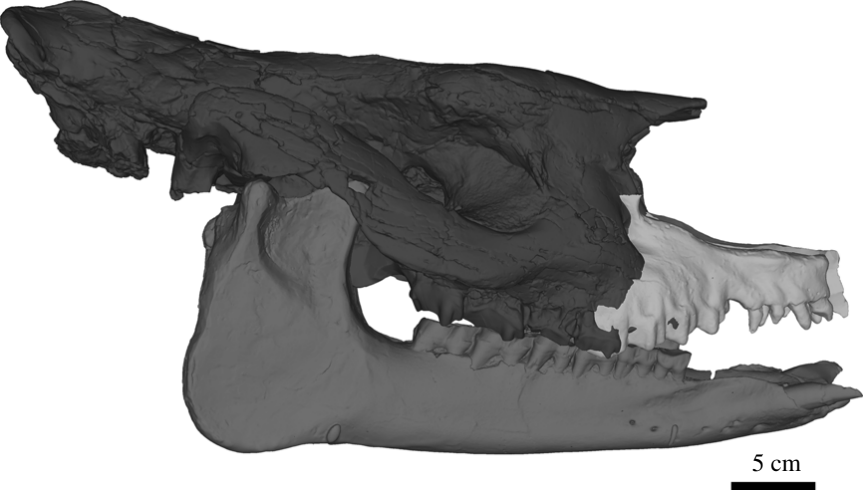
Archetypic reconstruction of the skull of *Epiaceratherium*, generated by three-dimensional virtual association of the cranium of *E. delemontense* comb. nov. (in dark grey), mandible of *E. magnum* (in medium grey) and snout of *E. bolcense* (in light grey). Three-dimensional model available in Tissier, Antoine & Becker (2020) [26].

Our new phylogeny leads to an important change concerning the monophyly of *Molassitherium*, which cannot be supported by our results. Indeed, the species ‘*M*. *delemontense* Becker & Antoine, 2013 [19]’ should be assigned to *Epiaceratherium* instead. *Molassitherium delemontense* was erected on a very well-preserved skull from Poillat (Jura Canton, Switzerland), which shared numerous similarities with the skull of *Molassitherium albigense*. However, based on our results, it shares more characters with *Epiaceratherium* than with *Molassitherium*, such as: a *foramen infraorbitalis* above P3 (**char. 2**), as in *E. bolcense*, but also *M. albigense*; a sharply deviating anterior tip of the zygomatic process in ventral view (**char. 37**), as in *E. bolcense*, but contrary to *M. albigense*; a long premolars series compared to the molars (**char. 63**), a synapomorphy of all epiaceratheres, also found in *M. albigense*; the absence of labial cingulum on upper premolars (**char. 83**) and on upper molars (**char. 109**), contrary to *M. albigense*; the hypocone connected to the ectoloph of P2–4 (**char. 86**), as in *E. magnum*, but also *M. albigense*; the separate protocone and hypocone on P2 (**char. 94**), as in *E. magnum*, but also *M. albigense*; the transverse metaloph on P2 (**char. 95**), a synapomorphy of all epiaceratheres and *Uintaceras*, not found in *M. albigense*; a lingual wall on P3–4 (**char. 102**), a synapomorphy of *Epiaceratherium* and *Uintaceras*, that may also be found in *M. albigense*; a hypocone anterior to the metacone on P3–4 (**char. 103**), a character found in all early-branching Rhinocerotidae, but not in *M. albigense*; a continuous metaloph on P4 (**char. 108**), as in *E. magnum*, but also *M. albigense*; an antecrochet always present on upper molars (**char. 110**), a synapomorphy of all *Epiaceratherium* species, that is not always found in *M. albigense*; the lingual cingulum usually absent on upper molars (**char. 114**), a synapomorphy of *Epiaceratherium*, whereas it is usually present in *M. albigense*; a constriction of the protocone always present on M1–2 (**char. 115**), as in *E. bolcense* and *E. magnum*, which is more often absent on *M. albigense*; the absence of metacone fold on M1–2 (**char. 119**), as in *E. magnum*, but also *M. albigense*; a long metastyle on M1–2 (**char. 120**), as in *E. bolcense* and *E. magnum*, but also *M. albigense*; a posterior part of the ectoloph of M1–2 concave (**char. 122**), as in *E. magnum*, but also *M. albigense*; the presence of a lingual groove on the hypocone of M2 (**char. 129**), as in *E. naduongense* and *E. magnum*, but also sometimes on *M. albigense*; the presence of a mesostyle on M2 (**char. 130**), as in *E. bolcense* and *E. magnum*, but also *M. albigense*; a fused ectoloph on M3 (**char. 133**), as in *E. magnum*, as well as *M. albigense*; a protocone always constricted on M3 (**char. 135**) as in *E. bolcense* and *E. magnum*, but contrary to *M. albigense*; and finally, a strong metacone fold on P2 (**char. 285**) and on P3–4 (**char. 286**), as most early-branching Rhinocerotidae, but contrary to *M. albigense*. Thus, although ‘*M. delemontense*’ shares 12 homoplastic synapomorphies with *Epiaceratherium* that are also found in *M. albigense*, it also shares with *Epiaceratherium* 11 synapomorphies that are not found in *Molassitherium*. Therefore, in agreement with these results, we propose the new combination ‘*Epiaceratherium delemontense* (Becker & Antoine, 2013 [19])’. This could imply that this species would have had, like all epiaceratheres, a complete upper dentition, including three pairs of incisors and one pair of canines. A hypothetical representation of the complete skull of *Epiaceratherium* is shown in figure 5 and was generated by virtually assembling the type cranium of *E. delemontense* comb. nov. (specimen MJSN POI007–245; three-dimensional model available in [39]) to the hypothetical gypsum reconstruction of the snout of *E. bolcense* (NMB.I.O.43), which is based on several specimens (including specimens 27 287 and 27 295 housed at the Museum of Geology and Paleontology of Padua) and the mandible from Rheinbetts (NMB.O.B.928) attributed to *E. magnum* (see Tissier *et al*. [26] for details on the protocol). Thus, this representation does not have any anatomical value, and is strictly theoretical, in the aim of visualizing the speculative cranial morphology of *Epiaceratherium*.

### 3.2. Evolution of the anterior dentition in early Rhinocerotidae

The reduction of the anterior dentition is one of the major adaptative traits of the Rhinocerotidae, which developed much earlier than the horn(s). Indeed, the first horns may have appeared in the American genus *Diceratherium*, from the Early Oligocene [27,40,41] whereas the reduction of the anterior dentition was initiated already in Late Eocene times, notably with *Trigonias*, a well-known genus from North America.

**Figure 6.**
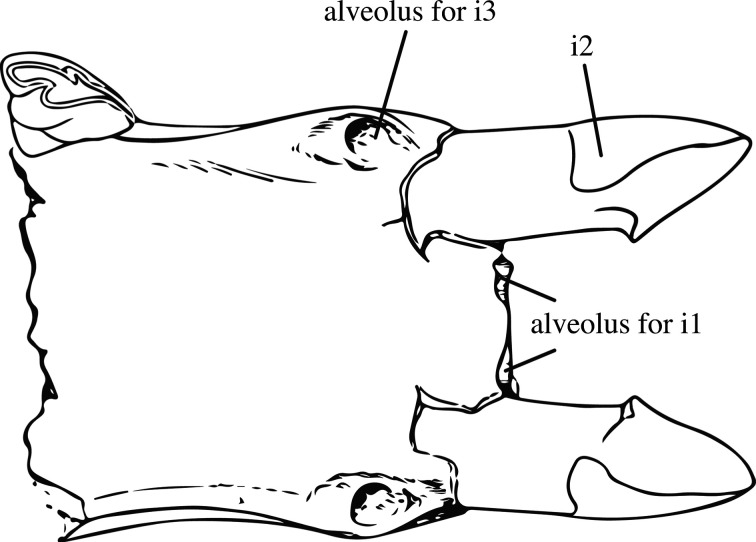
Drawing of the symphysis of the mandible USNM-4815, paratype of *Trigonias osborni*, modified from Lucas [41].

The lower anterior dentition of *Trigonias* has been subject to some confusion. Lucas [41] originally considered the alveolus just behind the tusk-shaped i2 as the alveolus for i3 when he created this genus, but later, Gregory and Cook [42] believed that it could be in fact the alveolus for a canine. Indeed, on one mandible from a juvenile individual (No. 1027), two alveoli are present distally to i2, which indicates the presence of both i3 and a lower canine (or di3 and dc). Based on the shape and position of these alveoli, they suggested that the single tooth retained in the adult was the canine, instead of i3. However, Russell [43] showed that in some specimens the canine was definitely lost in the subadult while the i3 was still present. Thus, the lower canine is lost in the adult, but may still be present in the juvenile. However, Prothero *et al.* [32] and Antoine [27] consider that the lower canine and i3 are lost in the adult *Trigonias* (see [32]: fig. 4, character 28 and [27]: p. 134), following Radinsky [44], who also considered the lower canine and i3 as lost in the adult, and not only in the juvenile. The confusion can perhaps be traced back up to Scott [45] who indicated the presence of three lower incisors in the dental formula of *Trigonias* (p. 776) yet later indicated in the description that ‘*of the third incisor and the canine no vestige remains*' (p. 777), perhaps meaning that these teeth were physically absent, but not their alveoli. On the contrary, Wood [46,47] considered the presence of the lower i3 as a defining generic character, whereas the presence of the lower canine would be variable. Here, we choose to follow Lucas [41] and Wood [46,47] and consider the lower i3 as present in the adult. Indeed, although the paratype mandible USNM-4815 (in Smithsonian National Museum of Natural History) belongs to an old individual with well-worn teeth, as reported by Wood [46], the alveoli for the i3 are still very large and not sealed (figure 6), which indicates that this tooth would have been present during most of the animal's life. However, there may still be some intraspecific variability concerning the presence of this i3, since some individuals reported by Russell [43] show no trace of i3 (fig. 26 for example).

**Figure 7.**
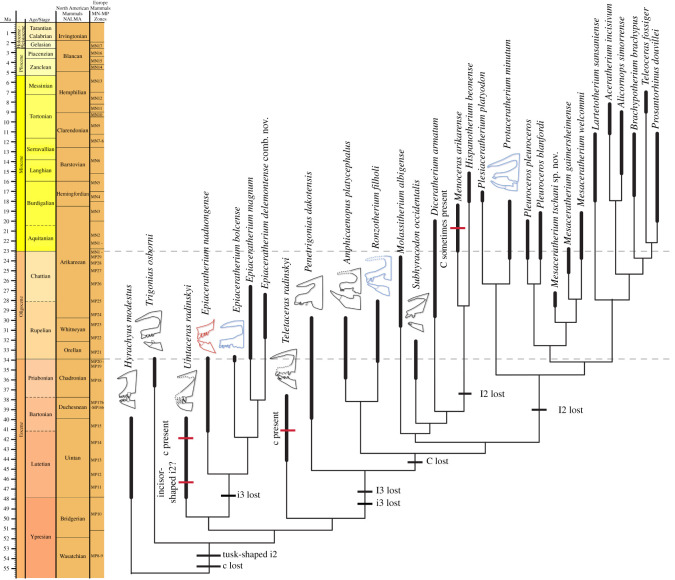
Optimization of characters 73–75, 79 and 81–82 with the ACCTRAN parameter in early-branching Rhinocerotidae, mapped on the single most parsimonious tree. Schematic depictions of anterior dentition are represented next to the taxon names where black lines indicate North American taxa, blue lines indicate European taxa and red lines indicate Asian taxa. Occurrences indicated by bold lines and are based on [1,12,19,25,40,48,49]. *Menoceras arikarense* and *Hispanotherium matritense* have been added to illustrate convergent reduction of anterior dentition among early Elasmotheriinae, and were placed according to previous formal phylogenies [19,25].

The evolution of the major traits concerning upper and lower anterior dentition is optimized on the most parsimonious tree, using the accelerated transformation parameter (ACCTRAN), favouring reversals instead of convergences (figure 7). Thus, according to our phylogeny, the absence of a lower canine can be regarded as a synapomorphy of the Rhinocerotidae, acquired quite early during the evolution of this group (figure 7). Yet, the presence/absence of an upper and/or lower canine remains a rather labile character in this group and may further be subject to sexual dimorphism, as in other laurasiatheres, including perissodactyls. For example, the canine can be present in the adult male domestic horse, but not in the female [50]. Even within rhinoceroses, a rudimentary canine can sometimes be present in some individuals, as observed on one skull of *Dicerorhinus sumatrensis* (copy in MJSN collection). On some skulls of *Subhyracodon*, an upper canine may be present, although only on one side and not the other [40]. We nonetheless coded it as absent for this species, as it is the most-often seen condition. In other groups of perissodactyls, there can be an important sexual dimorphism on the size of the canine (e.g. in Lophiodontidae, as reported by Vautrin *et al*. [51]). Thus, this might explain why the lower canine could have reappeared at least twice during the evolution of the Rhinocerotidae, in *Uintaceras* and *Teletaceras*, for example (figure 7).

Another typical feature of the Rhinocerotidae is the tusk-shaped second lower incisor, which might have been acquired at the same time as the lower canine was lost (figure 7). It is indeed tusk-shaped in all Rhinocerotidae (when it is present), except in *Uintaceras* who might still have retained an incisor-shaped i2 [52]. However, the anterior dentition of this genus remains poorly known, and was not found in connection with the mandible, but the roots preserved in the lower jaw suggest nonetheless that i2 may have been smaller than i3.

*Epiaceratherium* is peculiar, as it possesses a complete upper anterior dentition, but has lost its i3 and c. This differs from the condition seen in all other rhinocerotids, in which I3 is lost at the same time as i3. Thus, i3 has been lost at least twice independently among Rhinocerotidae. I3 is also the first upper anterior tooth lost, before the successive loss of C and I2, and contrary to what occurs in the lower jaw. In *Penetrigonias dakotensis*, I3 is lost while C is unambiguously retained on the anterior tip of the maxilla [40]. However, Russell [43] identified I3 on the premaxilla in *Penetrigonias sagittatus*. Yet, Prothero [40] considers it as a canine. Thus, more investigation would be needed to determine the exact condition in this species. Finally, the loss of I2 is a distinguishing feature of Rhinocerotinae and Elasmotheriini, who retain only the typical chisel-tusk complex formed by I1/i2, as well as a non-functional i1 in many taxa.

## 4. Conclusion

Based on parsimony analyses of Rhinocerotidae, we identify an unpublished mandible (NMB.O.B.928) from the ‘middle’ Oligocene of Rheinbetts (Basel Canton, Switzerland) as belonging to *Epiaceratherium magnum*. As the first complete and well-preserved mandible available for this species, it allows for assessing the absence of i3 and c. Along with this mandible, newly prepared specimens from Bumbach (MP25, early Late Oligocene, Switzerland) are referred to a new species: *Mesaceratherium tschani* sp. nov. These remains document the oldest occurrence of *Mesaceratherium*, which was previously only known from MP28 onward.

Our new phylogenetic analyses support a new combination: *Epiaceratherium delemontense* (Becker & Antoine, 2013 [19]) comb. nov. replaces ‘*Molassitherium delemontense* Becker & Antoine, 2013 [19]’. Optimization of concerned characters further allows for inferring that this species would have retained a complete upper anterior dentition, as in all other species assigned to *Epiaceratherium*. A new evolutionary scenario is proposed for tracking anterior dentition reduction in Rhinocerotidae. Based on this new scenario, the third lower incisor would have been lost at least twice independently, whereas I3 and C would have been lost only once, successively.

## Supplementary Material

Morphological characters matrix

Reviewer comments

## Supplementary Material

Additional trees

## Data accessibility

All data generated or analysed during this study are included in this published article (and its electronic supplementary material files). The morphological data matrix is available in NEXUS format in electronic supplementary material, S1. Three-dimensional models are available from the MorphoMuseuM repository (https://doi.org/10.18563/journal.m3.116) with DOIs https://doi.org/10.18563/m3.sf.534 (three-dimensional surface model of the mandible NMB.O.B.928) and https://doi.org/10.18563/m3.sf.534 (three-dimensional surface model of the archetype reconstruction of *Epiaceratherium*).
