# Supplementary material for: New material of Epiaceratherium and a new species of Mesaceratherium clear up the phylogeny of early Rhinocerotidae (Perissodactyla)
Source: R Soc Open Sci. 2020 Jul 15;7(7):200633. doi: 10.1098/rsos.200633 (PMC7428265; doi:10.1098/rsos.200633)

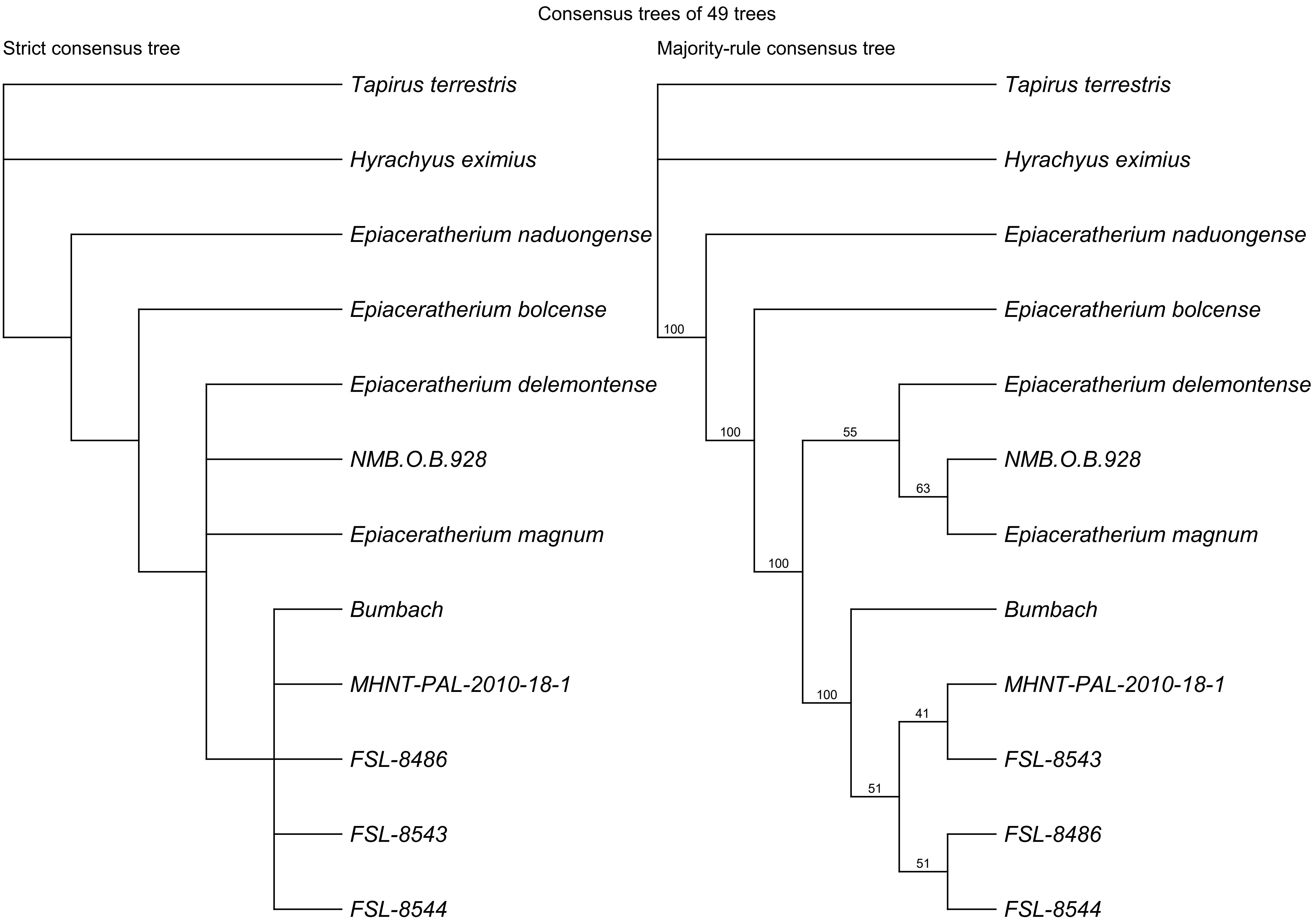

Consensus trees of 49 trees
Strict consensus tree
Majority-rule consensus tree
Tapirus terrestris
Hyrachyus eximius
Epiaceratherium naduongense
Epiaceratherium bolcense
Epiaceratherium delemontense
NMB.O.B.928
Epiaceratherium magnum
Bumbach
MHNT-PAL-2010-18-1
FSL-8486
FSL-8543
FSL-8544
Tapirus terrestris
Hyrachyus eximius
Epiaceratherium naduongense
Epiaceratherium bolcense
Epiaceratherium delemontense
NMB.O.B.928
Epiaceratherium magnum
Bumbach
MHNT-PAL-2010-18-1
FSL-8543
FSL-8486
FSL-8544
100
100
55
63
100
100
41
51
51

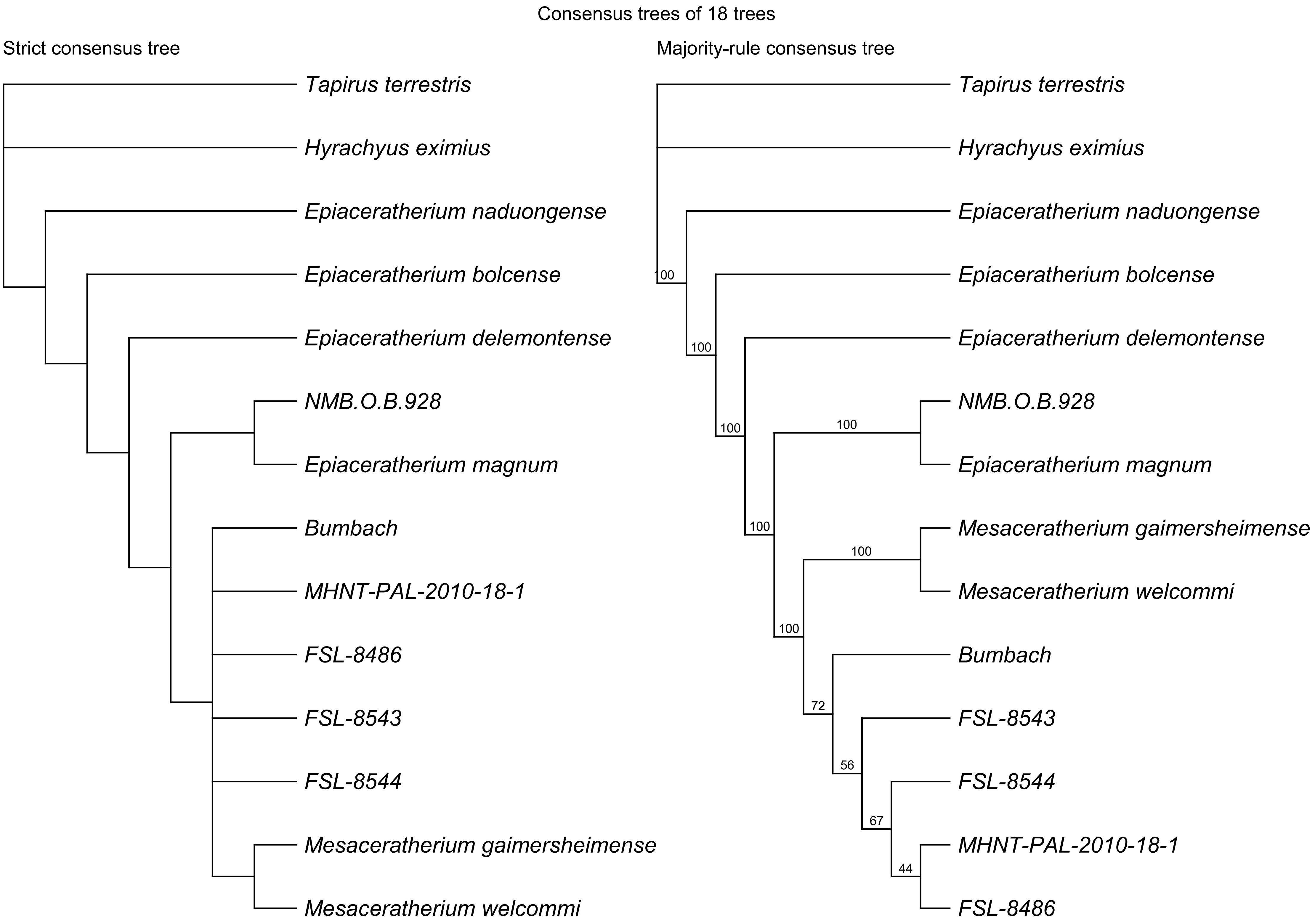

Consensus trees of 18 trees
Strict consensus tree
Majority-rule consensus tree
Tapirus terrestris
Hyrachyus eximius
Epiaceratherium naduongense
Epiaceratherium bolcense
Epiaceratherium delemontense
NMB.O.B.928
Epiaceratherium magnum
Bumbach
MHNT-PAL-2010-18-1
FSL-8486
FSL-8543
FSL-8544
Mesaceratherium gaimersheimense
Mesaceratherium welcommi
100
100
100
100
100
100
100
72
56
67
44

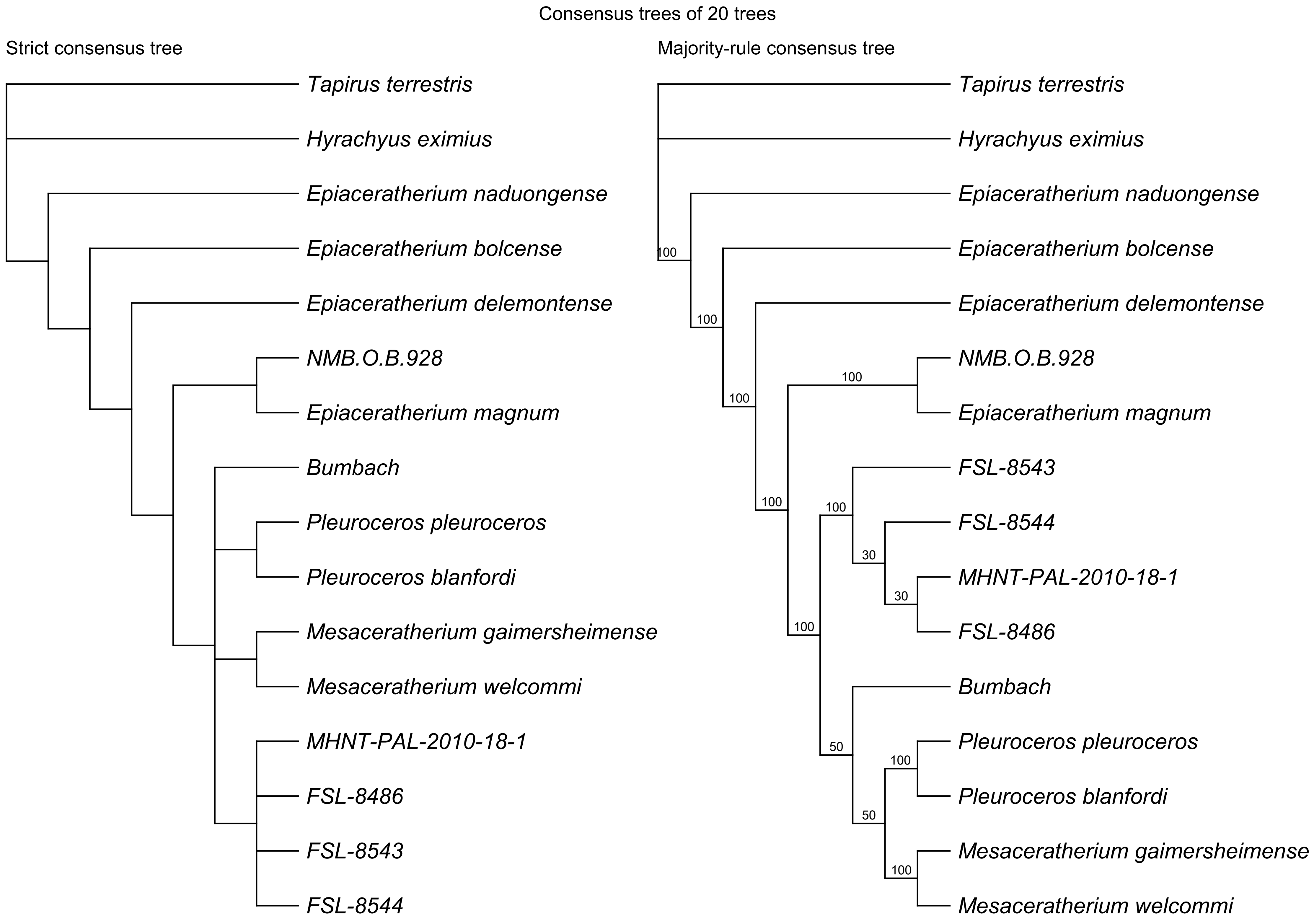

Consensus trees of 20 trees
Strict consensus tree
Tapirus terrestris
Hyrachyus eximius
Epiaceratherium naduongense
Epiaceratherium bolcense
Epiaceratherium delemontense
NMB.O.B.928
Epiaceratherium magnum
Bumbach
Pleuroceros pleuroceros
Pleuroceros blanfordi
Mesaceratherium gaimersheimense
Mesaceratherium welcommi
MHNT-PAL-2010-18-1
FSL-8486
FSL-8543
FSL-8544
Majority-rule consensus tree
Tapirus terrestris
Hyrachyus eximius
Epiaceratherium naduongense
Epiaceratherium bolcense
Epiaceratherium delemontense
NMB.O.B.928
Epiaceratherium magnum
FSL-8543
FSL-8544
MHNT-PAL-2010-18-1
FSL-8486
Bumbach
Pleuroceros pleuroceros
Pleuroceros blanfordi
Mesaceratherium gaimersheimense
Mesaceratherium welcommi
100
100
100
100
100
100
30
30
100
50
100
50
100

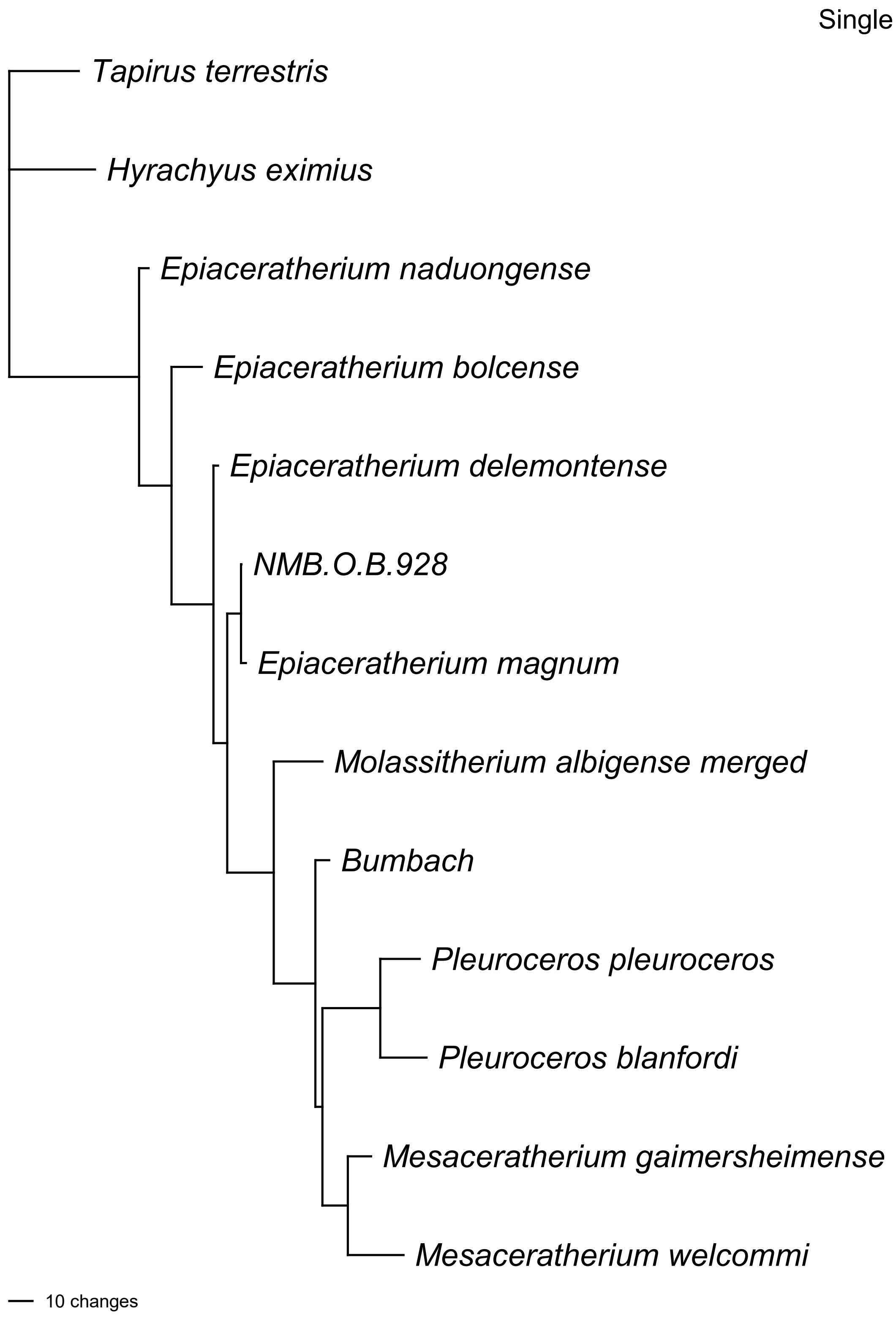

Single most parsimonious tree
Tapirus terrestris
Hyrachyus eximius
Epiaceratherium naduongense
Epiaceratherium bolcense
Epiaceratherium delemontense
NMB.O.B.928
Epiaceratherium magnum
Molassitherium albigense merged
Bumbach
Pleuroceros pleuroceros
Pleuroceros blanfordi
Mesaceratherium gaimersheimense
Mesaceratherium welcommi
10 changes

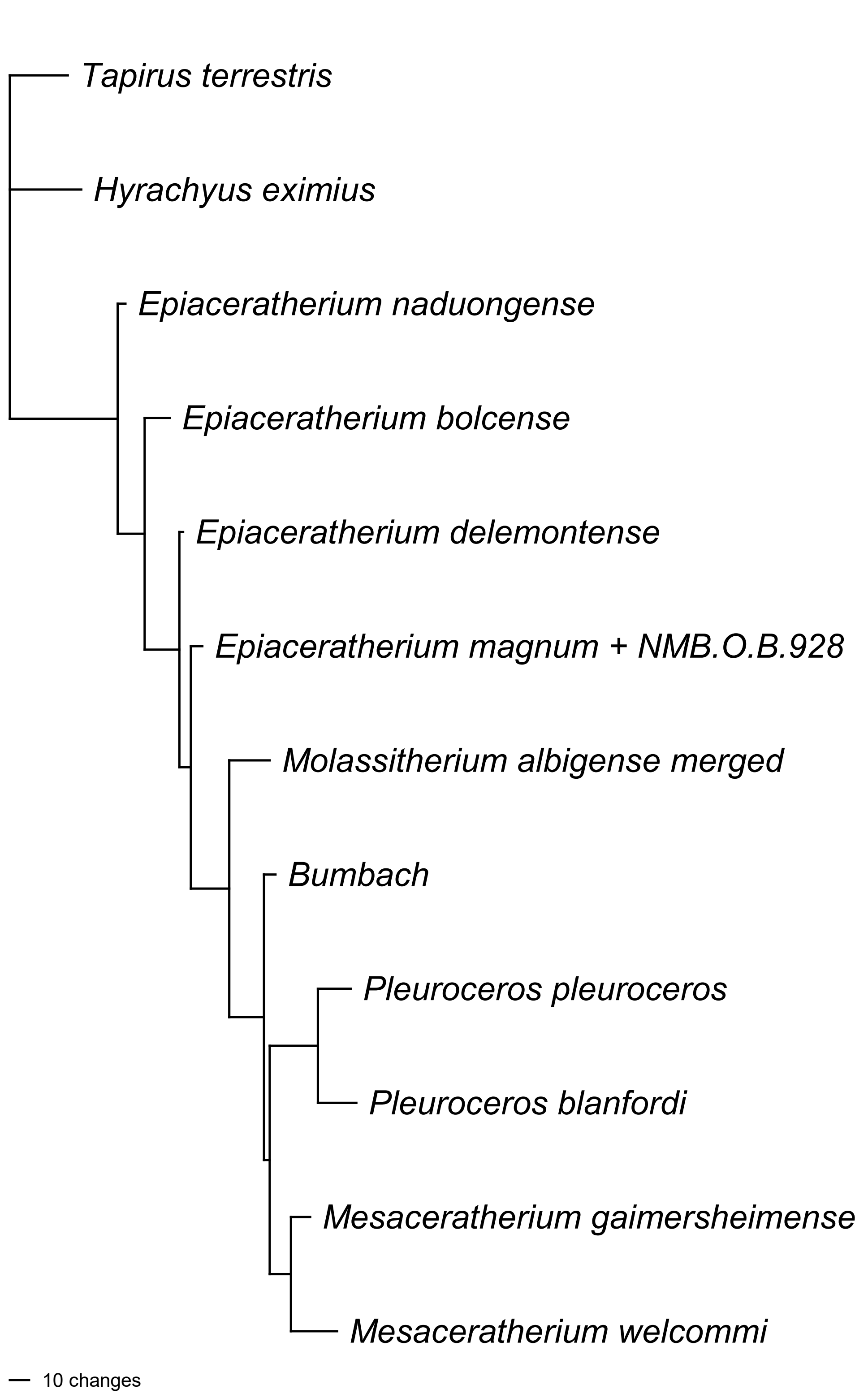
Single most parsimonious tree
Tapirus terrestris
Hyrachyus eximius
Epiaceratherium naduongense
Epiaceratherium bolcense
Epiaceratherium delemontense
Epiaceratherium magnum + NMB.O.B.928
Molassitherium albigense merged
Bumbach
Pleuroceros pleuroceros
Pleuroceros blanfordi
Mesaceratherium gaimersheimense
Mesaceratherium welcommi
10 changes

Single most parsimonious tree

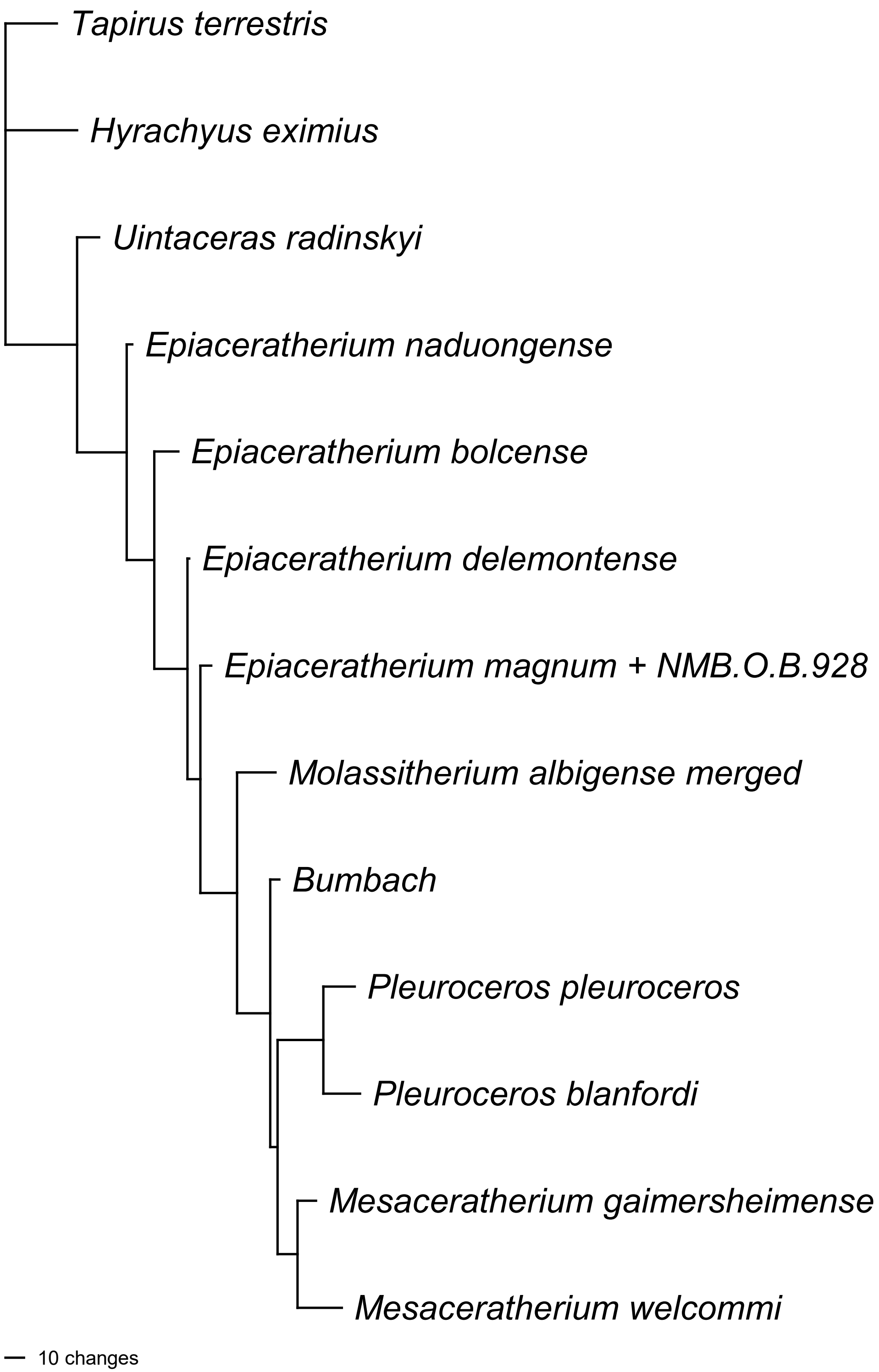

Consensus trees of 2 trees

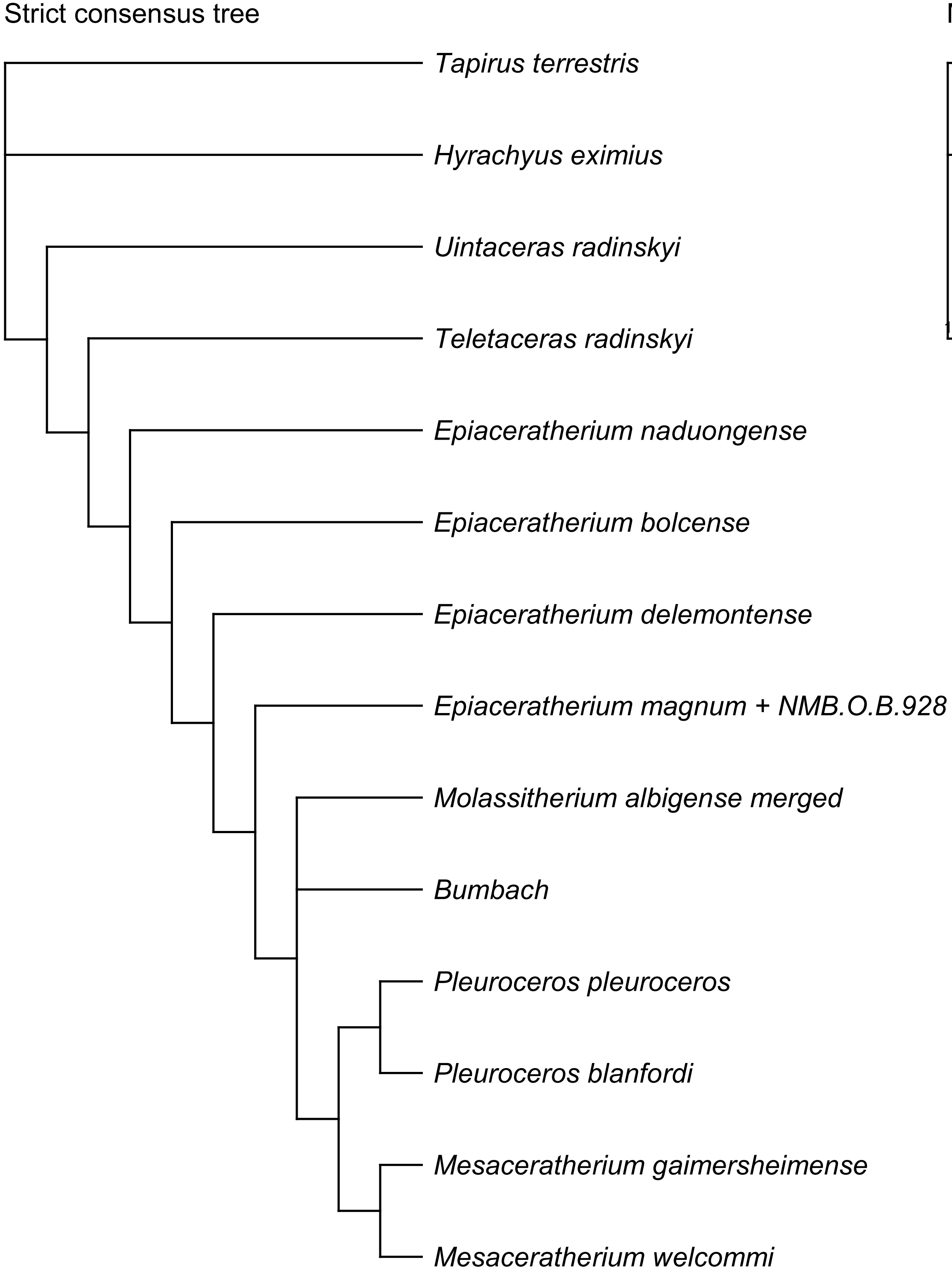

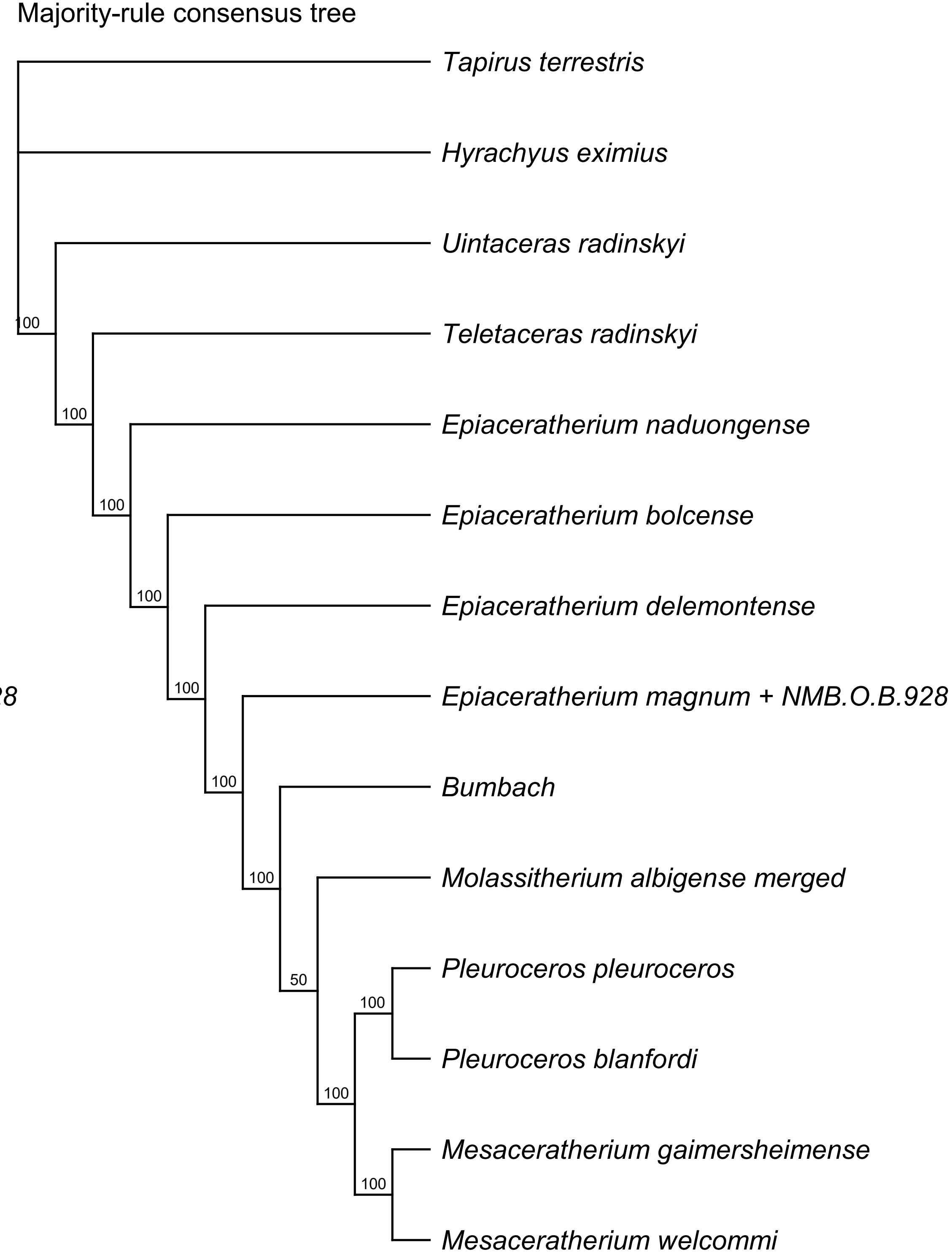

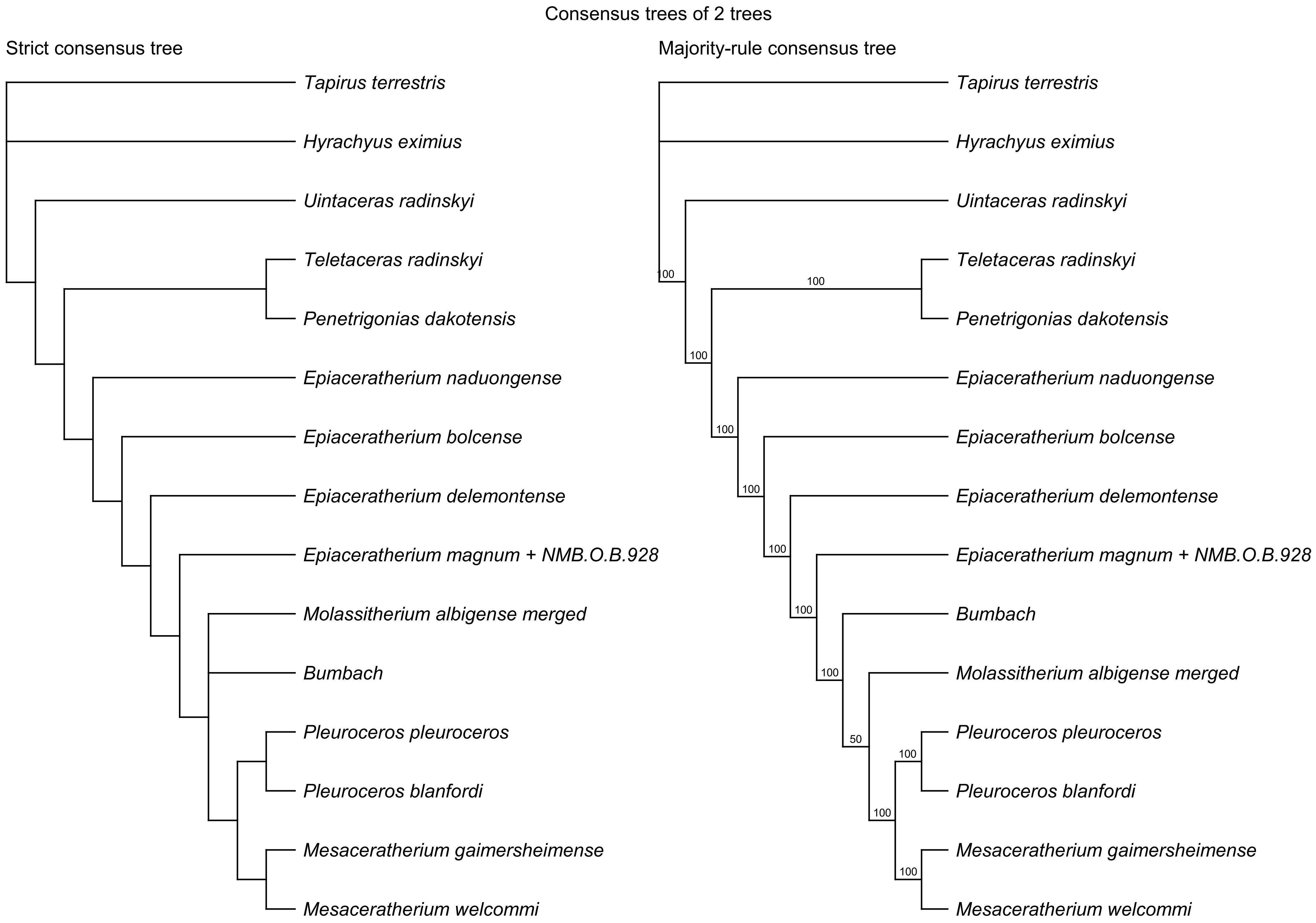
Consensus trees of 2 trees
Strict consensus tree
Tapirus terrestris
Hyrachyus eximius
Uintaceras radinskyi
Teletaceras radinskyi
Penetrigonias dakotensis
Epiaceratherium naduongense
Epiaceratherium bolcense
Epiaceratherium delemontense
Epiaceratherium magnum + NMB.O.B.928
Molassitherium albigense merged
Bumbach
Pleuroceros pleuroceros
Pleuroceros blanfordi
Mesaceratherium gaimersheimense
Mesaceratherium welcommi
Majority-rule consensus tree
Tapirus terrestris
Hyrachyus eximius
Uintaceras radinskyi
Teletaceras radinskyi
Penetrigonias dakotensis
Epiaceratherium naduongense
Epiaceratherium bolcense
Epiaceratherium delemontense
Epiaceratherium magnum + NMB.O.B.928
Bumbach
Molassitherium albigense merged
Pleuroceros pleuroceros
Pleuroceros blanfordi
Mesaceratherium gaimersheimense
Mesaceratherium welcommi
100
100
100
100
100
100
100
100
50
100
100
100

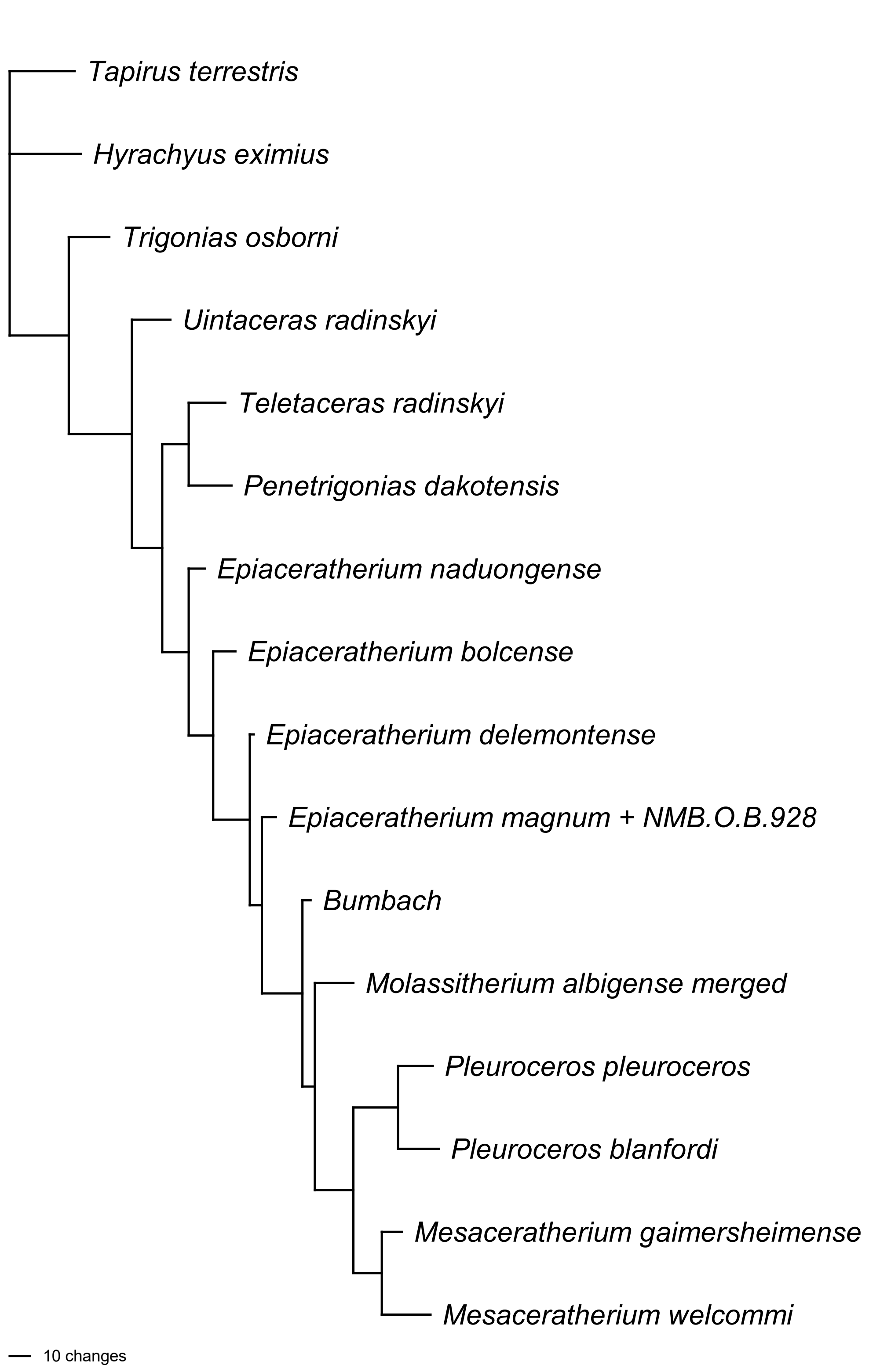
Single most parsimonious tree
Tapirus terrestris
Hyrachyus eximius
Trigonias osborni
Uintaceras radinskyi
Teletaceras radinskyi
Penetrigonias dakotensis
Epiaceratherium naduongense
Epiaceratherium bolcense
Epiaceratherium delemontense
Epiaceratherium magnum + NMB.O.B.928
Bumbach
Molassitherium albigense merged
Pleuroceros pleuroceros
Pleuroceros blanfordi
Mesaceratherium gaimersheimense
Mesaceratherium welcommi
10 changes

Single most parsimonious tree

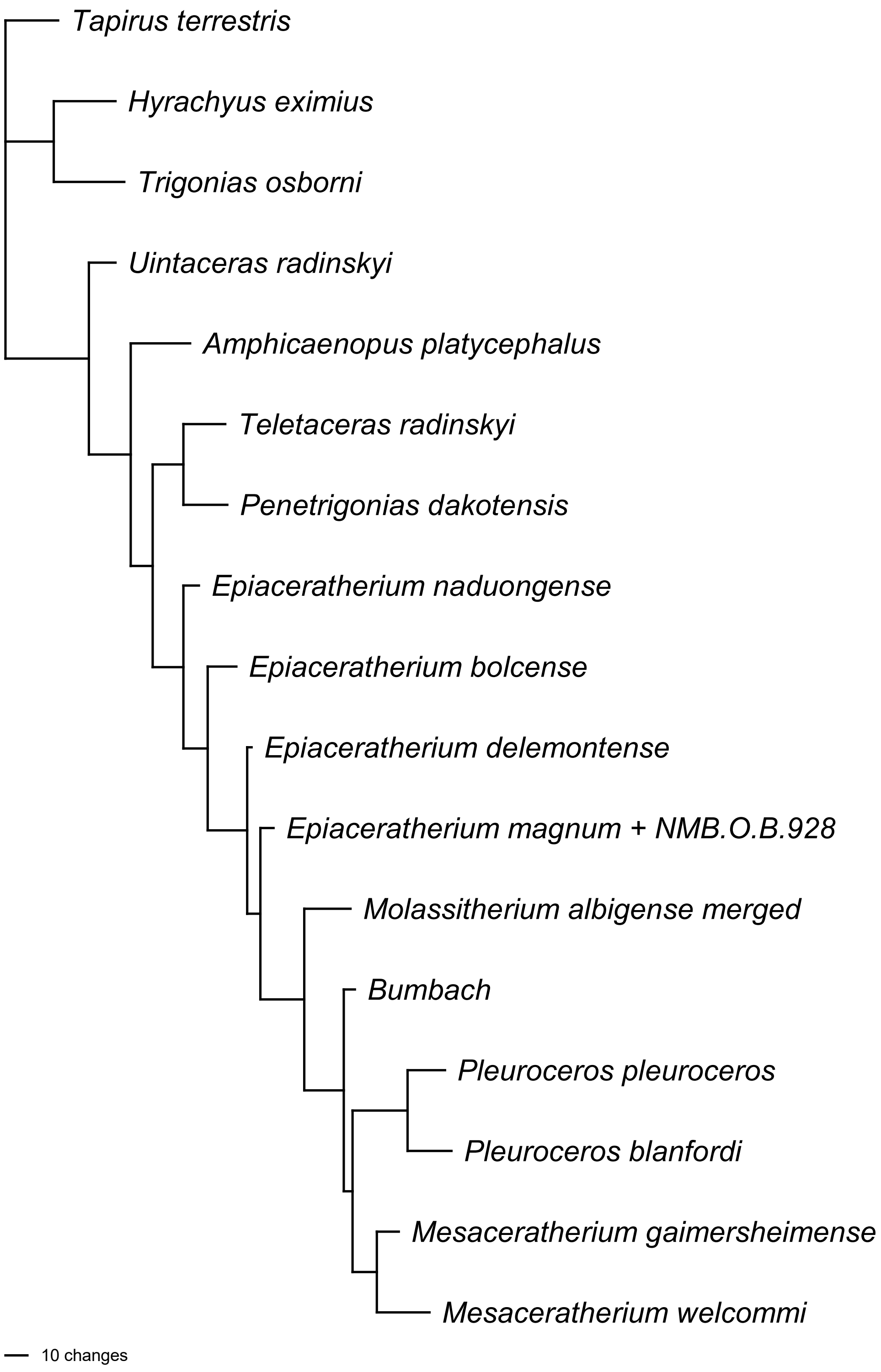

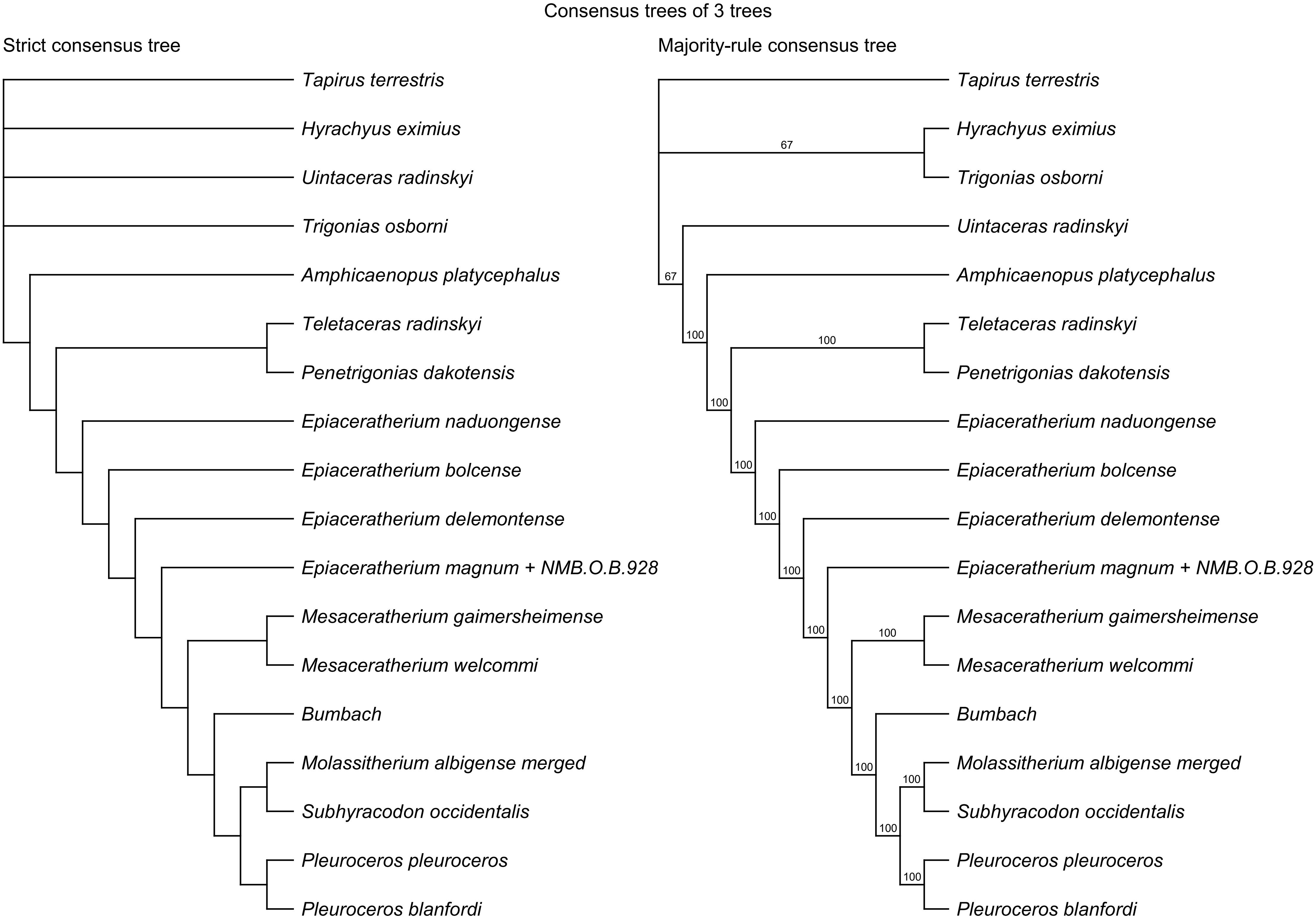

Consensus trees of 3 trees
Strict consensus tree
Tapirus terrestris
Hyrachyus eximius
Uintaceras radinskyi
Trigonias osborni
Amphicaenopus platycephalus
Teletaceras radinskyi
Penetrigonias dakotensis
Epiaceratherium naduongense
Epiaceratherium bolcense
Epiaceratherium delemontense
Epiaceratherium magnum + NMB.O.B.928
Mesaceratherium gaimersheimense
Mesaceratherium welcommi
Bumbach
Molassitherium albigense merged
Subhyracodon occidentalis
Pleuroceros pleuroceros
Pleuroceros blanfordi
Majority-rule consensus tree
Tapirus terrestris
67
Hyrachyus eximius
Trigonias osborni
Uintaceras radinskyi
67
Amphicaenopus platycephalus
100
100
Teletaceras radinskyi
Penetrigonias dakotensis
100
Epiaceratherium naduongense
100
Epiaceratherium bolcense
100
Epiaceratherium delemontense
100
Epiaceratherium magnum + NMB.O.B.928
100
100
Mesaceratherium gaimersheimense
Mesaceratherium welcommi
100
Bumbach
100
100
Molassitherium albigense merged
Subhyracodon occidentalis
100
100
Pleuroceros pleuroceros
Pleuroceros blanfordi

Single most parsimonious tree

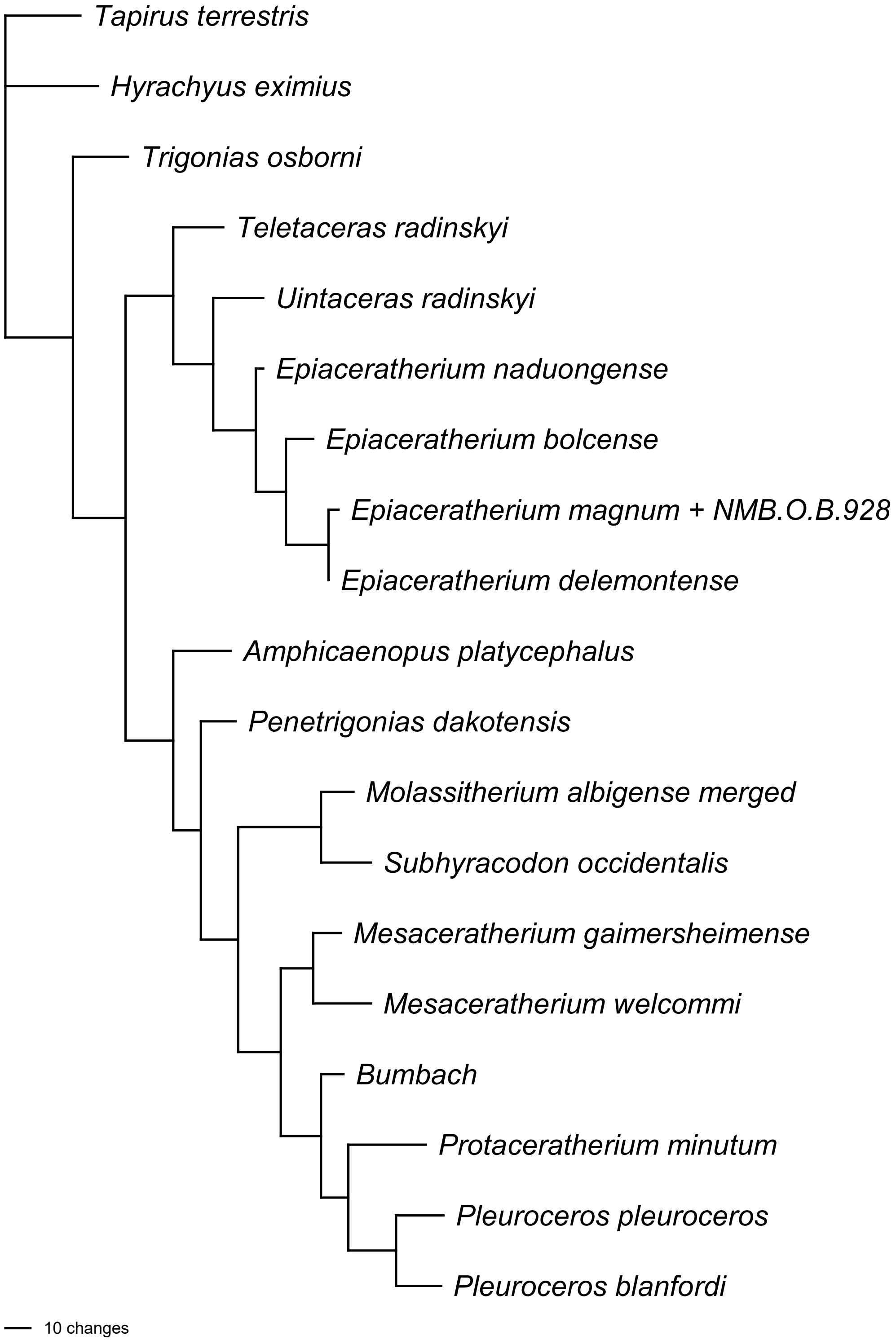

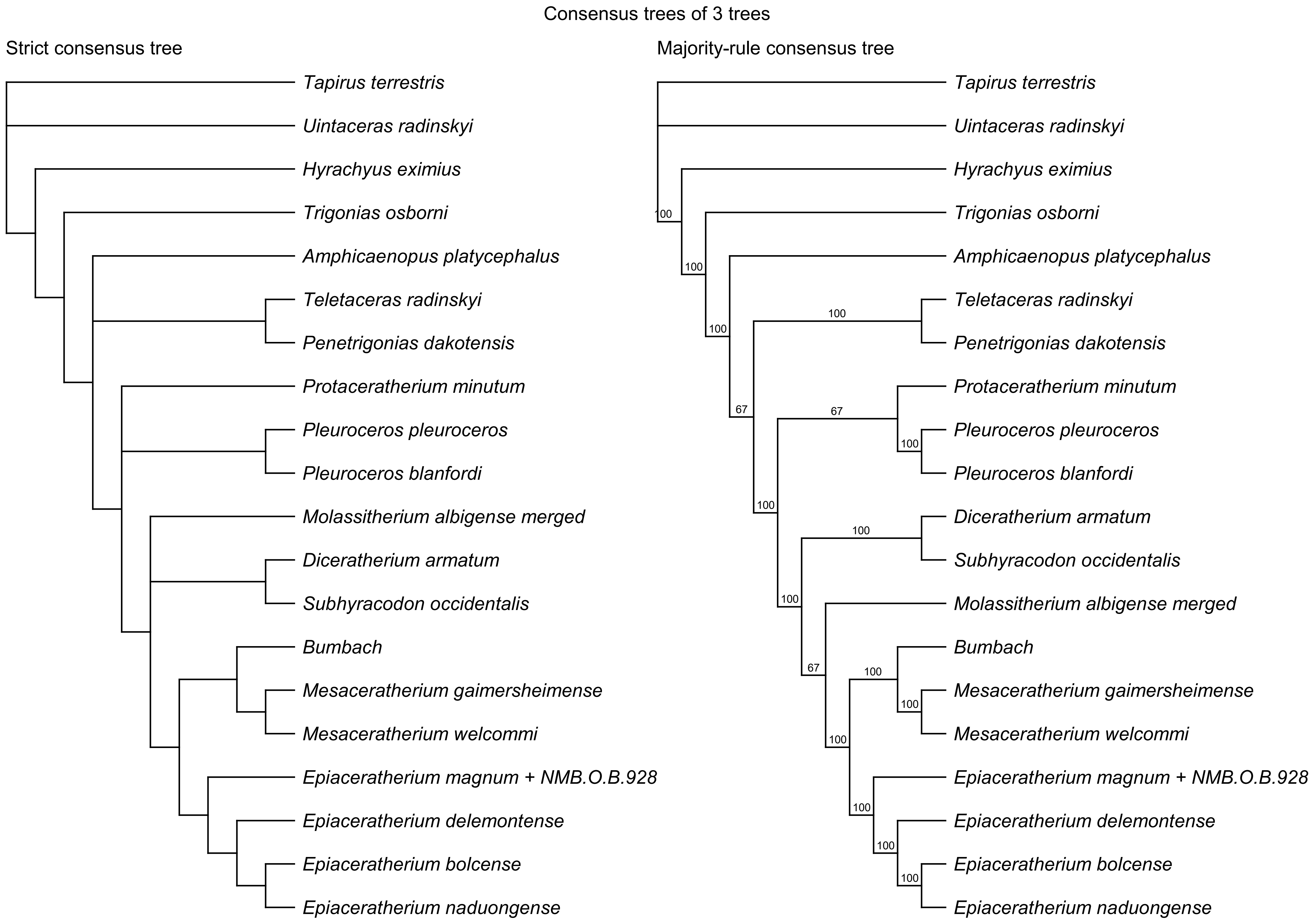
Consensus trees of 3 trees
Strict consensus tree
Tapirus terrestris
Uintaceras radinskyi
Hyrachyus eximius
Trigonias osborni
Amphicaenopus platycephalus
Teletaceras radinskyi
Penetrigonias dakotensis
Protaceratherium minutum
Pleuroceros pleuroceros
Pleuroceros blanfordi
Molassitherium albigense merged
Diceratherium armatum
Subhyracodon occidentalis
Bumbach
Mesaceratherium gaimersheimense
Mesaceratherium welcommi
Epiaceratherium magnum + NMB.O.B.928
Epiaceratherium delemontense
Epiaceratherium bolcense
Epiaceratherium naduongense
Majority-rule consensus tree
Tapirus terrestris
Uintaceras radinskyi
Hyrachyus eximius
Trigonias osborni
Amphicaenopus platycephalus
Teletaceras radinskyi
Penetrigonias dakotensis
Protaceratherium minutum
Pleuroceros pleuroceros
Pleuroceros blanfordi
Diceratherium armatum
Subhyracodon occidentalis
Molassitherium albigense merged
Bumbach
Mesaceratherium gaimersheimense
Mesaceratherium welcommi
Epiaceratherium magnum + NMB.O.B.928
Epiaceratherium delemontense
Epiaceratherium bolcense
Epiaceratherium naduongense
100
100
100
100
67
67
100
100
100
100
67
100
100
100
100
100
100

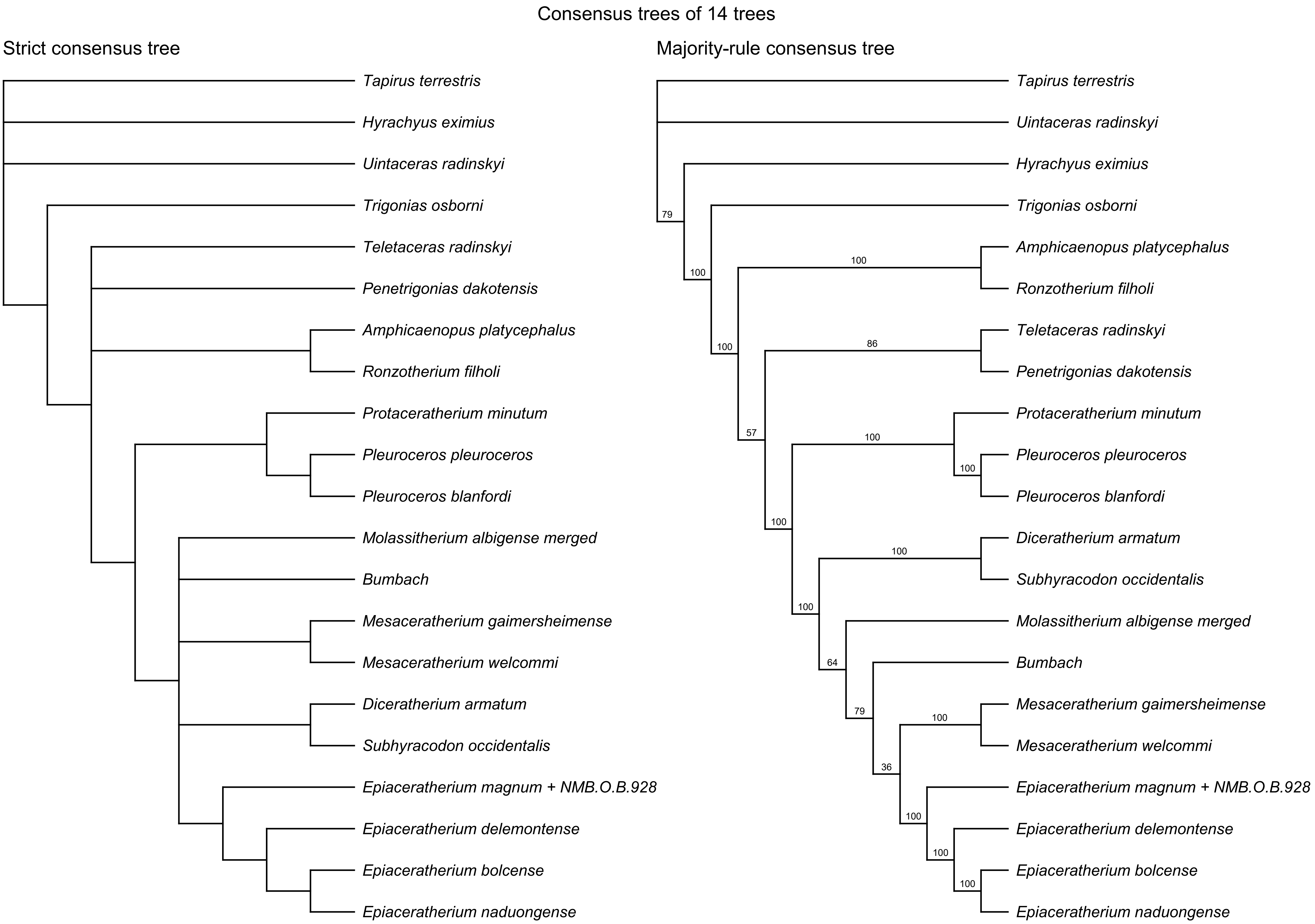
Consensus trees of 14 trees
Strict consensus tree
Tapirus terrestris
Hyrachyus eximius
Uintaceras radinskyi
Trigonias osborni
Teletaceras radinskyi
Penetrigonias dakotensis
Amphicaenopus platycephalus
Ronzotherium filholi
Protaceratherium minutum
Pleuroceros pleuroceros
Pleuroceros blanfordi
Molassitherium albigense merged
Bumbach
Mesaceratherium gaimersheimense
Mesaceratherium welcommi
Diceratherium armatum
Subhyracodon occidentalis
Epiaceratherium magnum + NMB.O.B.928
Epiaceratherium delemontense
Epiaceratherium bolcense
Epiaceratherium naduongense
Majority-rule consensus tree
Tapirus terrestris
Uintaceras radinskyi
Hyrachyus eximius
Trigonias osborni
Amphicaenopus platycephalus
Ronzotherium filholi
Teletaceras radinskyi
Penetrigonias dakotensis
Protaceratherium minutum
Pleuroceros pleuroceros
Pleuroceros blanfordi
Diceratherium armatum
Subhyracodon occidentalis
Molassitherium albigense merged
Bumbach
Mesaceratherium gaimersheimense
Mesaceratherium welcommi
Epiaceratherium magnum + NMB.O.B.928
Epiaceratherium delemontense
Epiaceratherium bolcense
Epiaceratherium naduongense
79
100
100
100
86
57
100
100
100
100
100
64
79
100
36
100
100
100

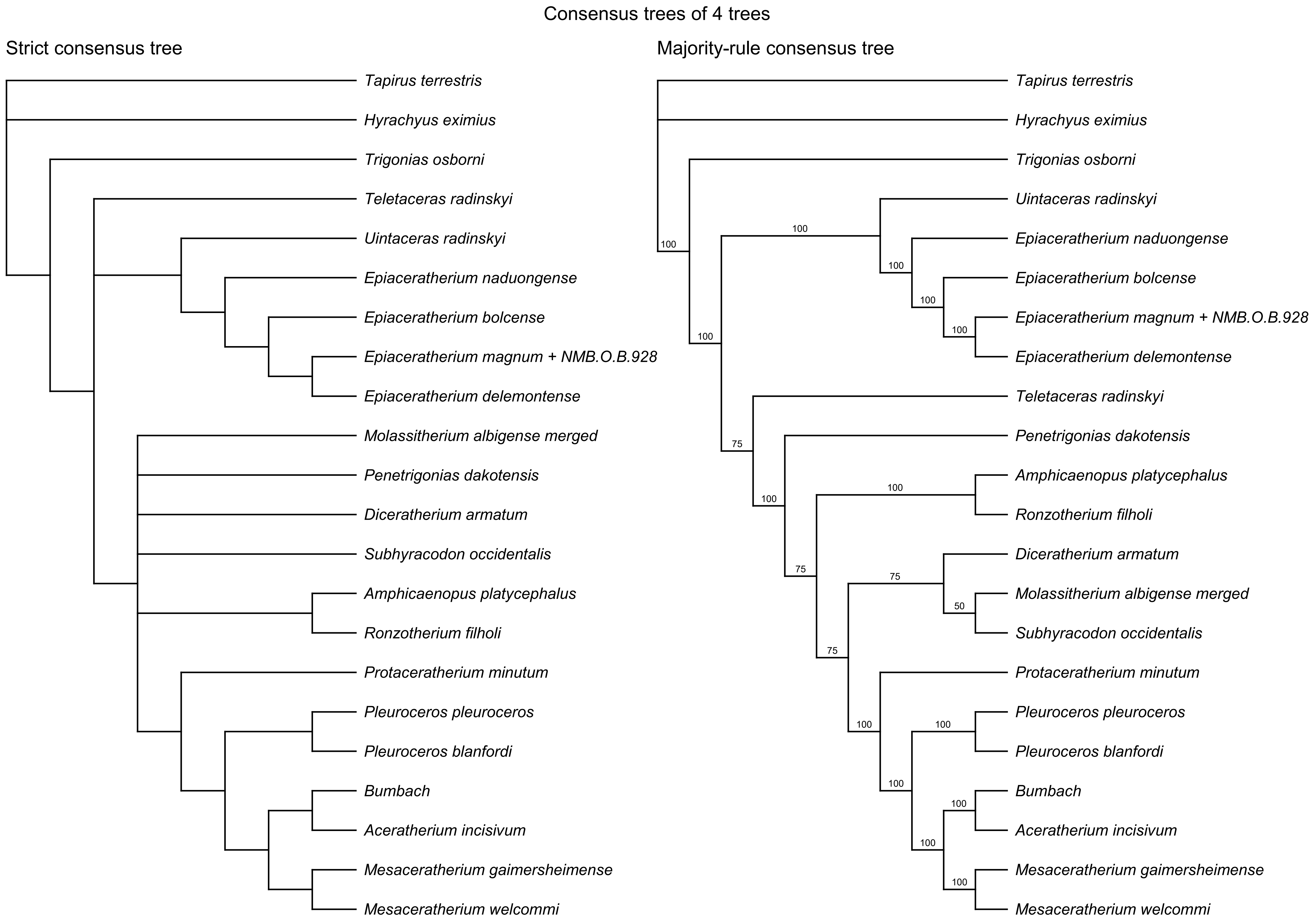

Consensus trees of 4 trees
Strict consensus tree
Tapirus terrestris
Hyrachyus eximius
Trigonias osborni
Teletaceras radinskyi
Uintaceras radinskyi
Epiaceratherium naduongense
Epiaceratherium bolcense
Epiaceratherium magnum + NMB.O.B.928
Epiaceratherium delemontense
Molassitherium albigense merged
Penetrigonias dakotensis
Diceratherium armatum
Subhyracodon occidentalis
Amphicaenopus platycephalus
Ronzotherium filholi
Protaceratherium minutum
Pleuroceros pleuroceros
Pleuroceros blanfordi
Bumbach
Aceratherium incisivum
Mesaceratherium gaimersheimense
Mesaceratherium welcommi
Majority-rule consensus tree
Tapirus terrestris
Hyrachyus eximius
Trigonias osborni
Uintaceras radinskyi
Epiaceratherium naduongense
Epiaceratherium bolcense
Epiaceratherium magnum + NMB.O.B.928
Epiaceratherium delemontense
Teletaceras radinskyi
Penetrigonias dakotensis
Amphicaenopus platycephalus
Ronzotherium filholi
Diceratherium armatum
Molassitherium albigense merged
Subhyracodon occidentalis
Protaceratherium minutum
Pleuroceros pleuroceros
Pleuroceros blanfordi
Bumbach
Aceratherium incisivum
Mesaceratherium gaimersheimense
Mesaceratherium welcommi
100
100
100
100
100
100
75
100
100
75
75
50
75
100
100
100
100
100
100

Consensus trees of 6 trees

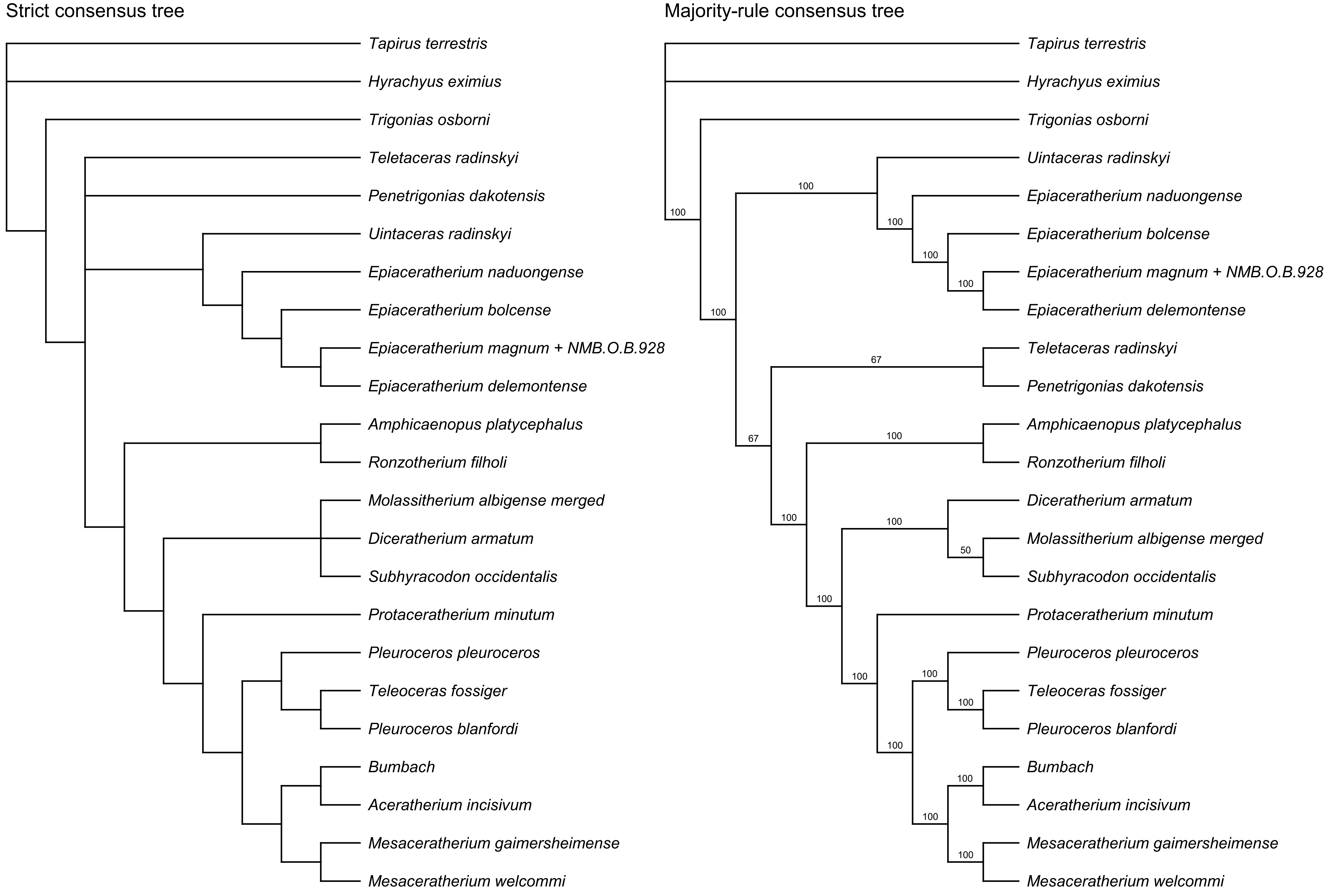

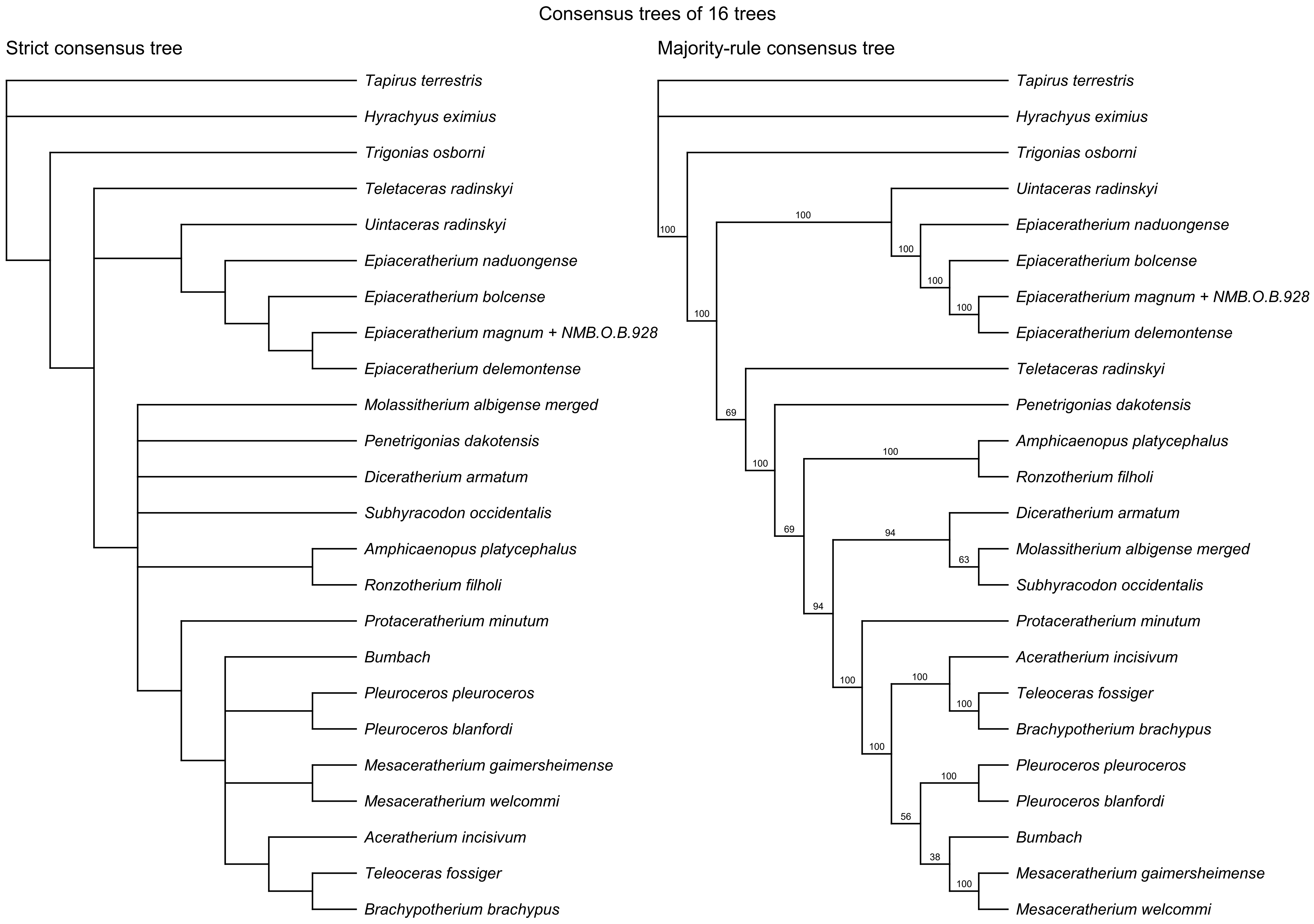

Consensus trees of 16 trees
Strict consensus tree
Majority-rule consensus tree
Tapirus terrestris
Hyrachyus eximius
Trigonias osborni
Teletaceras radinskyi
Uintaceras radinskyi
Epiaceratherium naduongense
Epiaceratherium bolcense
Epiaceratherium magnum + NMB.O.B.928
Epiaceratherium delemontense
Molassitherium albigense merged
Penetrigonias dakotensis
Diceratherium armatum
Subhyracodon occidentalis
Amphicaenopus platycephalus
Ronzotherium filholi
Protaceratherium minutum
Bumbach
Pleuroceros pleuroceros
Pleuroceros blanfordi
Mesaceratherium gaimersheimense
Mesaceratherium welcommi
Aceratherium incisivum
Teleoceras fossiger
Brachypotherium brachypus
100
69
94
63
56
38

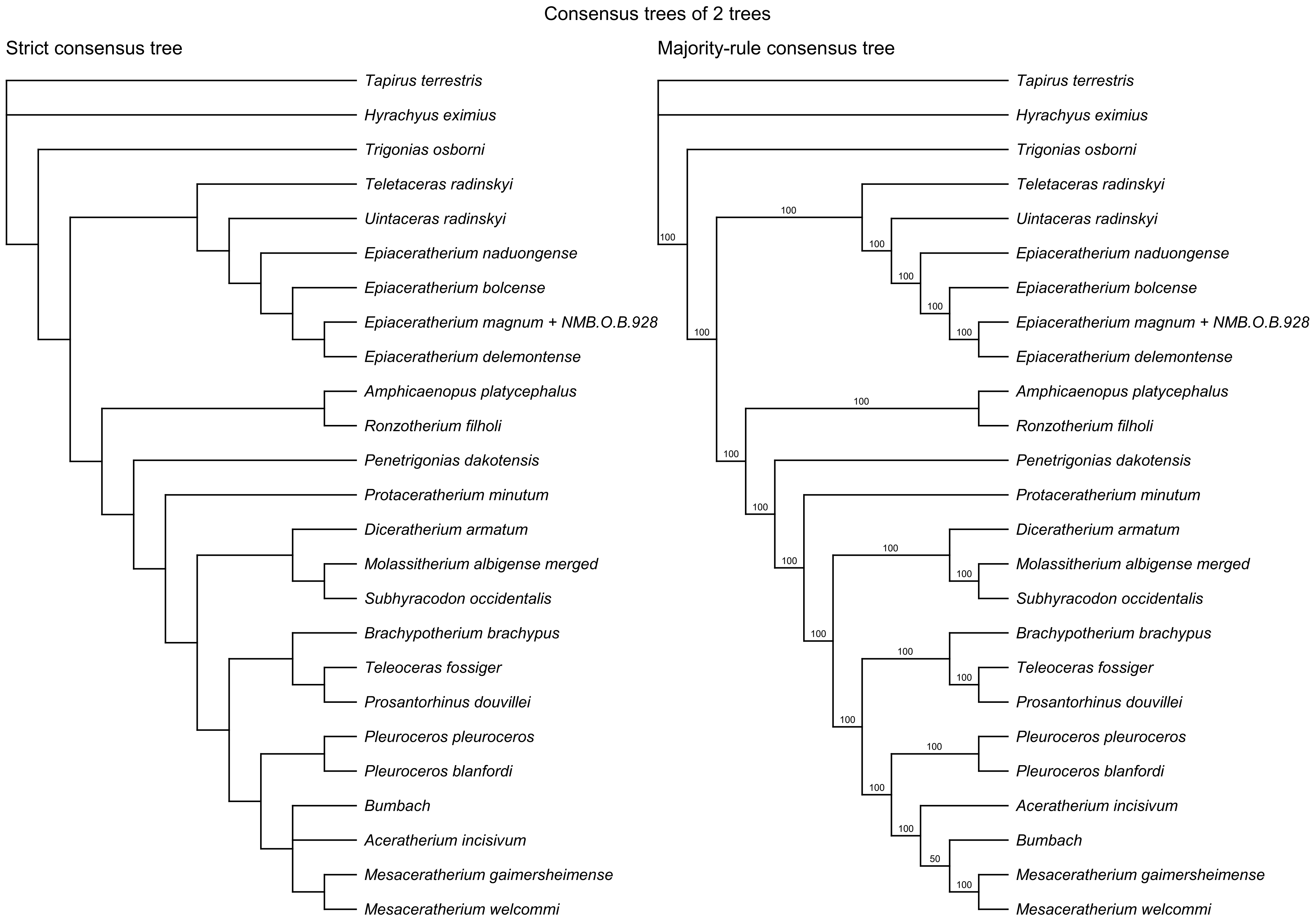
Consensus trees of 2 trees
Strict consensus tree
Majority-rule consensus tree
Tapirus terrestris
Hyrachyus eximius
Trigonias osborni
Teletaceras radinskyi
Uintaceras radinskyi
Epiaceratherium naduongense
Epiaceratherium bolcense
Epiaceratherium magnum + NMB.O.B.928
Epiaceratherium delemontense
Amphicaenopus platycephalus
Ronzotherium filholi
Penetrigonias dakotensis
Protaceratherium minutum
Diceratherium armatum
Molassitherium albigense merged
Subhyracodon occidentalis
Brachypotherium brachypus
Teleoceras fossiger
Prosantorhinus douvillei
Pleuroceros pleuroceros
Pleuroceros blanfordi
Bumbach
Aceratherium incisivum
Mesaceratherium gaimersheimense
Mesaceratherium welcommi
100
50

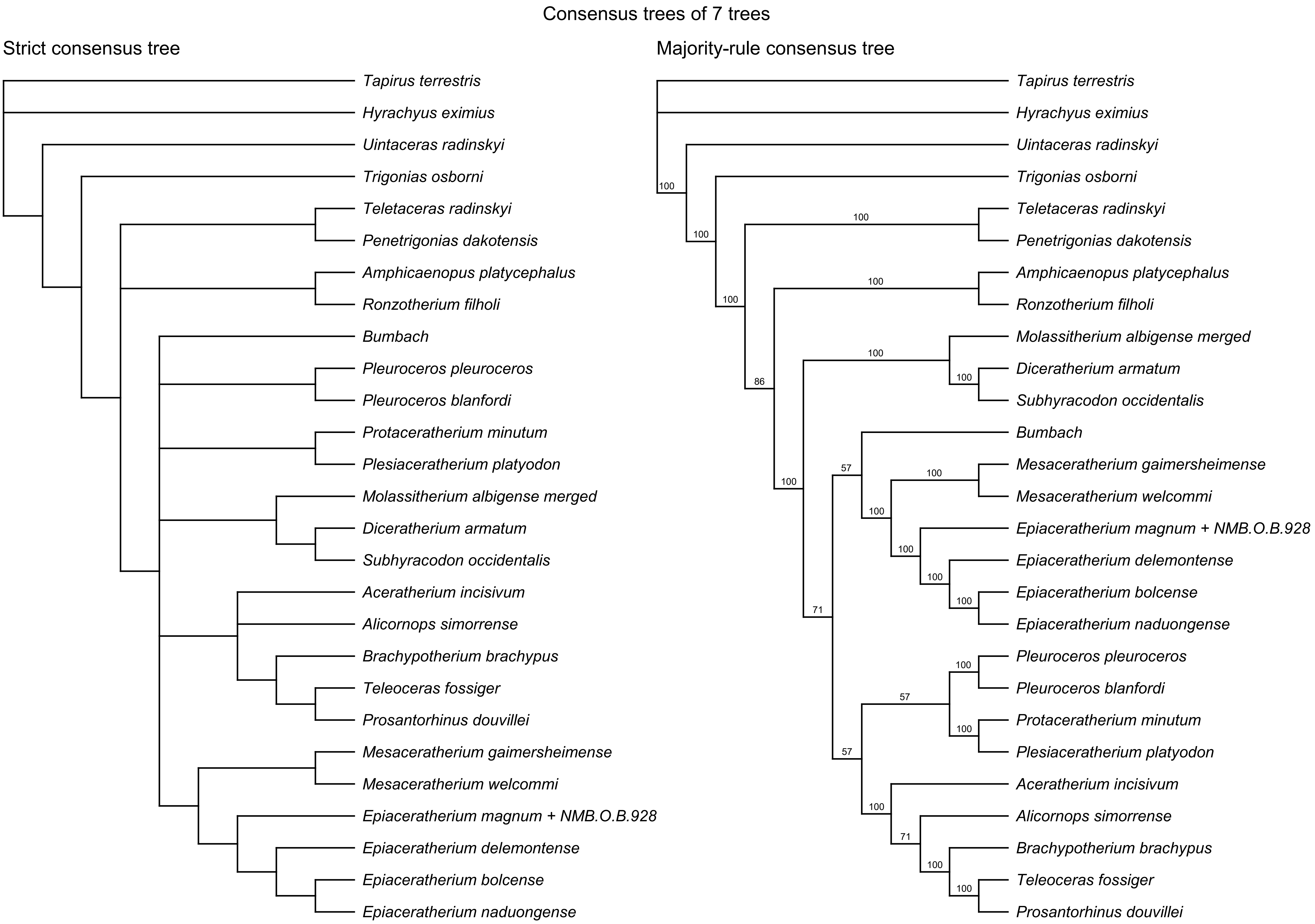
Consensus trees of 7 trees
Strict consensus tree
Tapirus terrestris
Hyrachyus eximius
Uintaceras radinskyi
Trigonias osborni
Teletaceras radinskyi
Penetrigonias dakotensis
Amphicaenopus platycephalus
Ronzotherium filholi
Bumbach
Pleuroceros pleuroceros
Pleuroceros blanfordi
Protaceratherium minutum
Plesiaceratherium platyodon
Molassitherium albigense merged
Diceratherium armatum
Subhyracodon occidentalis
Aceratherium incisivum
Alicornops simorrense
Brachypotherium brachypus
Teleoceras fossiger
Prosantorhinus douvillei
Mesaceratherium gaimersheimense
Mesaceratherium welcommi
Epiaceratherium magnum + NMB.O.B.928
Epiaceratherium delemontense
Epiaceratherium bolcense
Epiaceratherium naduongense
Majority-rule consensus tree
Tapirus terrestris
Hyrachyus eximius
Uintaceras radinskyi
Trigonias osborni
Teletaceras radinskyi
Penetrigonias dakotensis
Amphicaenopus platycephalus
Ronzotherium filholi
Molassitherium albigense merged
Diceratherium armatum
Subhyracodon occidentalis
Bumbach
Mesaceratherium gaimersheimense
Mesaceratherium welcommi
Epiaceratherium magnum + NMB.O.B.928
Epiaceratherium delemontense
Epiaceratherium bolcense
Epiaceratherium naduongense
Pleuroceros pleuroceros
Pleuroceros blanfordi
Protaceratherium minutum
Plesiaceratherium platyodon
Aceratherium incisivum
Alicornops simorrense
Brachypotherium brachypus
Teleoceras fossiger
Prosantorhinus douvillei
100
100
100
100
100
86
100
100
100
57
100
100
100
100
100
71
100
57
100
57
100
71
100
100

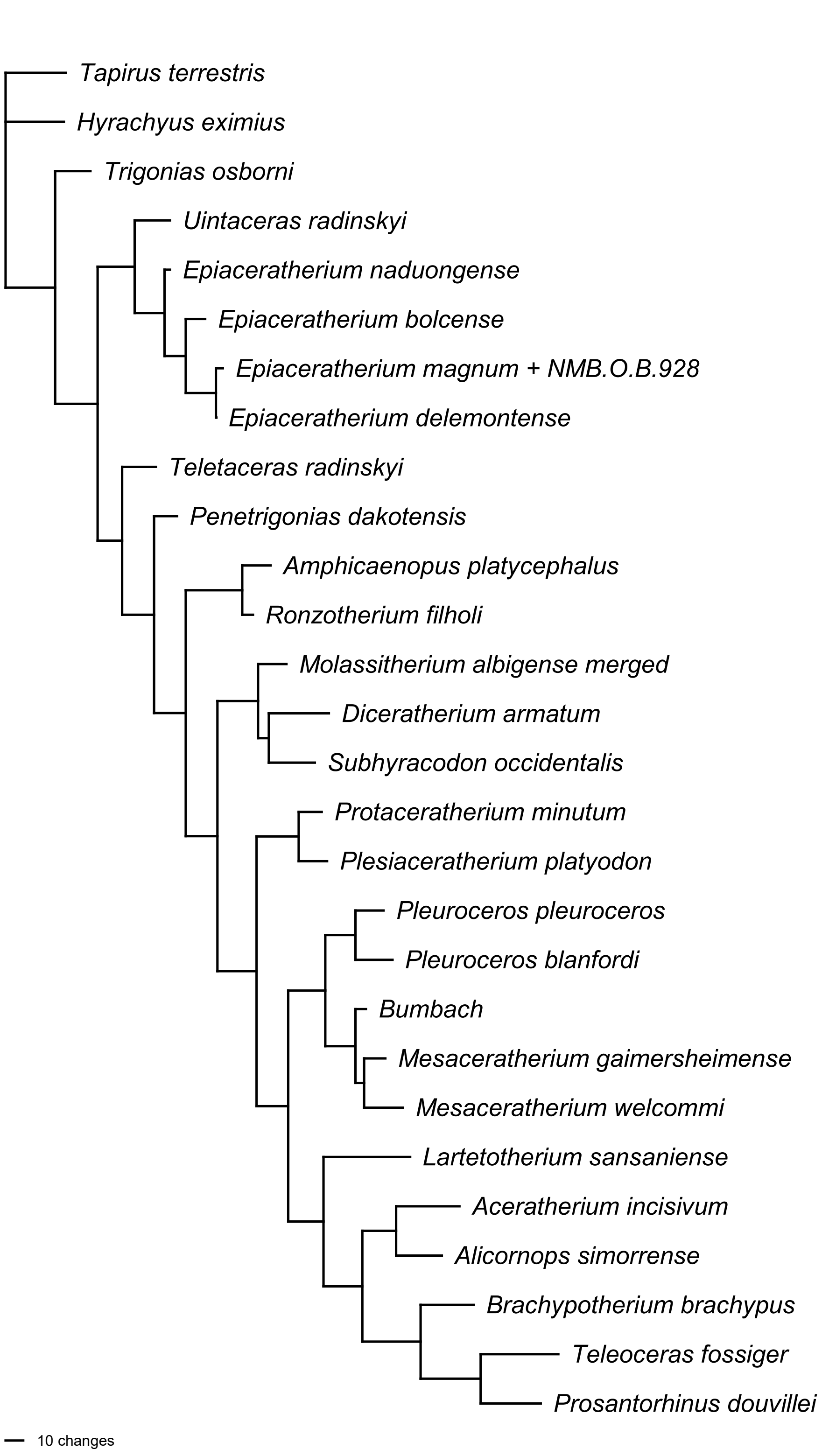
Single most parsimonious tree
Tapirus terrestris
Hyrachyus eximius
Trigonias osborni
Uintaceras radinskyi
Epiaceratherium naduongense
Epiaceratherium bolcense
Epiaceratherium magnum + NMB.O.B.928
Epiaceratherium delemontense
Teletaceras radinskyi
Penetrigonias dakotensis
Amphicaenopus platycephalus
Ronzotherium filholi
Molassitherium albigense merged
Diceratherium armatum
Subhyracodon occidentalis
Protaceratherium minutum
Plesiaceratherium platyodon
Pleuroceros pleuroceros
Pleuroceros blanfordi
Bumbach
Mesaceratherium gaimersheimense
Mesaceratherium welcommi
Lartetotherium sansaniense
Aceratherium incisivum
Alicornops simorrense
Brachypotherium brachypus
Teleoceras fossiger
Prosantorhinus douvillei
10 changes

Single most-parsimonious tree - final

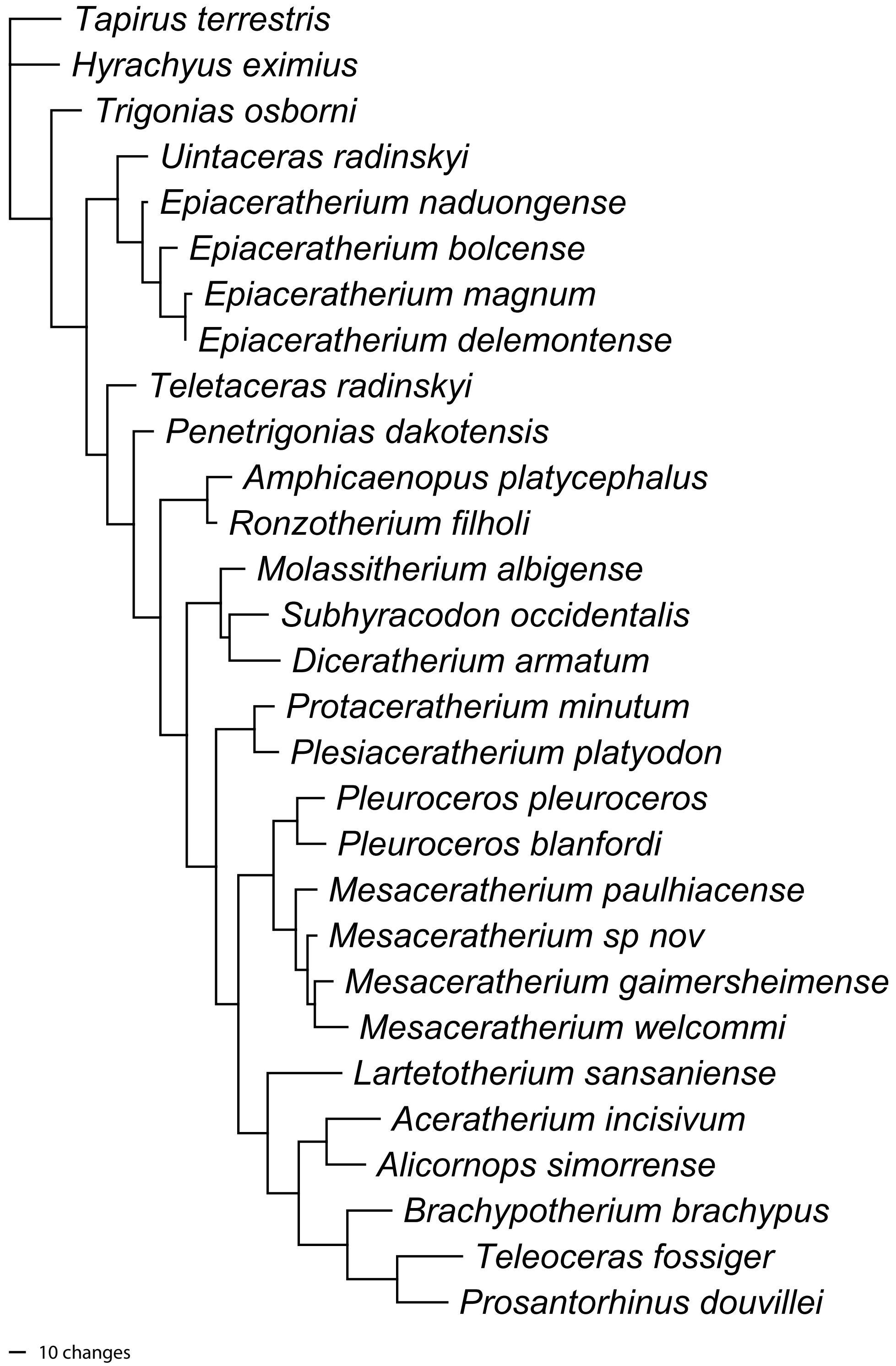

Supplement: Additional trees [file rsos200633supp2.pdf]
