# Supplementary material for: New material of Epiaceratherium and a new species of Mesaceratherium clear up the phylogeny of early Rhinocerotidae (Perissodactyla)
Source: R Soc Open Sci. 2020 Jul 15;7(7):200633. doi: 10.1098/rsos.200633 (PMC7428265; doi:10.1098/rsos.200633)
Supplement: Reviewer comments [file rsos200633_review_history.pdf]

THE ROYAL SOCIETY
PUBLISHING

ROYAL SOCIETY
OPEN SCIENCE

New material of *Epiaceratherium* and a new species of *Mesaceratherium* clear up the phylogeny of early rhinocerotidae (perissodactyla)

Jérémy Tissier, Pierre-Olivier Antoine and Damien Becker

Article citation details
R. Soc. open sci. **7**: 200633.
http://dx.doi.org/10.1098/rsos.200633

Review timeline
Original submission: 16 April 2020
Revised submission: 8 June 2020
Final acceptance: 10 June 2020

Note: Reports are unedited and appear as submitted by the referee. The review history appears in chronological order.

Review History

RSOS-200633.R0 (Original submission)

Review form: Reviewer 1 (Naoto Handa)

Is the manuscript scientifically sound in its present form?
Yes

Are the interpretations and conclusions justified by the results?
Yes

Is the language acceptable?
Yes

Do you have any ethical concerns with this paper?
No

Have you any concerns about statistical analyses in this paper?
No

Recommendation?

Reports © 2020 The Reviewers; Decision Letters © 2020 The Reviewers and Editors; Responses © 2020 The Reviewers, Editors and Authors. Published by the Royal Society under the terms of the Creative Commons Attribution License http://creativecommons.org/licenses/by/4.0/, which permits unrestricted use, provided the original author and source are credited

Accept with minor revision (please list in comments)

Comments to the Author(s)
This paper shows very good result for main part of description and comparisons.
I have found a few issues that, once addressed, will improve the manuscript.

- This paper shows various abbreviations for measurements of postcranials in tables 3, 4 and p. 14, i. 34. therefore, please add to the explanations of those abbreviations in full in Terminologyy section.

- This paper discusses the reduction of the anterior dention among rhinocerotids. If it possible, I suggest to add a picture of dorsal view of the symphysis part of the Mesaceratherium tschani (NMBE-D1836/1837) in Fig. 2 because it would be difficult for some scholars to understand the detailed morphology of the part.

- In this paper, various articular facets of the postcranials of Mesaceratherium tschani are triated as key characters in the diagnosis and comparisons sections. Therefore, I suggest to add nomenclatures of those facets in Fig. 3.

Review form: Reviewer 2 (Luca Pandolfi)

Is the manuscript scientifically sound in its present form?
Yes

Are the interpretations and conclusions justified by the results?
Yes

Is the language acceptable?
Yes

Do you have any ethical concerns with this paper?
No

Have you any concerns about statistical analyses in this paper?
No

Recommendation?
Accept with minor revision (please list in comments)

Comments to the Author(s)
The manuscript deals with the description of new rhinocerotids remains from the Oligocene of Europe and their significative contribution in the understanding of the evolution of early Rhinocerotidae. The manuscript is well-written (but I' m not a native speaker); the descriptions are accompained by an exhaustive phylogenetic analysis and the data are available for the readers that will be enable to reproduce the analyses. The data reported in the manuscript will be very useful for future papers on early rhinocerotids. The conclusions are well-supported by data. I added only a few comments in the attached files (Appendix A). The main comment is regarding the specimen NMBIO43; I added a comment with infos that the authors could be added in the text if they want. Minor comments are for tables and a figure and concerning lower teeth of Molassitherium delemontense. Anyway, these comments do not invalidate results, conclusions or analyses and I strongly recommend the publication of this manuscript.

Decision letter (RSOS-200633.R0)

We hope you are keeping well at this difficult and unusual time. We continue to value your support of the journal in these challenging circumstances. If Royal Society Open Science can assist you at all, please don't hesitate to let us know at the email address below.

Dear Mr Tissier,

On behalf of the Editors, I am pleased to inform you that your Manuscript RSOS-200633 entitled "New material of *Epiaceratherium* and a new species of *Mesaceratherium* clear up the phylogeny of the early Rhinocerotidae (Perissodactyla)" has been accepted for publication in Royal Society Open Science subject to minor revision in accordance with the referee suggestions. Please find the referees' comments at the end of this email.

The reviewers and handling editors have recommended publication, but also suggest some minor revisions to your manuscript. Therefore, I invite you to respond to the comments and revise your manuscript.

• Ethics statement
If your study uses humans or animals please include details of the ethical approval received, including the name of the committee that granted approval. For human studies please also detail whether informed consent was obtained. For field studies on animals please include details of all permissions, licences and/or approvals granted to carry out the fieldwork.

• Data accessibility
It is a condition of publication that all supporting data are made available either as supplementary information or preferably in a suitable permanent repository. The data accessibility section should state where the article's supporting data can be accessed. This section should also include details, where possible of where to access other relevant research materials such as statistical tools, protocols, software etc can be accessed. If the data has been deposited in an external repository this section should list the database, accession number and link to the DOI for all data from the article that has been made publicly available. Data sets that have been deposited in an external repository and have a DOI should also be appropriately cited in the manuscript and included in the reference list.

If you wish to submit your supporting data or code to Dryad (http://datadryad.org/), or modify your current submission to dryad, please use the following link:
http://datadryad.org/submit?journalID=RSOS&manu=RSOS-200633

• Competing interests
Please declare any financial or non-financial competing interests, or state that you have no competing interests.
• Authors' contributions
All submissions, other than those with a single author, must include an Authors' Contributions section which individually lists the specific contribution of each author. The list of Authors should meet all of the following criteria; 1) substantial contributions to conception and design, or acquisition of data, or analysis and interpretation of data; 2) drafting the article or revising it critically for important intellectual content; and 3) final approval of the version to be published.

All contributors who do not meet all of these criteria should be included in the acknowledgements.

We suggest the following format:

AB carried out the molecular lab work, participated in data analysis, carried out sequence alignments, participated in the design of the study and drafted the manuscript; CD carried out the statistical analyses; EF collected field data; GH conceived of the study, designed the study, coordinated the study and helped draft the manuscript. All authors gave final approval for publication.

• Acknowledgements
Please acknowledge anyone who contributed to the study but did not meet the authorship criteria.

• Funding statement
Please list the source of funding for each author.

Please ensure you have prepared your revision in accordance with the guidance at https://royalsociety.org/journals/authors/author-guidelines/ -- please note that we cannot publish your manuscript without the end statements. We have included a screenshot example of the end statements for reference. If you feel that a given heading is not relevant to your paper, please nevertheless include the heading and explicitly state that it is not relevant to your work.

Because the schedule for publication is very tight, it is a condition of publication that you submit the revised version of your manuscript before 05-Jun-2020. Please note that the revision deadline will expire at 00.00am on this date. If you do not think you will be able to meet this date please let me know immediately.

To revise your manuscript, log into https://mc.manuscriptcentral.com/rsos and enter your Author Centre, where you will find your manuscript title listed under "Manuscripts with Decisions". Under "Actions," click on "Create a Revision." You will be unable to make your revisions on the originally submitted version of the manuscript. Instead, revise your manuscript and upload a new version through your Author Centre.

When submitting your revised manuscript, you will be able to respond to the comments made by the referees and upload a file "Response to Referees" in "Section 6 - File Upload". You can use this to document any changes you make to the original manuscript. In order to expedite the processing of the revised manuscript, please be as specific as possible in your response to the referees. We strongly recommend uploading two versions of your revised manuscript:

1) Identifying all the changes that have been made (for instance, in coloured highlight, in bold text, or tracked changes);
2) A 'clean' version of the new manuscript that incorporates the changes made, but does not highlight them.

When uploading your revised files please make sure that you have:

1) A text file of the manuscript (tex, txt, rtf, docx or doc), references, tables (including captions) and figure captions. Do not upload a PDF as your "Main Document";
2) A separate electronic file of each figure (EPS or print-quality PDF preferred (either format should be produced directly from original creation package), or original software format);
3) Included a 100 word media summary of your paper when requested at submission. Please ensure you have entered correct contact details (email, institution and telephone) in your user account;
4) Included the raw data to support the claims made in your paper. You can either include your data as electronic supplementary material or upload to a repository and include the relevant doi within your manuscript. Make sure it is clear in your data accessibility statement how the data can be accessed;
5) All supplementary materials accompanying an accepted article will be treated as in their final form. Note that the Royal Society will neither edit nor typeset supplementary material and it will

be hosted as provided. Please ensure that the supplementary material includes the paper details where possible (authors, article title, journal name).

Supplementary files will be published alongside the paper on the journal website and posted on the online figshare repository (https://rs.figshare.com/). The heading and legend provided for each supplementary file during the submission process will be used to create the figshare page, so please ensure these are accurate and informative so that your files can be found in searches. Files on figshare will be made available approximately one week before the accompanying article so that the supplementary material can be attributed a unique DOI.

Please note that Royal Society Open Science charge article processing charges for all new submissions that are accepted for publication. Charges will also apply to papers transferred to Royal Society Open Science from other Royal Society Publishing journals, as well as papers submitted as part of our collaboration with the Royal Society of Chemistry (https://royalsocietypublishing.org/rsos/chemistry).

If your manuscript is newly submitted and subsequently accepted for publication, you will be asked to pay the article processing charge, unless you request a waiver and this is approved by Royal Society Publishing. You can find out more about the charges at https://royalsocietypublishing.org/rsos/charges. Should you have any queries, please contact openscience@royalsociety.org.

Once again, thank you for submitting your manuscript to Royal Society Open Science and I look forward to receiving your revision. If you have any questions at all, please do not hesitate to get in touch.

Best regards,

Lianne Parkhouse
Editorial Coordinator
Royal Society Open Science
openscience@royalsociety.org

on behalf of Professor Allison Daley (Associate Editor) and Kevin Padian (Subject Editor)
openscience@royalsociety.org

Associate Editor Comments to Author (Professor Allison Daley):

Both reviewers gave very positive assessments of the manuscript, commenting on the high quality of the descriptions and phylogenetic analysis. Please incorporate the reviewers requests to add additional images to Figures 2 and 3, and modifications to the data Tables 3 and 4. Please also add text to the manuscript addressing the comment about specimen NMB I.O.43, that is a hypothetical reconstruction of the snout (in gypsum) and it is not an original specimen. In addition, make the minor changes suggested in the annotated PDF files.

Reviewer comments to Author:

Reviewer: 1
Comments to the Author(s)

This paper shows very good result for main part of description and comparisons.
I have found a few issues that, once addressed, will improve the manuscript.

- This paper shows various abbreviations for measurements of postcranials in tables 3, 4 and p. 14, i. 34. therefore, please add to the explanations of those abbreviations in full in Terminologyy section.

- This paper discusses the reduction of the anterior dention among rhinocerotids. If it possible, I suggest to add a picture of dorsal view of the symphysis part of the Mesaceratherium tschani (NMBE-D1836/1837) in Fig. 2 because it would be difficult for some scholars to understand the detailed morphology of the part.

- In this paper, various articular facets of the postcranials of Mesaceratherium tschani are triated as key characters in the diagnosis and comparisons sections. Therefore, I suggest to add nomenclatures of those facets in Fig. 3.

Reviewer: 2
Comments to the Author(s)

The manuscript deals with the description of new rhinocerotids remains from the Oligocene of Europe and their significative contribution in the understanding of the evolution of early Rhinocerotidae. The manuscript is well-written (but I′ m not a native speaker); the descriptions are accompained by an exhaustive phylogenetic analysis and the data are available for the readers that will be enable to reproduce the analyses. The data reported in the manuscript will be very useful for future papers on early rhinocerotids. The conclusions are well-supported by data. I added only a few comments in the attached files. The main comment is regarding the specimen NMBIO43; I added a comment with infos that the authors could be added in the text if they want. Minor comments are for tables and a figure and concerning lower teeth of Molassitherium delemontense. Anyway, these comments do not invalidate results, conclusions or analyses and I strongly recommend the publication of this manuscript.

Author's Response to Decision Letter for (RSOS-200633.R0)

See Appendix B.

Decision letter (RSOS-200633.R1)

We hope you are keeping well at this difficult and unusual time. We continue to value your support of the journal in these challenging circumstances. If Royal Society Open Science can assist you at all, please don't hesitate to let us know at the email address below.

Dear Mr Tissier,

It is a pleasure to accept your manuscript entitled "New material of Epiaceratherium and a new species of Mesaceratherium clear up the phylogeny of the early Rhinocerotidae (Perissodactyla)" in its current form for publication in Royal Society Open Science.

Please ensure that you send to the editorial office an editable version of your accepted manuscript, and individual files for each figure and table included in your manuscript. You can send these in a zip folder if more convenient. Failure to provide these files may delay the

processing of your proof. You may disregard this request if you have already provided these files to the editorial office.

You can expect to receive a proof of your article in the near future. Please contact the editorial office (openscience_proofs@royalsociety.org) and the production office (openscience@royalsociety.org) to let us know if you are likely to be away from e-mail contact -- if you are going to be away, please nominate a co-author (if available) to manage the proofing process, and ensure they are copied into your email to the journal.

Due to rapid publication and an extremely tight schedule, if comments are not received, your paper may experience a delay in publication. Royal Society Open Science operates under a continuous publication model. Your article will be published straight into the next open issue and this will be the final version of the paper. As such, it can be cited immediately by other researchers. As the issue version of your paper will be the only version to be published I would advise you to check your proofs thoroughly as changes cannot be made once the paper is published.

Please see the Royal Society Publishing guidance on how you may share your accepted author manuscript at https://royalsociety.org/journals/ethics-policies/media-embargo/.

Thank you for your fine contribution. On behalf of the Editors of Royal Society Open Science, we look forward to your continued contributions to the Journal.

Kind regards,
Royal Society Open Science Editorial Office
Royal Society Open Science
openscience@royalsociety.org

on behalf of Professor Allison Daley (Associate Editor) and Kevin Padian (Subject Editor)
openscience@royalsociety.org

Follow Royal Society Publishing on Twitter: @RSocPublishing
Follow Royal Society Publishing on Facebook:
https://www.facebook.com/RoyalSocietyPublishing.FanPage/
Read Royal Society Publishing's blog: https://blogs.royalsociety.org/publishing/

Appendix A

ROYAL SOCIETY
OPEN SCIENCE

New material of *Epiaceratherium* and a new species of *Mesaceratherium* clear up the phylogeny of the early Rhinocerotidae (Perissodactyla)

Journal:	*Royal Society Open Science*
Manuscript ID	RSOS-200633
Article Type:	Research
Date Submitted by the Author:	16-Apr-2020
Complete List of Authors:	Tissier, Jérémy; JURASSICA Museum; Universite de Fribourg, Département des Géosciences<br>ANTOINE, Pierre-Olivier; Institut des Sciences de l'Evolution de Montpellier (ISE-M), UMR 5554 CNRS/UM/IRD/EPHE, Paleontology<br>Becker, Damien; JURASSICA Museum; Universite de Fribourg, Département des Géosciences
Subject:	palaeontology < BIOLOGY, Palaeontology < EARTH SCIENCES, evolution < BIOLOGY
Keywords:	Epiaceratherium, Mesaceratherium, New species, Rhinocerotidae, Oligocene, Phylogeny
Subject Category:	Organismal and Evolutionary Biology

SCHOLARONE™
Manuscripts

Author-supplied statements

Relevant information will appear here if provided.

Ethics

Does your article include research that required ethical approval or permits?:
This article does not present research with ethical considerations

Statement (if applicable):
CUST_IF_YES_ETHICS :No data available.

Data

It is a condition of publication that data, code and materials supporting your paper are made publicly available. Does your paper present new data?:
Yes

Statement (if applicable):
All data generated or analysed during this study are included in this published article (and its supplementary information files). The morphological data matrix is available in NEXUS format in Supplementary Material 1. 3D models will be available from the MorphoMuseuM repository with DOIs and are temporarily accessible at these urls: http://morphomuseum.com/Specimenfiles/sendFile/534/762cc9 (3D surface model of the mandible NMB.O.B.928) and http://morphomuseum.com/Specimenfiles/sendFile/535/f4de1d (3D surface model of the archetype reconstruction of Epiaceratherium).

Conflict of interest

I/We declare we have no competing interests

Statement (if applicable):
CUST_STATE_CONFLICT :No data available.

Authors' contributions

This paper has multiple authors and our individual contributions were as below

Statement (if applicable):
All authors contributed to the study conception and design. Material study and analysis were performed by all authors. The first draft of the manuscript was written by Damien Becker and JÃ©rÃ©my Tissier. All authors gave final approval for publication and agree to be held accountable for the work performed therein.

New material of *Epiaceratherium* and a new species of *Mesaceratherium* clear up the phylogeny of early Rhinocerotidae (Perissodactyla)

Jérémy Tissier[1, 2, *], Pierre-Olivier Antoine[3] & Damien Becker[1, 2]

[1] JURASSICA Museum, Porrentruy, Jura Canton, Switzerland

[2] Université de Fribourg, Department of Geosciences, Fribourg, Fribourg Canton, Switzerland

[3] Université de Montpellier, Institut des sciences de l'évolution, Montpellier, France

[*] Corresponding author: jeremy.tissier@unifr.ch

ORCID:

- Jérémy Tissier: 0000-0002-8517-1612
- Pierre-Olivier Antoine: 0000-0001-9122-1818
- Damien Becker: 0000-0002-2226-9946

Abstract

Reduction of the anterior dentition (i.e. incisors and canines) is a major adaptative trait of the Rhinocerotidae among Perissodactyla. However, the corresponding evolutionary sequence was lacking a robust phylogenetic frame to support it thus far. Here, we describe a new Oligocene species of Rhinocerotinae, *Mesaceratherium* sp. nov. from the Swiss locality of Bumbach (MP25 reference level). In addition, we identify the only known complete mandible of *Epiaceratherium magnum*, an early-branching rhinocerotid, as well as one of the earliest European rhinoceroses. We also compute a parsimony analysis based on morpho-anatomical characters to assess their phylogenetic position and elucidate the early evolution of the Rhinocerotidae. Our results allow to propose a new scenario for the reduction of the anterior dentition in which upper and lower dentitions would have undergone distinct evolutionary trajectories.

urn:lsid:zoobank.org:pub:C4732CCF-996F-48D1-AC33-C1E733CBDFD9

Keywords

Epiaceratherium, *Mesaceratherium,* New species, Rhinocerotidae, Oligocene, Phylogeny

Acknowledgements

We deeply acknowledge all curators and collection managers who have permitted us to study the specimens presented here and comparison specimens: Gertrud Rössner and Kurt Heissig (Bayerische Staatssammlung für Paläontologie und Geologie, Munich, Germany), Emmanuel Robert (Université Claude-Bernard-Lyon-I, France), Yves Laurent (Muséum d'histoire naturelle de Toulouse, France) and Loïc Costeur (Naturhistorisches Museum Basel, Switzerland). We thank Renaud Roch (JURASSICA Museum, Porrentruy, Switzerland) for restoring some specimens presented here. We are especially thankful to Ursula Menkveld-Gfeller and Bernard Hostettler (Naturhistorisches Museum der Burgergemeinde Bern, Switzerland) for helping us find a species name for the new species and for letting us borrow and study the material from Bumbach.

Authors' contributions

All authors contributed to the study conception and design. Material study and analysis were performed by all authors. The first draft of the manuscript was written by Damien Becker and Jérémy Tissier. All authors gave final approval for publication and agree to be held accountable for the work performed therein.

Funding

This study was funded by the Swiss National Science Foundation (grant 200021-162359).

Conflict of Interest

The authors declare that they have no conflict of interest.

Data availability

All data generated or analysed during this study are included in this published article (and its supplementary information files). The morphological data matrix is available in NEXUS format in Supplementary Material 1. 3D models will be available from the MorphoMuseuM repository with DOIs and are temporarily accessible at these urls: http://morphomuseum.com/Specimenfiles/sendFile/534/762cc9 (3D surface model of the mandible NMB.O.B.928) and http://morphomuseum.com/Specimenfiles/sendFile/535/f4de1d (3D surface model of the archetype reconstruction of *Epiaceratherium*).

Introduction

Rhinocerotoidea have a relatively recent history in Western Europe, compared to their long-lasting history in Asia and North America, where they developed since the Early and early Middle Eocene respectively [1,2]. Indeed, they first appear in Western Europe after the Eocene–Oligocene transition, and the related Grande Coupure event [3–6]. Five genera of rhinocerotoids are known in Western Europe during the Early Oligocene interval: the eggysodontid *Eggysodon*, the amynodontid *Cadurcotherium* and the rhinocerotids *Epiaceratherium*, *Molassitherium*, and *Ronzotherium*. Among these three families, only the Rhinocerotidae survived the Oligocene–Miocene transition and have living representatives, in Africa and Asia [7].

The first appearance of Rhinocerotidae in Europe is mostly explained by dispersals from Asia, documenting the Grande Coupure event [1,4,8–10]. Indeed, *Epiaceratherium* and *Ronzotherium* may have close late Eocene relatives in Asia [1,11,12]. In Europe, *Epiaceratherium* is only known from a few Early Oligocene localities [5,10,12–15] contrary to *Ronzotherium* which is a conspicuous element of mammalian assemblages throughout the Oligocene epoch [16,17]. *Epiaceratherium bolcense* Abel, 1910 [18] is documented by numerous remains from the earliest Oligocene locality of Monteviale in Italy [13,15] but it has never been recognised anywhere else. *Epiaceratherium magnum* Uhlig, 1999 [14] is mostly represented by isolated dental and postcranial remains from several Early Oligocene localities of Germany, France, Switzerland and Czech Republic, i.e. across the Molasse Basin [5,10,14]. Later on, several remains originally referred to as *Epiaceratherium* aff. *magnum* by Uhlig [14] were assigned to *Molassitherium delemontense* Becker & Antoine, 2013 [19]. The type species of *Molassitherium* Becker & Antoine, 2013 [19] is *Molassitherium albigense* (Roman, 1912) [20], from the late Early and early Late Oligocene of France and the Iberian Peninsula [19,21].

The first occurrence of *Mesaceratherium* Heissig, 1969, another European rhinocerotid, is recorded during the late Oligocene [16]. The type species *M. gaimersheimense* Heissig, 1969 [16] was first discovered in the South German locality of Gaimersheim, along with *Ronzotherium*, but has now been identified in France and Switzerland as well [22,23]. Two other species are reported from the earliest Miocene: *M. paulhiacense* (Richard, 1937) [24] from France and *M. welcommi* Antoine & Downing, 2010 [25] from Pakistan.

Here, we describe an unpublished mandible (NMB.O.B.928) from the “middle Oligocene” Rheinbetts locality (Basel Canton, Switzerland) and newly restored mandibular and postcranial remains from the Bumbach locality (MP25 reference level; Bern Canton, Switzerland). We also compute a parsimony analysis based on morpho-anatomical features and including well-documented early Holarctic rhinocerotids as terminals, in order to assess

their phylogenetic affinities and, more broadly, to enlighten the early evolutionary history of the Rhinocerotidae. This phylogenetic framework further allows for inferring the evolution of one of the major adaptative traits of this group, i.e. their anterior dentition (incisors and canines).

Material and methods

1. Specimens

The newly described mandible NMB.O.B.928 from Rheinbetts is housed in the Naturhistorisches Museum Basel, Switzerland (**NMB**) while the specimens from Bumbach attributed to *Molassitherium albigense* are housed in the Naturhistorisches Museum der Burgergemeinde Bern, Switzerland (**NMBE**). The abbreviations of other institutions are: **BSPG**, Bayerische Staatssammlung für Paläontologie und Geologie, Munich (Germany); **DGMV**, Department of Geology and Minerals of Vietnam, **MJSN**, Jurassica Museum of Porrentruy (Switzerland); **MHNT**, Muséum d'histoire naturelle de Toulouse (France); **FSL**, Université Claude-Bernard-Lyon-I (France).

2. Surface scanning

Specimens have been scanned with a structured-light surface scanner (Artec Space Spider, Artec Group) and the 3D models were reconstructed using the Artec Studio 13 Professional software. These 3D models are available in Tissier, Antoine and Becker (In Prep.) [26].

3. Terminology

The morpho-anatomical characters described here follow the terminology of Antoine [27]. The dental abbreviations are as follows: c/C, lower/upper canine; d/D, lower/upper decidual tooth; i/I, lower/upper incisor; m/M, lower/upper molar; p/P, lower/upper premolar. Dental measurements were taken according to Uhlig [14]. Measurements are given in millimetres and measurements between parentheses are estimated.

4. Characters matrix and phylogeny

The character matrix is available in Supplementary Material 1 and is based on Antoine [27] with six additional characters:

- 283: p3, lingual branch of the paralophid: 0, developed; 1, reduced
- 284: p3-4, anterolingual cingulid: 0, stopping at the anterior valley or absent; 1, joining metaconid
- 285: P2, metacone fold: 0, strong; 1, weak or absent

- 286: P3-4, metacone fold: 0, strong; 1, weak or absent
- 287: M1-2, parastyle: 0, long; 1, short
- 288: I1, shape = 0, spatulate; 1, conical and pointed; 2, chisel (ordered)

Parsimony analyses were computed with the software PAUP* Version 4.0a (build 167) [28]. All characters were set as ordered, except characters 72, 94, 102, 103, 140, 187, 190 which do not form morphoclines, and all characters have equal weights.

We modified characters 2 and 3 from the original matrix of Antoine [27] as follows:

- 2: Maxilla: foramen infraorbitalis: 0, above P1-2; 1, above P3; 2, above P4; 3, above molars
- 3: Nasal notch: 0, above P1-2; 1, above P3; 2, above P4-M1

Several analyses were performed to assess the identification of the mandible NMB.O.B.928 from Rheinbetts and the material from Bumbach. We tested different sets of terminals, adding taxa by incrementation, or by merging some specimens into a single terminal. If in the resulting trees several specimens were grouped into a single clade, with the holotype specimen, we merged them together. When merging the scores of these terminals, the differences in characters states are considered as polymorphism in the new coding. During the first analysis, the taxonomic sample included two non-rhinocerotid perissodactyls as outgroups (*Tapirus terrestris* and *Hyrachyus eximius*) and the ingroup consisted of *Epiaceratherium naduongense*, *Epiaceratherium bolcense*, and *Molassitherium delemontense*, as well as specimens attributed to *M. albigense*, specimens from Bumbach (scored in a single terminal) and the mandibular specimen from Rheinbetts (NMB.O.B.928). We first selected these taxa to test our a-priori referrals to these respective genera (*Epiaceratherium* and *Molassitherium*). New terminals were then added consecutively, documenting other taxa to which they could be referred as, especially within *Mesaceratherium* and *Pleuroceros*. Other taxa were added to test the monophyly of *Epiaceratherium* and *Molassitherium* and, more broadly to better understand the early evolutionary steps of Rhinocerotidae, as well as to stabilise the topology of the tree and to test its robustness.

A branch-and-bound search algorithm was first used for the analyses encompassing 12 to 16 terminals, after which the heuristic algorithm was faster and almost as efficient. The addition sequence was set to "furthest" during the branch-and-bound search. We used a random addition sequence of 1000 replicates and held 100 trees at each step during the heuristic search with a TBR swapping algorithm with no reconnection limit and swapping on all trees.

5. Nomenclatural act

The electronic edition of this article conforms to the requirements of the amended International Code of Zoological Nomenclature, and hence the new names contained herein are available under that Code from the electronic edition of this article. This published work and the nomenclatural acts it contains have been registered in ZooBank, the online registration system for the ICZN. The ZooBank LSIDs (Life Science Identifiers) can be resolved and the associated information viewed through any standard web browser by appending the LSID to the prefix "http://zoobank.org/". The LSID for this publication is: urn:lsid:zoobank.org:pub:C4732CCF-996F-48D1-AC33-C1E733CBDFD9. The electronic edition of this work was published in a journal with an ISSN, and has been archived and is available from the following digital repositories: CLOCKSS, LOCKSS, Portico and PubMed Central.

Systematic palaeontology

Perissodactyla Owen, 1848 [29]

Rhinocerotoidea Owen, 1845 [30]

Rhinocerotidae Gray, 1821 [31]

Epiaceratherium Abel, 1910 [18]

Type species: *Epiaceratherium bolcense* Abel, 1910 [18]

Other species: *Epiaceratherium magnum* Uhlig, 1999 [14]; *Epiaceratherium delemontense* comb. nov. (Becker & Antoine, 2013) [19]; *Epiaceratherium naduongense* Böhme et al., 2013 [12].

Diagnosis: Stem rhinocerotine lacking i3 and a lower canine, with a wide postfossette on P2-P4, a protoloph usually constricted on M1-M2, a straight posterior half of the ectoloph on M1-M2, and a posterior valley usually closed on p2.

Stratigraphical distribution: late Middle Eocene to Early Oligocene (South Asia) and Early to early Late Oligocene (Europe).

Geographical distribution: From East to West**,** Northern Vietnam, Pakistan, Czech Republic, Northern Italy, Germany, Switzerland, and France.

Epiaceratherium magnum Uhlig, 1999 [14]

Emended diagnosis: large species of the genus with a horizontal mandibular symphysis with divergent i2, a metacone fold weak or absent on P2, a crista sometimes present on P3, a metaloph directed postero-lingually on P3-4, a crochet usually present on upper molars, a crista usually absent on upper molars, a V-shaped ectolophid groove developed until the neck on lower cheek teeth, a single-rooted d1, an antero-posterior diameter/height ratio inferior to 0.65, and a nearly straight caudal border of the astragal trochlea. The skull is unknown.

Type material: Left M1 (BSPG-1972-XI-1930), M2 (BSPG-1972-XI-1930) and M3 (BSPG-1972-XI-1930).

Type horizon and locality: Fissure filling of Möhren 13 near Treuchtlingen (Franken Alb, Bavaria, Germany), possibly MP22 (early Oligocene) based on faunal comparison.

Additional referred material: NMB.O.B.928 (Fig. 1), a sub-complete mandible from the "Molasse Alsacienne" formation of Rheinbetts (Basel Canton, Switzerland), dated from the "Middle Oligocene".

Stratigraphical distribution: Early to early Late Oligocene.

Geographical distribution: Germany, France and Switzerland. *Epiaceratherium* cf. *magnum* was described in lower Oligocene deposits of Pakistan [1].

Description. The mandible NMB.O.B.928 (Fig. 1) is both well preserved (left ramus) and partly reconstructed (right ramus). The angle between the symphysis and the *corpus mandibulae* is particularly open, as the symphysis is almost horizontal. The symphysis is wide and without ventral keel. The dorsolateral borders of the symphysis, constricted, form sharp and acute dorsal ridges, between incisors and cheek teeth. The posterior edge of the symphysis is located at the level of p2. There are six *mental foramina* on each side: the two most anterior are large and ventral, whereas four others are more lateral and smaller. On the left side, the four smaller foramina are located below p1-2, whereas on the right two are located below p2-3 and other ones are anterior to p1. On the medial side of the *corpus mandibulae*, the groove for the mylohyoid nerves and vessels is well marked and extends from m1/2 to the ramus. The ventral edge of the corpus is completely straight in lateral view. The ramus is reconstructed on the right side, but partly preserved on the left. It is vertical, with a well-developed coronoid process. The large *foramen mandibulare* opens slightly below a hypothetical horizontal line formed by the teeth neck.

The lower dental formula is i1, i2, d/p1-m3. The premolar series is long compared to the molars (Lp3-4/Lm1-3 > 0.5). The cement is globally absent on cheek teeth. The first incisors are typically incisor-shaped and well developed, with a clear neck and a thin lingual cingulid. They are partly worn by contact with upper teeth (and

food) and almond shaped in occlusal outline. The very large second incisors are tusk-like and slightly divergent. They are also very worn, without a clear neck. The wear surface is triangular, with a much more worn medial side. There is no cingulid.

The p1 is absent on both sides, but there are small alveoli, anterior to p2, for a small single-rooted d/p1. There are very thin and smooth external rugosities on the ectolophid of p2-3. The ectolophid groove of p2-m3 is angular but vanishing above the neck. On p3, this groove is very oblique, and becomes almost horizontal above the neck. The trigonid of the cheek teeth is angular and forms an acute angle. The entoconid and metaconid are not constricted. The posterior valley of p2 is lingually closed, while on p3-4 it is very narrow and V-shaped, in lingual view. The lingual cingulid is completely absent on p2-m3. The labial cingulid is only partly present on m2-3, but very weak. The paralophid of p2 is developed, isolated, and spur-like. The hypolophid of the lower molars is oblique. There is no lingual groove on the entoconid of m3.

Teeth	*E. magnum* NMB.O.B.928	*E. magnum* [14]	*E. bolcense* (NMB.I.O.23)	*E. naduongense* (DGMV SAU-08)
**i1**	13-12.7 / 9.3-8.6 / 10-9.5	11-14 / 9-12 / x-x		11 / 8
**i2**	27.6-27.7 / 17-16.5 / x-x	23-24 / 14-15.5 / x-x		25 / 17
**p2**	20-21.5 / 13-14	18.5-22 / 12-14.5	18.5 / 10.7	22 / 13
**p3**	24-24.5 / 18.5-17.5	24-27.5 / 16-20	21.1-20.5 / 14-14.5	27 / 18
**p4**	26.5-26 / x-20	25-29 / 18-22.5	22.5 / 16.1	27 / 21.5
**m1**	25.5-27.5 / 20.5-20.5	27-31.5 / 21-25	24.6 / (18.3)	31 / 22.5
**m2**	34-35.5 / 24-24	31.5-36 / 22-26.5	28.2 / 19.3	35 / 24
**m3**	36-37 / 21-23	31-36.5 / 21-24.5		37 /24

Table 1: Measurements of the lower teeth of NMB.O.B.928 compared with other species of *Epiaceratherium*. Measurements are given in millimetres and are presented as width (or APD for incisors) / length (or TD for incisors) / height.

Comparisons. The mandible NMB.O.B.928 can be unambiguously attributed to a rhinocerotoid, based on the typical shape of the lower cheek teeth (ectolophodont cheek teeth with a *cristid obliqua* directed towards the protoconid). An attribution to the Amynodontidae, Eggysodontidae, Hyracodontidae, or Paraceratheriidae can be excluded based on its anterior dentition, due to the tusk-shaped second lower incisor and the absence of third lower incisor and lower canine. The mandible can be further referred to a small to middle-sized rhinocerotid, thus excluding large-sized European Oligocene rhinoceroses such as *Ronzotherium* or *Diaceratherium*.

Six genera of small- to medium-sized Rhinocerotidae are known in Europe during the Oligocene-Early Miocene. *Pleuroceros*, *Plesiaceratherium* and *Protaceratherium* (sensu [19]) are all reported from the ?latest Oligocene to the Early Miocene [32]. *Pleuroceros* differs from NMB.O.B.928 in having a smooth and U-shaped external groove on lower cheek teeth (angular and V-shaped on NMB.O.B.928) and a continuous lingual cingulid on

lower premolars (absent on NMB.O.B.928) [25]. *Plesiaceratherium* differs from the studied specimen in having premolars with a labial cingulid high above the base (absent on NMB.O.B.928), with shallow ectolophid groove (angular and V-shaped on NMB.O.B.928) [33]. *Protaceratherium* differs by the reduced first lower incisors (strong on NMB.O.B.928) and the presence of cingulid on the lower cheek teeth (mostly absent on NMB.O.B.928) [34]. Among Oligocene taxa, *Mesaceratherium* differs by its very upraised mandibular symphysis (horizontal on NMB.O.B.928) and the usual presence of lingual cingulid on the lower cheek teeth (absent on NMB.O.B.928) [16]. *Molassitherium albigense* (lower dentition unknown for *M. delemontense*) differs by the strong cingulid on the lower cheek teeth (mostly absent on NMB.O.B.928) [17]. To sum up, the mandible can be assigned to *Epiaceratherium*, as supported by our phylogenetic analyses. Both entities share the absence of i3 and c, the absence of lingual cingulid on the lower premolars, as well as external rugosities on the ectolophid of the premolars and the usually-closed posterior valley on p2, which are diagnostic characters shared by all species of the genus [12]. Within this genus, *E. naduongense* and *E. bolcense* differ by the biradiculate d/p1 whereas it is uniradiculate on NMB.O.B.928, as in *E. magnum* [12,14]. *Epiaceratherium bolcense* further differs by the constriction of the metaconid on the cheek teeth [13] whereas *E. naduongense* differs by a slightly more convex ventral border of the mandible and a slightly more upraised symphysis [12]. Therefore, we refer to the mandible NMB.O.B.928 as *Epiaceratherium magnum*.

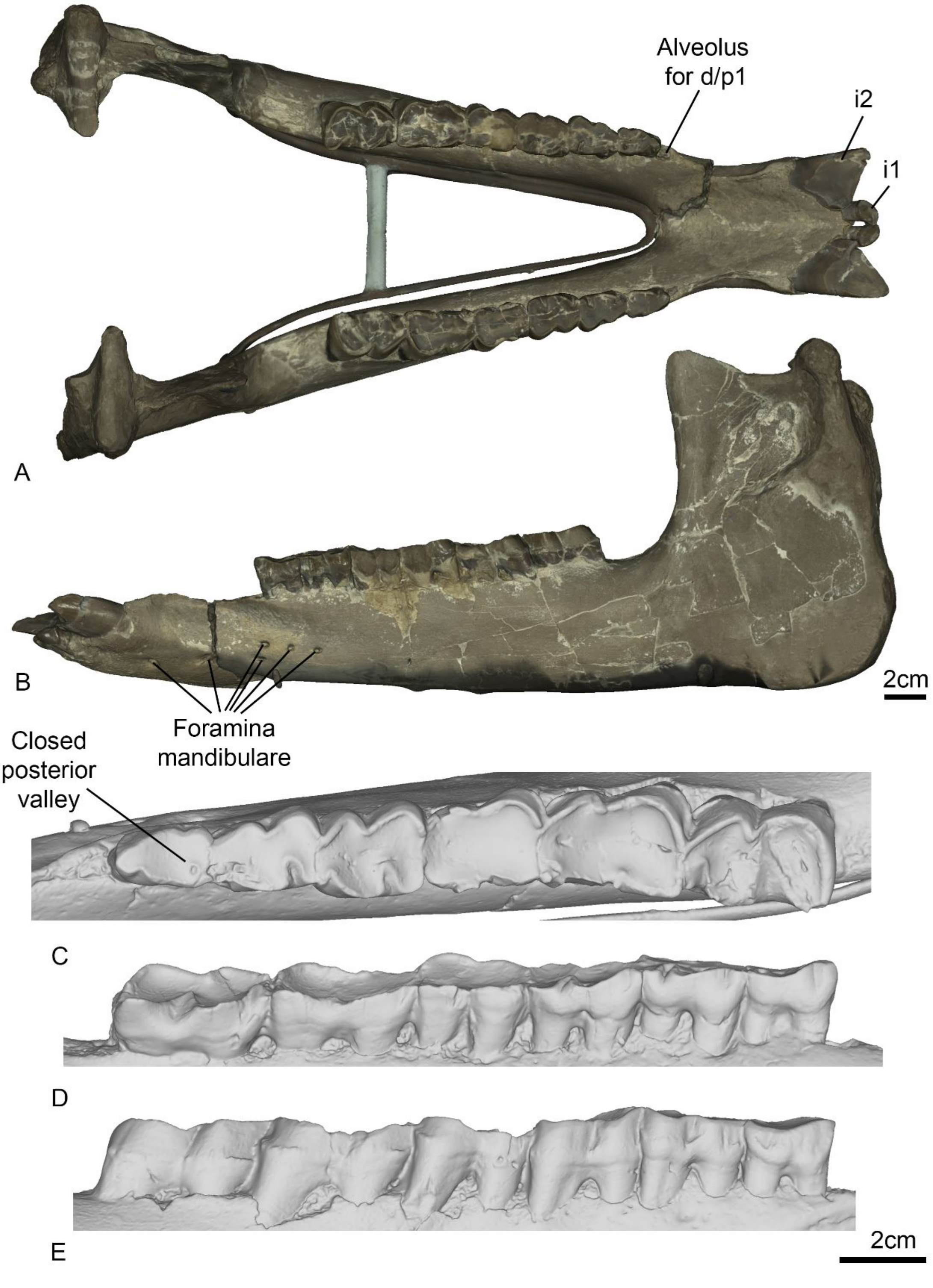

Figure 1: 3D models of the mandible of the Oligocene rhinocerotid *Epiaceratherium magnum* NMB.O.B.928, from Rheinbetts, Switzerland. A: occlusal view with texture; B: lateral view with texture; C: close-up occlusal view of the right cheek teeth series, with p2-m3; D: same, in lingual view (note the distolingually-twisted wear); E: same, in labial view. Scale bars equal 2 cm.

Rhinocerotinae Gray, 1821 [31]

Mesaceratherium Heissig, 1969 [16]

Type species: *Mesaceratherium gaimersheimense* Heissig, 1969 [16]

Other species: *Mesaceratherium paulhiacense* (Richard, 1937) [24]; *Mesaceratherium welcommi* Antoine & Downing, 2010 [25]; *Mesaceratherium tschani* sp. nov.

Diagnosis: Medium-sized hornless rhinocerotine, with a strong paracone fold on M1-M2, a posterior McIII-facet on McII, no posterior MtII- facet on MtIII, with slender limbs, a transverse metaloph on P2, a lingual cingulid on lower premolars, and a curved magnum-facet on McII.

Stratigraphical distribution: MP25–MN3 (emended from [22]).

Geographical distribution: France, Switzerland, Germany, Pakistan [16,22–25,32,35].

***Mesaceratherium tschani* sp. nov.**

urn:lsid:zoobank.org:act:C3E9079B-5DE8-4C50-9C78-ACE7DEF8C2DB

Diagnosis: Differs from all other assigned species by a ramus of the mandible inclined forward, a developed external groove on the lower cheek teeth, the absence of labial cingulid on the lower molars, radius and ulna with a marked contact on the diaphysis and the presence of a posterior expansion of the pyramidal facet on the unciform. Skull and upper dentition unknown.

Further differs from *M. gaimersheimense* in having a posterior border of the mandibular symphysis at the level of p2, a contact between the pyramidal and McV facets on the unciform, a keeled anterior side on the lunate and a pentagonal uniform facet on the McIV.

Differs from *M. welcommi* by a posterior border of the mandibular symphysis at the level of p2, lower cheek teeth without a constricted entoconid, an ectolophid groove deep to angular and not interrupted above the neck, an angular and acute trigonid, a rounded distal border of the lunate and a magnum-facet does not reach the anterior side of the lunate.

Differs from *M. paulhiacense* by the large pyramidal-facet on the unciform, the contact between the McV and pyramidal facets on the unciform, the circular posterior facet for the McIV on the McIII and the small-sized McV facet on the McIV.

Etymology: From the last name of the original person who discovered these specimens, Gottlieb Tschan, from Merligen (Bern Canton, Switzerland).

Type locality and horizon: Bumbach (Bern Canton, Switzerland), Swiss reference level for the MP25 biozone (early Late Oligocene).

Type material: Fragmented mandible NMBE–D1836/1837 with left p3 and m3 and right p1-3 and labial sides of p4-m2.

Other referred material: Right mandible with p4–m1 (NMBE–D1823); mandible (NMBE–D1836/1837); atlas (NMBE–D217); fragments of thoracic vertebra (NMBE–D161; NMBE–D220); distal fragments of right and left humeri (NMBE–D214, NMBE–D215); right radius (NMBE–D224); left radius-ulna (NMBE–D223); left semilunate and scaphoid (NMBE–D1546, NMBE–D1547); right unciform (NMBE–D1559); proximal fragment of right McIII (NMBE–D1567); left McIV (NMBE–D1563); all specimens from Bumbach (Bern Canton, Switzerland), dated from MP25. Figs. 2-3.

Description: The material from Bumbach is poorly preserved, sliced, and crushed. Both sides are preserved on the mandible NMBE-D1836/1837 (Figs 2A-D). Only p3 and a part of m3 are preserved on the left side, whereas p1-3 and labial parts of p4-m2 are preserved on the right side. The symphysis is broken, but it is nonetheless upraised compared with the *corpus mandibulae*. The symphysis is quite wide, and its posterior border is located at the level of p2. As on the previously described mandible NMB.O.B.928 from Rheinbetts, there are several mental foramina on each side. On the right side, two large ones are at the level of p1/2 and p3 and a much smaller one is between p1 and i2. On the left side, three large foramina are at the level of p1/2 and a smaller one below p2. However, the symphysis is very badly preserved, and some parts are missing. The lingual groove for the mylohyoid nerves and vessels seems to be well marked on the corpus, but it could also be an artefact from the restoration. The ventral base of the *corpus mandibulae* is straight. On the other mandible available, NMBE-D1823 (Figs. 2E-F), only the right part is preserved, with p4 and a part of m1. The symphysis is broken, but the ramus is preserved, and it is inclined forward and upward. The coronoid apophysis is broken and the *foramen mandibulare* is located below the teeth neck. The premolar series seems long compared to the molar series, but it is difficult to estimate precisely the exact length of the latter. Cement is completely absent. Only the root of one i2 is partly preserved on NMBE-D1836/1837 (Fig. 2A-D). Based on the fragmentary and heavily crushed remaining part of the symphysis, i1s were probably absent. The third incisor and the canine are also absent. The p1 is present, large and single-rooted. There are very weak vertical rugosities on the anterior part of the

ectolophid of p2-3. The ectolophid groove of the cheek teeth is deep to angular and does not vanish above the neck on m2 but does on premolars. The trigonid is angular and forms an acute angle in occlusal view. The opening of the posterior valley is acute on premolars, and the lingual cingulid is very weak, but present below the anterior valley. Labial cingulid is present on the trigonid, and below the ectolophid groove. The paralophid of p2 is curved, without constriction, and developed. The posterior valley of p2 is lingually open. The labial cingulid is present on the trigonid of m3. No other characters are visible on the molars.

Teeth	***Mesaceratherium tschani* sp. nov. NMBE–D1823**	***Mesaceratherium tschani* sp. nov. NMBE–D1836/1837**	***Mesaceratherium gaimersheimense* (from Gaimersheim)**	***Mesaceratherium gaimersheimense* (from Thézels)**	***Mesaceratherium welcommi***
**p1**		16 / 8.5	14 / x	15.3-17 / 8-9.6	
**p2**		23 / 17.5	22-23 / 12-14	20.6-23.6 / 11.8-15.5	
**p3**		27.5-27.5 / 19.5-21.5	27-30.5 / 18.5-20.5	27-30.3 / 19.4-22	25 / 22.5
**p4**	31.5 / 21	31 / x	28-30.5 / 20-22.5	27.6-33 / 21-23.9	32 / 30.5-32
**m1**			29-34.5 / 22-26.5	30-33.7 / 20.5-24	31-41 / 27.5-33
**m2**		(>30) / x	32-36.5 / 21.5-26	31.5-38 / 23-26	39.5–49.5 / 29.5–32
**m3**		(>35) / (25)	34 / 22.5	33-39 / 21.3-22.7	46.5–53.5 / 26–27.5

Table 2: Measurements of the lower teeth of the Oligocene rhinocerotine *Mesaceratherium tschani* sp. nov. from Bumbach, compared with other *Mesaceratherium* species. Measurements are given in millimetres and are presented as length / width.

The postcranial elements are slender and small-sized (Fig. 3; Tab. 2-4). The atlas NMBE–D217 (Fig. 3A-B) is partly preserved, lacking the processus transverse (Ltot: (115.5); Htot: 65.0; H *foramen vertebrale*: 47.0; W condylar facets: 83.5). The rachidian canal has a mushroom-like outline. The alar notches are seemingly absent, while the *foramen vertebrale lateralis* and the *foramen transversarium* are present. They are hidden by the axis-facets in lateral view. The condylar facets are kidney-shaped, while the axis facets are transversally straight. The dimensions of the body (APD: 31.0; DTant: 35.5; DTpost: 37.0; Hant: 40.0; Hpost: 40.0) and the vertebral foramen (DT: 22.0; H: 23.0) of the thoracic vertebra NMBE–D161 (Fig. 3C) fit well those of the atlas NMBE–D217. Only the spinous process is observable from the deformed and incomplete thoracic vertebra NMBE–D220 (Fig. 3D).

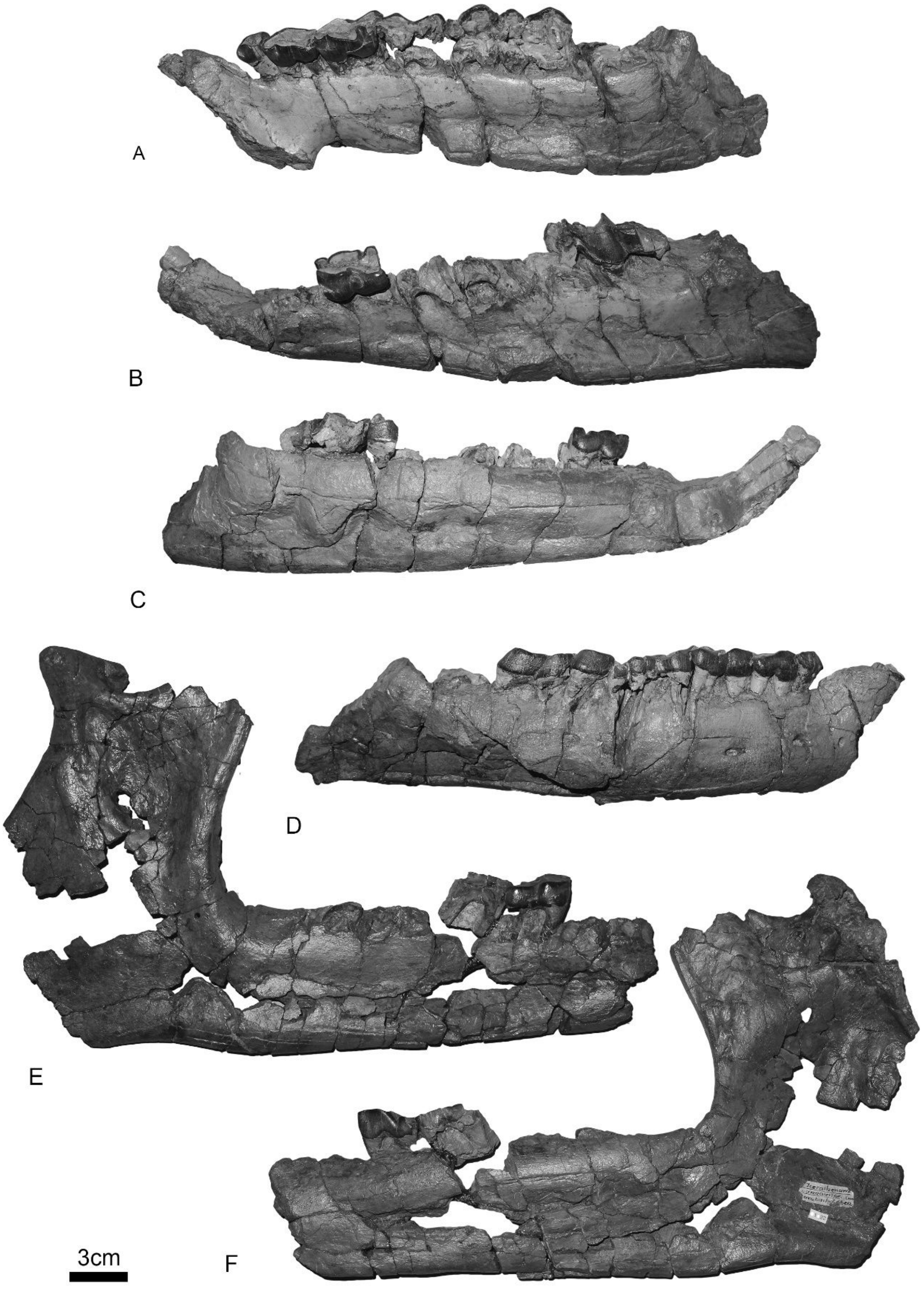

Figure 2: Mandibles of *Mesaceratherium tschani* sp. nov. from Bumbach (Switzerland; MP25). A-D: mandible NMBE–D1836/1837; E-F: mandible NMBE–D1823. Scale bar equals 3 cm.

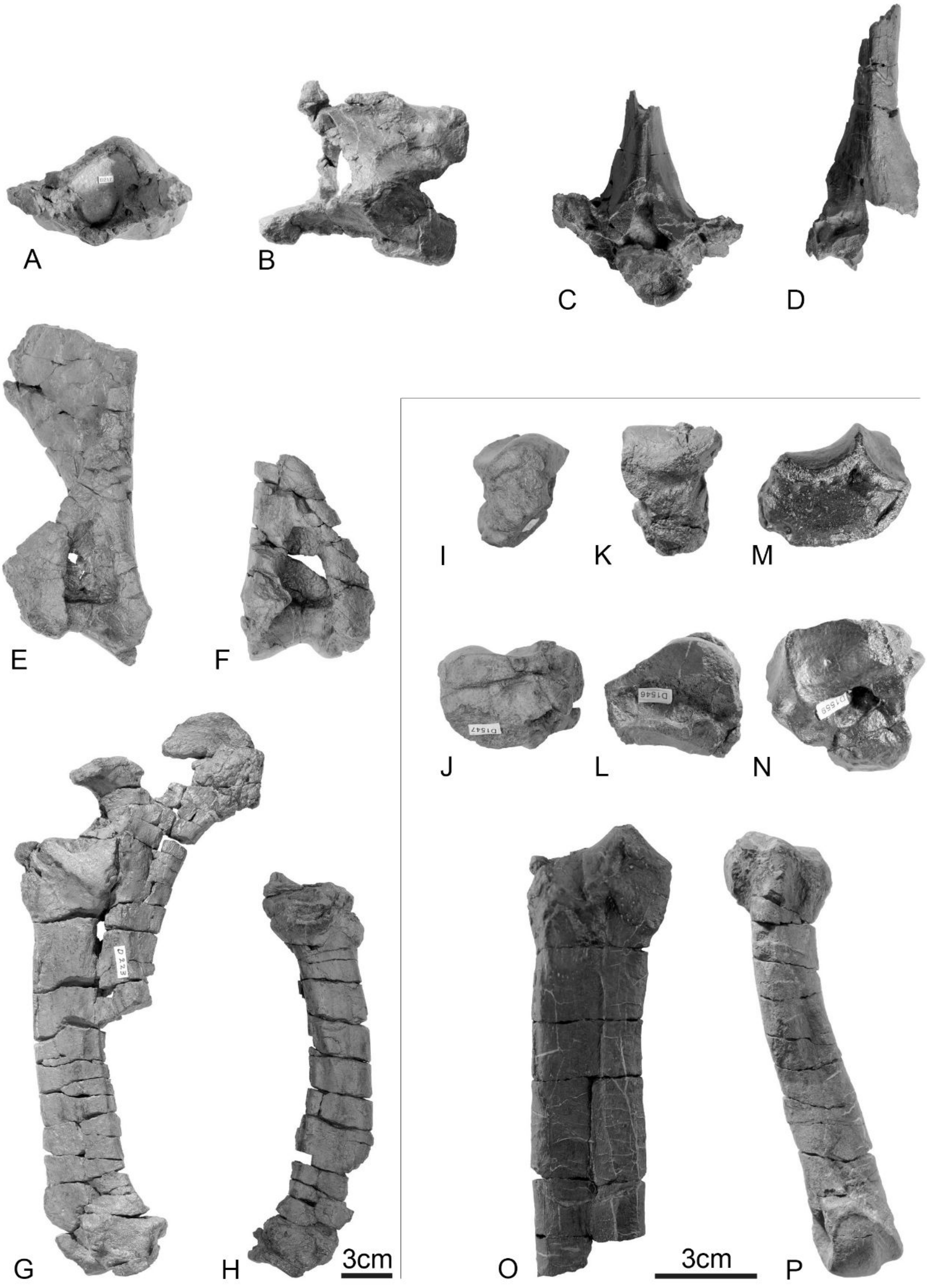

Figure 3: Postcranial remains of the Oligocene rhinocerotine *Mesaceratherium tschani* sp. nov. from Bumbach (Switzerland; MP25). A-B: atlas NMBE–D217; C: thoracic vertebra NMBE–D161; D: thoracic vertebra NMBE–D220; E: left humerus NMBE–D215; F: right humerus NMBE–D214; G: left radius-ulna NMBE–D223; H: right radius NMBE–D224; I-J: fragment of left scaphoid NMBE–D1547; K-L: left lunate NMBE–D1546; M-N: right unciform NMBE–D1559; O: right McIII NMBE–D1567; P: left McIV NMBE–D1563. Scale bars equal 3 cm.

Two distal fragments of humerus (NMBE–D214 and NMBE–D215; Fig. 3E-F) are referred to this taxon. The diaphysis is slender. The humeral crest forms a right dihedron with the epicondylar crest. The *fossa olecrani* is narrow and high. The trochlea is poorly constricted in its median part (egg cup *sensu* [27]). The medial lip is more developed. The lateral epicondyle is rather low, moderately developed, and bearing a shallow distal gutter. There is no scar (synovial fossette) on the anteroproximal part of the trochlea.

The radius is represented by two deformed specimens, but nearly complete. NMBE–D223 (Fig. 3G) includes also the proximal part of the ulna. The latter is not completely in anatomical connection, the radius being rotated by about counter clockwise 90° and the effective welding is due to taphonomical process. However, it shows clear synostosis traces resulting from a contact radius/ulna. From the radius NMBE–D223 (Fig. 3H), the anterior border of the proximal articulation is straight, the medial border of the diaphysis is rather straight, the *m. extensor carpi* groove is wide and deepened by the strong *tuberculum dorsale* lying beside it, and the posterior expansion of the scaphoid-facet is low. On NMBE–D224, the proximal ulna-facets are clearly separate and the insertion of the *m. biceps brachii* is shallow. From the ulna, the olecranon is thick, forming a rather closed angle with the diaphysis. The posterior tip of the olecranon (insertion of the *m. triceps brachii*) is salient with respect to the distal border of the process. By its dimensions, the humeral cochlea fits the distal trochlea of the humerus NMBE–D215.

The carpus is slender. Both the left scaphoid (NMBE–D1547; Fig. 3I-J) and lunate (NMBE–D1546; Fig. 3K-L) are posteriorly incomplete but they fit together with the radius-ulna (NMBE–D223) and belong probably to the same individual. The posteroproximal facet of the scaphoid for the lunate is lacking, but the two bones are posteroproximally in contact. The trapezium-facet and the magnum-facet are not visible from the scaphoid. The lunate has no ulna-facet. The anterior side is smooth, with a rounded distal border. The magnum-facet does not reach the anterior side. In lateral view, the distal pyramidal-facet is elliptic and anteriorly elongated by a thin band stretching to the anterior border. The unciform-facet is sagittally elongated.

The unciform NMBE–D1559 (Fig. 3M-N) is well preserved. The proximal facets are separate by an acute ridge. The semilunate-facet roughly outlines a quarter-circle nearly flat. The pyramidal-facet is larger, slightly concave transversally and regularly convex sagittally. There is a wide postero-lateral expansion to this facet, which connects it to the McV-facet. The distal facets (for the magnum, McIII and McIV) are not distinct, except the most lateral one, for the McV. In anterior view, the laterodistal border of the bone is straight (McV-facet) while the rest of the distal border is rounded. This McV-facet is subvertical pointing to a tridactyl manus.

The proximal fragment of McIII NMBE–D1567 (Fig. 3O) points to a very slender bone. In dorsal view, the lateral articular facets fit with the McIV NMBE–D1563, which leads us to assign it to the same individual. The insertion of the *m. extensor carpalis* is salient and the magnum-facet is largely visible in anterior view. The latter is regularly concave transversally and separate from the unciform-facet by a sharp ridge (90° angle). The proximal end is wide, due to the strong lateral development of the unciform-facet. The latter is wide, slightly sagittally convex and triangular. The surfaces for the *m. interossei* are restricted to the proximal half of the diaphysis on the medial and lateral sides. The McIV-facets are well developed and separate. The anterior one is elliptic and the posterior one is circular. It is distally displaced with respect to the proximal end of the bone. The diaphysis is straight and anteroposteriorly flattened. The McII-facet is not observable on the available specimen.

The McIV NMBE–D1563 (Fig. 3P) is slender (TDdia/L = 0.144). Its proximal facet is pentagonal and sagittally elongated in proximal view. On the medial side, the McIII-facets are separate by a deep groove. The anterior one is elliptic and subhorizontal, while the posterior one is circular and lower. The diaphysis is curved laterally, at the proximal third of the bone. The *m. interosseus* is restricted to the proximal half of the diaphysis. The diaphysis is slightly sagittally flattened. The intermediate relief is high and acute. The medial lip is lower than the lateral one. The latter is transversally concave whilst the former is flat. In lateral view, the proximal end bears a small articular McV-facet.

Postcranials	L	TDprox	APDprox	TDdia	APDdia	TDdist	APDdist
**Humerus** NMBE-D214 (*Mesaceratherium tschani* sp. nov.)				39.5	44	81	75.5
**Humerus** NMBE–D215 (*Mesaceratherium tschani* sp. nov.)				38	41	88	(66.5)
**Humerus** from Thézels (*Mesaceratherium gaimersheimense*)	298.1	109.8		39.2-49.9	44.7-49.4	82.2-99.1	68-73.2
**Radius** NMBE–D219 (*Mesaceratherium tschani* sp. nov.)				47	34.5		
**Radius** NMBE–D222 (*Mesaceratherium tschani* sp. nov.)				50.5	32		
**Radius** from Thézels (*Mesaceratherium gaimersheimense*)		60.9-70	34.7-38.2			70.8	
**Ulna** NMBE–D223 (*Mesaceratherium tschani* sp. nov.)	326.0	40.5	109.5	18.0	37.0		
**Ulna** The4338 from Thézels (*Mesaceratherium gaimersheimense*)			112.6	25.2	46.5		
**McIII** NMBE–D1567 (*Mesaceratherium tschani* sp. nov.)	>133.0	(42.0)	>27.0	33.5	12.0		
**McIII** from Thézels (*Mesaceratherium gaimersheimense*)	153-172.9	32.1-40.5	22.3-32	30.2-37.8	11.8-15	40.3-50.7	25-29.5
**McIV** NMBE–1563 (*Mesaceratherium tschani* sp. nov.)	135.0	29.5	27.0	19.5	11.0	23.5	24
**McIV** The4117 from Thézels (*Mesaceratherium gaimersheimense*)		23	31.7				

Table 3: Measurements of the long bones and metacarpals of the Oligocene rhinocerotine *Mesaceratherium tschani* sp. nov. from Bumbach compared with *Mesaceratherium gaimersheimense*, in millimetres.

Postcranials	APD	TD	H
**Scaphoid** NMBE–D1547 (*Mesaceratherium tschani* sp. nov.)	43	32.5	33.5
**Scaphoid** from Gaimersheim (*Mesaceratherium gaimersheimense*)	53.9	34.5	42.6
**Scaphoid** from Thézels (*Mesaceratherium gaimersheimense*)	55.5-62.1	30.8-35.3	48.6-54.7
**Unciform** NMBE–D1559 (*Mesaceratherium tschani* sp. nov.)	48.8	45.5	32
**Unciform** BSPG-1952-II from Gaimersheim (*Mesaceratherium gaimersheimense*)	50.5	47	37.5
**Unciform** from Thézels (*Mesaceratherium gaimersheimense*)	60.4-65.4	42.6-50.9	34.8-42.8
**Lunate** NMBE–D1546 (*Mesaceratherium tschani* sp. nov.)	>40.0	30.0	36.0
**Lunate** BSPG-1952-II from Gaimersheim (*Mesaceratherium gaimersheimense*)	39.6	29.2	31.1
**Lunate** from Thézels (*Mesaceratherium gaimersheimense*)	57.9-67.9	32.6-40.6	40.8-44.7

Table 4: Measurements of the carpal bones of the Oligocene rhinocerotine *Mesaceratherium tschani* sp. nov. from Bumbach compared with *Mesaceratherium gaimersheimense*, in millimetres.

Comparisons. As for the specimen from Rheinbetts, these specimens from Bumbach can be unambiguously attributed to a rhinocerotid, based on the typical shape of the lower cheek teeth (ectolophodont cheek teeth with a *cristid obliqua* directed towards the protoconid) and the anterior dentition (absence of i3 and c, and tusk-shaped i2). It can also be further referred to a small to medium-sized rhinocerotid, thus excluding large-sized European Oligocene rhinoceroses such as *Ronzotherium* or *Diaceratherium*.

Among the six genera of medium-sized Rhinocerotidae known in Europe during the Oligocene–Early Miocene interval. *Pleuroceros* differs by a nearly horizontal mandibular symphysis (upraised on NMBE–D1836/1837) a smooth and U-shaped external groove on lower cheek teeth (deep to angular on NMBE–D1823 and NMBE–D1836/1837) and a continuous lingual cingulid on lower premolars (reduced on NMBE–D1823 and NMBE–D1836/1837) [25]. *Plesiaceratherium* differs by premolars with shallow ectolophid groove (deep to angular on NMBE–D1823 and NMBE–D1836/1837) [27,33]. *Protaceratherium* differs by the narrower symphysis in dorsal view (wider on NMBE–D1836/1837), the smoother ectolophid groove of the cheek teeth (deep to angular on NMBE–D1823 and NMBE–D1836/1837) and the stronger cingulid (usually absent on NMBE–D1823 and NMBE–D1836/1837) [19,34]. *Molassitherium albigense* (lower dentition unknown for "*M.*" *delemontense*)

differs by the much stronger cingulid on the lower cheek teeth (usually absent on NMBE–D1823 and NMBE–D1836/1837) [17,19]. *Epiaceratherium* differs by a posterior valley usually closed on p2 (open on NMBE–D1836/1837) and the absence of lingual cingulid on the lower premolars (present on NMBE–D1836/1837) [12]. In agreement with our phylogenetic results, we refer the material from Bumbach to *Mesaceratherium*. *Mesaceratherium* shares with these specimens a lingual cingulid on lower premolars, an angular ectolophid groove of the cheek teeth and a rather upraised mandibular symphysis [16,22,25,35]. Furthermore, the postcranial remains from Thézels assigned to *M. gaimersheimense* also share with the specimens from Bumbach the absence of scar on the trochlea, the egg-cup shaped trochlea and the high *fossa olecrani* of the humerus, the straight anterior border of the proximal articulation and medial border of the epiphysis of the radius, the closed angle between the olecranon of the ulna and the diaphysis, the triangular facet for the unciform on the McIII, and the overall similar dimensions (Tab. 3-4) but differ by other characters, such as a possibly tetradactyl manus, the remote pyramidal and McV facets on the unciform or the keeled anterior side of the lunate [22]. Therefore, based on this unique combination of characters, we refer to these specimens from Bumbach as *Mesaceratherium tschani* sp. nov.

Results

1. Phylogeny

The different sets of taxonomic samples used during the phylogenetic analyses and their results are reported in Table 5. The resulting trees of each analysis are presented in Supplementary Material 2.

**Analysis**	**Search algorithm**	**Taxonomic samples**	**Results**
1	Branch-and-bound	MHNT-PAL-2010-18-1; FSL-8543; FSL-8486; FSL-8544; specimens from Bumbach (one single terminal), NMB.O.B.928	All specimens from Moissac and St-Henri/St-André can be referred to *Molassitherium albigense.* NMB.O.B.928 should be referred to *Epiaceratherium.*
2	Branch-and-bound	Addition of *Mesaceratherium gaimersheimense* and *M. welcommi*	All specimens from Moissac and St-Henri/St-André can be referred to *Molassitherium albigense.* NMB.O.B.928 should be referred to *Epiaceratherium magnum.*
3	Branch-and-bound	Addition of *Pleuroceros pleuroceros* and *P. blanfordi*	All specimens from Moissac and St-Henri/St-André can be referred to *Molassitherium albigense.* NMB.O.B.928 can be referred to *Epiaceratherium magnum.* Bumbach closer to *Pleuroceros*/*Mesaceratherium.*
4	Branch-and-bound	**MHNT-PAL-2010-18-1, FSL-8543, FSL-8486 and FSL-8544 merged (= *M. albigense*)**	*M. delemontense* not sister group of *M. albigense.* Bumbach closer to *Pleuroceros*/*Mesaceratherium.*
5	Branch-and-bound	**NMB.O.B.928 and *Epiaceratherium magnum* merged**	*M. delemontense* not sister group of *M. albigense.* Bumbach closer to *Pleuroceros*/*Mesaceratherium.*

6	Branch-and-bound	Addition of *Uintaceras radinskyi*	*M. delemontense* not sister group of *M. albigense.* Bumbach closer to *Pleuroceros*/*Mesaceratherium.*
7	Branch-and-bound	Addition of *Teletaceras radinskyi*	*M. delemontense* not sister group of *M. albigense.*
8	Branch-and-bound	Addition of *Penetrigonias dakotensis*	*M. delemontense* not sister group of *M. albigense.*
9	Branch-and-bound	Addition of *Trigonias osborni*	*M. delemontense* not sister group of *M. albigense.*
10	Heuristic	Addition of *Amphicaenopus platycephalus*	*M. delemontense* not sister group of *M. albigense.* Bumbach closer to *Pleuroceros*/*Mesaceratherium.*
11	Heuristic	Addition of *Subhyracodon occidentalis*	*M. delemontense* not sister group of *M. albigense.*
12	Heuristic	Addition of *Protaceratherium minutum*	*Epiaceratherium* monophyletic (including *M. delemontense*) sister-group of *Teletaceras* and *Uintaceras*. Specimens from Bumbach sister-group of *Protaceratherium* and *Pleuroceros*
13	Heuristic	Addition of *Diceratherium armatum*	Specimens from Bumbach sister-group of *Mesaceratherium*
14	Heuristic	Addition of *Ronzotherium filholi*	
15	Heuristic	Addition of *Aceratherium incisivum*	Bumbach sister-group of *Aceratherium*
16	Heuristic	Addition of *Teleoceras fossiger*	Bumbach sister-group of *Aceratherium*
17	Heuristic	Addition of *Brachypotherium brachypus*	Bumbach sister-group of *Mesaceratherium*
18	Heuristic	Addition of *Prosantorhinus douvillei*	Bumbach sister-group of *Mesaceratherium*
19	Heuristic	Addition of *Alicornops simorrense* and *Plesiaceratherium platyodon*	
20	Heuristic	Addition of *Lartetotherium sansaniense*	Bumbach sister-group of *Mesaceratherium*
21	Heuristic	Addition of *Mesaceratherium paulhiacense*	*Mesaceratherium* monophyletic, including Bumbach

Table 5: Taxonomic sample used for each analysis, with details on the search algorithms used and a summary of corresponding results. The resulting most parsimonious trees, or consensus trees are available in Supplementary Material 2, where page numbers correspond to the analysis number in the first column.

Analyses 1 to 5 were done in order to test different hypotheses:

1) whether the specimens from St-Henri/St-André and Moissac (described by Lihoreau et al. [36]) all belonged to *Molassitherium albigense*, which our analyses have confirmed.
2) whether *Molassitherium* and *Epiaceratherium* were monophyletic in their previous acceptation. However here the results cannot support their monophyly, but a larger taxonomic sampling is necessary to support this claim.
3) whether the specimen NMB.O.B.928 from Rheinbetts could be confidently referred to *Epiaceratherium magnum*, which has been confirmed by the analyses.
4) whether the specimens from Bumbach could be referred to *Molassitherium* (as suggested by Scherler et al., 2013: Online Resource 1), which has been partly refuted by the results.

Thus, more taxa were needed to test at least two hypotheses: monophyly of *Molassitherium* and *Epiaceratherium* and refined taxonomic assignment of the specimens from Bumbach. To test them, we added six early-branching genera of Rhinocerotidae (*Uintaceras, Teletaceras, Penetrigonias, Trigonias, Amphicaenopus*, and *Subhyracodon*) in order to stabilize the tree base, through the analyses 6 to 11. Based on the results of these analyses, it became clear that *Molassitherium* as originally refined was not monophyletic. However, no referral to known species can be proposed for the specimens from Bumbach based on these analyses.

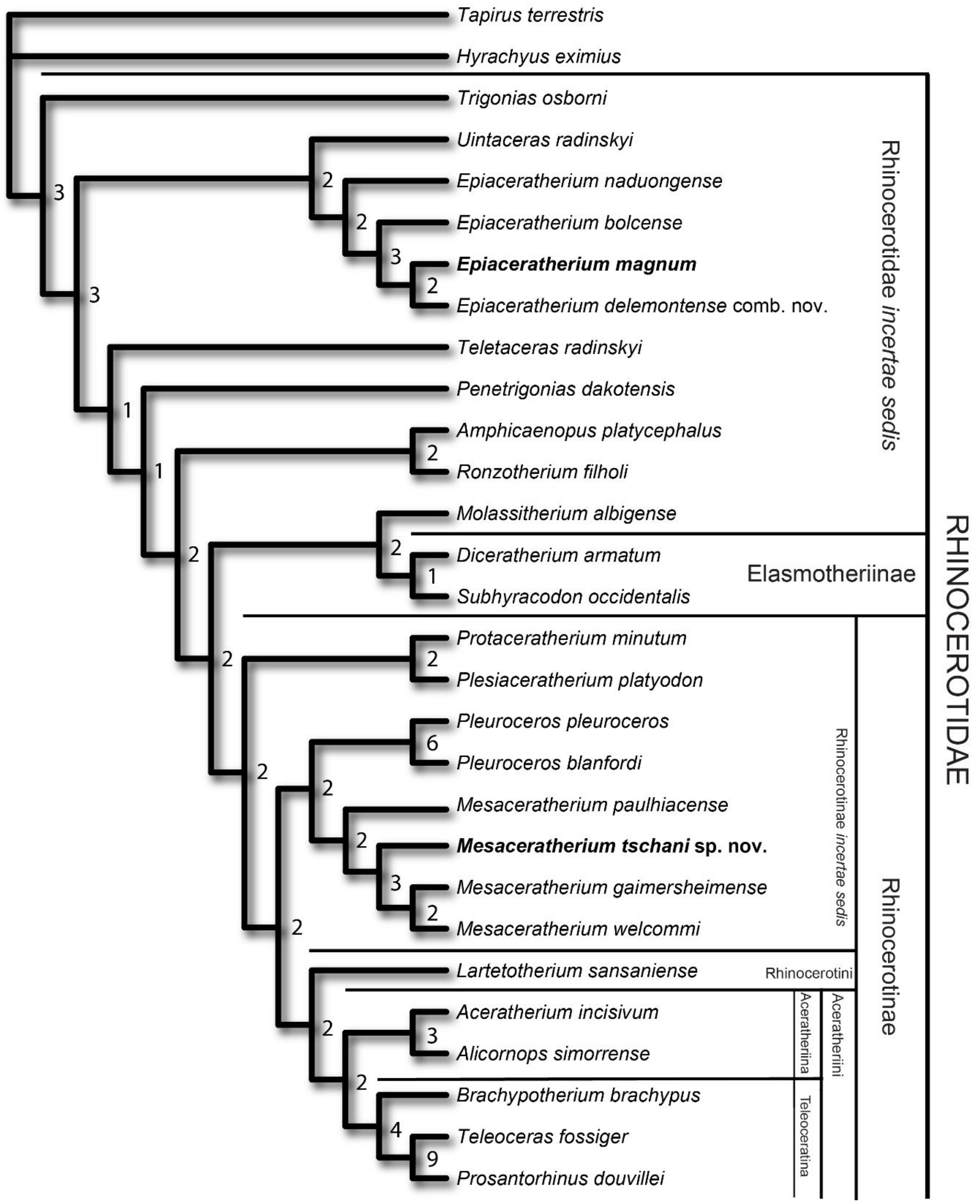

Figure 4: Single most parsimonious tree of the Rhinocerotidae using the heuristic search in PAUP* Version 4.0a (build 167) with a matrix of 288 morphological characters and 28 terminals, with *Tapirus terrestris* and *Hyrachyus eximius* as outgroups. Tree length = 1099 steps, RI = 0.43, CI = 0.3. Bremer support values are reported at the nodes. Newly described specimens from Rheinbetts and Bumbach are attributed to *Epiaceratherium magnum* and *Mesaceratherium tschani* sp. nov., respectively.

We then added several more derived rhinocerotid taxa to further test the non-monophyly of *Molassitherium* and get a stable placement for the specimens from Bumbach, which tended to branch close to *Pleuroceros* and/or *Mesaceratherium* without clear preference. Thus, during analyses 12 to 20, nine new genera were added, comprising Oligocene to Miocene genera of Rhinocerotidae, representing two subfamilies: Elasmotheriinae and Rhinocerotinae (comprising Rhinocerotini and Aceratheriini [Aceratheriina + Teleoceratina]) as well as *Ronzotherium*, an early large-sized rhinocerotid. Finally, the lesser known species *Mesaceratherium paulhiacense* was added to further test the monophyly of *Mesaceratherium*, and the placement of the specimens from Bumbach. Based on these results, these specimens are attributed to *Mesaceratherium tschani* sp. nov. This is the placement resulting from most of our analyses, including the most comprehensive ones, and for which the topology of tree is the most stable. Thus, adding more taxa would now be unnecessary, although it could lead to new topologies. Indeed, our identifications would probably not drastically change as they are well supported by numerous characters. The definitive tree (single most parsimonious tree) resulting from the most complete taxonomical sampling is presented in Figure 4.

Our new phylogeny leads to an important change concerning the monophyly of *Molassitherium*, which cannot be supported by our results. Indeed, the species "*M. delemontense* Becker & Antoine, 2013" should be assigned to *Epiaceratherium* instead. "*M. delemontense*" was erected on a very well preserved skull from Poillat (Jura Canton, Switzerland), which shared numerous similarities with the skull of *Molassitherium albigense*. However, based on our results, it shares more characters with *Epiaceratherium* than with *Molassitherium*, such as: a *foramen infraorbitalis* above P3 (**char. 2**), as in *E. bolcense*, but also *M. albigense*; a sharply deviating anterior tip of the zygomatic process in ventral view (**char. 37**), as in *E. bolcense*, but contrary to *M. albigense*; a long premolars series compared to the molars (**char. 63**), a synapomorphy of all epiaceratheres, also found in *M. albigense*; the absence of labial cingulum on upper premolars (**char. 83**) and on upper molars (**char. 109**), contrary to *M. albigense*; the hypocone connected to the ectoloph of P2-4 (**char. 86**), as in *E. magnum*, but also *M. albigense*; the separate protocone and hypocone on P2 (**char. 94**), as in *E. magnum*, but also *M. albigense*; the transverse metaloph on P2 (**char. 95**), a synapomorphy of all epiaceratheres and *Uintaceras*, not found in *M.*

albigense; a lingual wall on P3-4 (**char. 102**), a synapomorphy of *Epiaceratherium* and *Uintaceras*, that may also be found in *M. albigense*; an hypocone anterior to the metacone on P3-4 (**char. 103**), a character found in all early-branching Rhinocerotidae, but not in *M. albigense*; a continuous metaloph on P4 (**char. 108**), as in *E. magnum*, but also *M. albigense*; an antecrochet always present on upper molars (**char. 110**), a synapomorphy of all *Epiaceratherium* species, that is not always found in *M. albigense*; the lingual cingulum usually absent on upper molars (**char. 114**), a synapomorphy of *Epiaceratherium*, whereas it is usually present in *M. albigense*; a constriction of the protocone always present on M1-2 (**char. 115**), as in *E. bolcense* and *E. magnum*, which is more often absent on *M. albigense*; the absence of metacone fold on M1-2 (**char. 119**), as in *E. magnum*, but also *M. albigense*; a long metastyle on M1-2 (**char. 120**), as in *E. bolcense* and *E. magnum*, but also *M. albigense*; a posterior part of the ectoloph of M1-2 concave (**char. 122**), as in *E. magnum*, but also *M. albigense*; the presence of a lingual groove on the hypocone of M2 (**char. 129**), as in *E. naduongense* and *E. magnum*, but also sometimes on *M. albigense*; the presence of a mesostyle on M2 (**char. 130**), as in *E. bolcense* and *E. magnum*, but also *M. albigense*; a fused ectoloph on M3 (**char. 133**), as in *E. magnum*, as well as *M. albigense*; a protocone always constricted on M3 (**char. 135**) as in *E. bolcense* and *E. magnum*, but contrary to *M. albigense*; and finally, a strong metacone fold on P2 (**char. 285**) and on P3-4 (**char. 286**), as most early-branching Rhinocerotidae, but contrary to *M. albigense*. Thus, although "*M. delemontense*" shares 12 homoplastic synapomorphies with *Epiaceratherium* that are also found in *M. albigense*, it also shares with *Epiaceratherium* 11 synapomorphies that are not found in *Molassitherium*. Therefore, in agreement with these results we propose the new combination "*Epiaceratherium delemontense* (Becker & Antoine, 2013)". This could imply that this species would have had, like all epiaceratheres, a complete upper dentition, including three pairs of incisors and one pair of canines. A hypothetical representation of the complete skull of *Epiaceratherium* is shown in Figure 5 and was generated by virtually assembling the type cranium of *E. delemontense* comb. nov. (specimen MJSN POI007–245; 3D model available in [37]) to the snout of *E. bolcense* (NMB.I.O.43) and the mandible from Rheinbetts (NMB.O.B.928) attributed to *E. magnum* (see Tissier, Antoine and Becker, In Prep [26] for details on the protocol). Thus, this representation does not have any anatomical value, and is strictly theoretical, in the aim of visualising the speculative cranial morphology of *Epiaceratherium*.

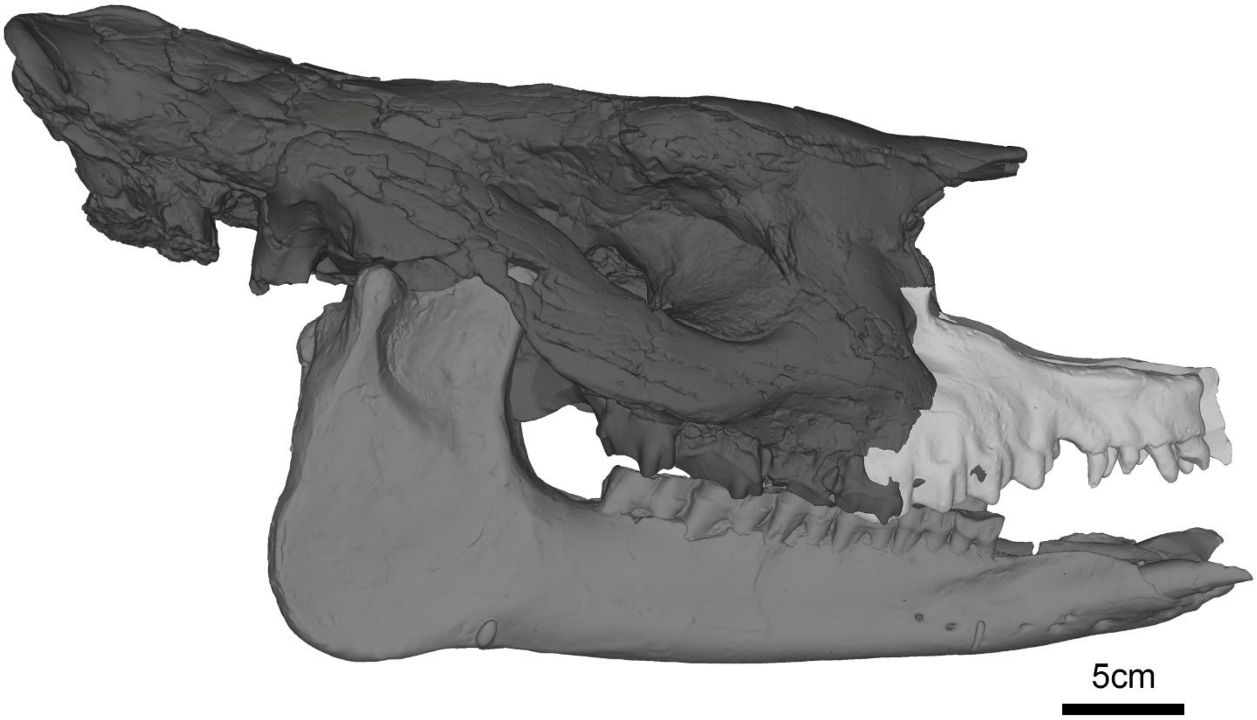

Figure 5: Archetypic reconstruction of the skull of *Epiaceratherium*, generated by 3D virtual association of the cranium of *E. delemontense* comb. nov. (in dark gray), mandible of *E. magnum* (in medium gray) and snout of *E. bolcense* (in light gray). 3D model available in Tissier, Antoine & Becker (In Prep.) [26]

2. Evolution of the anterior dentition in early Rhinocerotidae

The reduction of the anterior dentition is one of the major adaptative traits of the Rhinocerotidae, which developed much earlier than the horn(s). Indeed, the first horns may have appeared in the American genus *Diceratherium*, from the Early Oligocene [27,38,39] whereas the reduction of the anterior dentition was initiated already in Late Eocene times, notably with *Trigonias*, a well-known genus from North America.

The lower anterior dentition of *Trigonias* has been subject to some confusion. Lucas [40] originally considered the alveolus just behind the tusk-shaped i2 as the alveolus for i3 when he created this genus, but later, Gregory and Cook [41] believed that it could be in fact the alveolus for a canine. Indeed, on one mandible from a juvenile individual ("No. 1027"), two alveoli are present distally to i2, which indicates the presence of both i3 and a lower canine (or di3 and dc). Based on the shape and position of these alveoli, they suggested that the single tooth retained in the adult was the canine, instead of i3. However, Russell [42] showed that in some specimens the canine was definitely lost in the subadult while the i3 was still present. Thus, the lower canine is lost in the adult, but may still be present in the juvenile. However, Prothero, Manning & Hanson [38] and Antoine [27]

consider that the lower canine and i3 are lost in the adult *Trigonias* (see [38]: fig. 4, character 28 and [27]: p. 134), following Radinsky [43], who also considered the lower canine and i3 as lost in the adult, and not only in the juvenile. The confusion can perhaps be traced back up to Scott [44] who indicated the presence of three lower incisors in the dental formula of *Trigonias* (p. 776) yet later indicated in the description that "*of the third incisor and the canine no vestige remains*" (p. 777), perhaps meaning that these teeth were physically absent, but not their alveoli. On the contrary, Wood [45,46] considered the presence of the lower i3 as a defining generic character, whereas the presence of the lower canine would be variable. Here, we choose to follow Lucas [40] and Wood [45,46] and consider the lower i3 as present in the adult. Indeed, although the paratype mandible USNM-4815 (in Smithsonian National Museum of Natural History) belongs to an old individual with well-worn teeth, as reported by Wood [45], the alveoli for the i3 are still very large and not sealed (see Fig. 6), which indicates that this tooth would have been present during most of the animal's life. However, there may still be some intraspecific variability concerning the presence of this i3, since some individuals reported by Russell [42] show no trace of i3 (see fig. 26 for example).

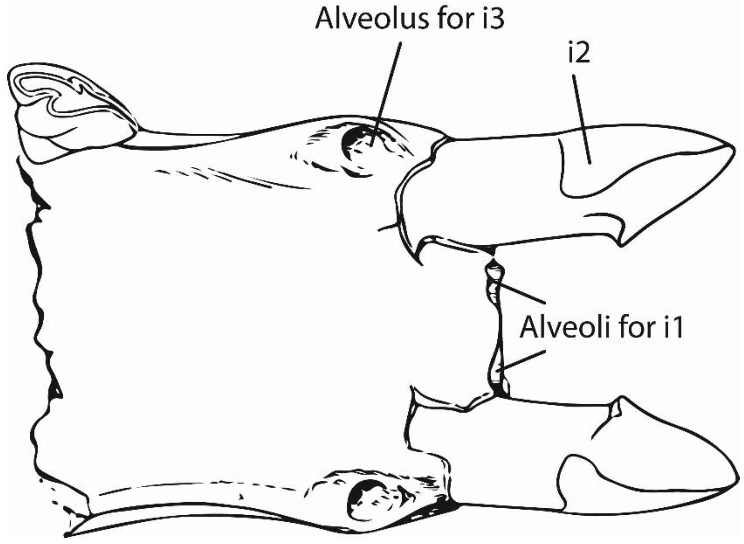

Figure 6: Drawing of the symphysis of the mandible USNM-4815, paratype of *Trigonias osborni*, modified from Lucas [40]

The evolution of the major traits concerning upper and lower anterior dentition is optimised on the most parsimonious tree, using the accelerated transformation parameter (ACCTRAN), favouring reversals instead of convergences (Fig. 7). Thus, according to our phylogeny, the absence of a lower canine can be regarded as a synapomorphy of the Rhinocerotidae, acquired quite early during the evolution of this group (Fig. 7). Yet, the presence/absence of an upper and/or lower canine remains a rather labile character in this group and may further

be subject to sexual dimorphism, as in other laurasiatheres, including perissodactyls. For example, the canine can be present in the adult male domestic horse, but not in the female [47]. Even within rhinoceroses, a rudimentary canine can sometimes be present in some individuals, as observed on one skull of *Dicerorhinus sumatrensis* (copy in MJSN collection). On some skulls of *Subhyracodon*, an upper canine may be present, although only on one side and not the other [38]. We nonetheless coded it as absent for this species, as it is the most-often seen condition. In other groups of perissodactyls, there can be an important sexual dimorphism on the size of the canine (e.g. in Lophiodontidae, as reported by Vautrin et al. [48]). Thus, this might explain why the lower canine could have reappeared at least twice during the evolution of the Rhinocerotidae, in *Uintaceras* and *Teletaceras*, for example (Fig. 7).

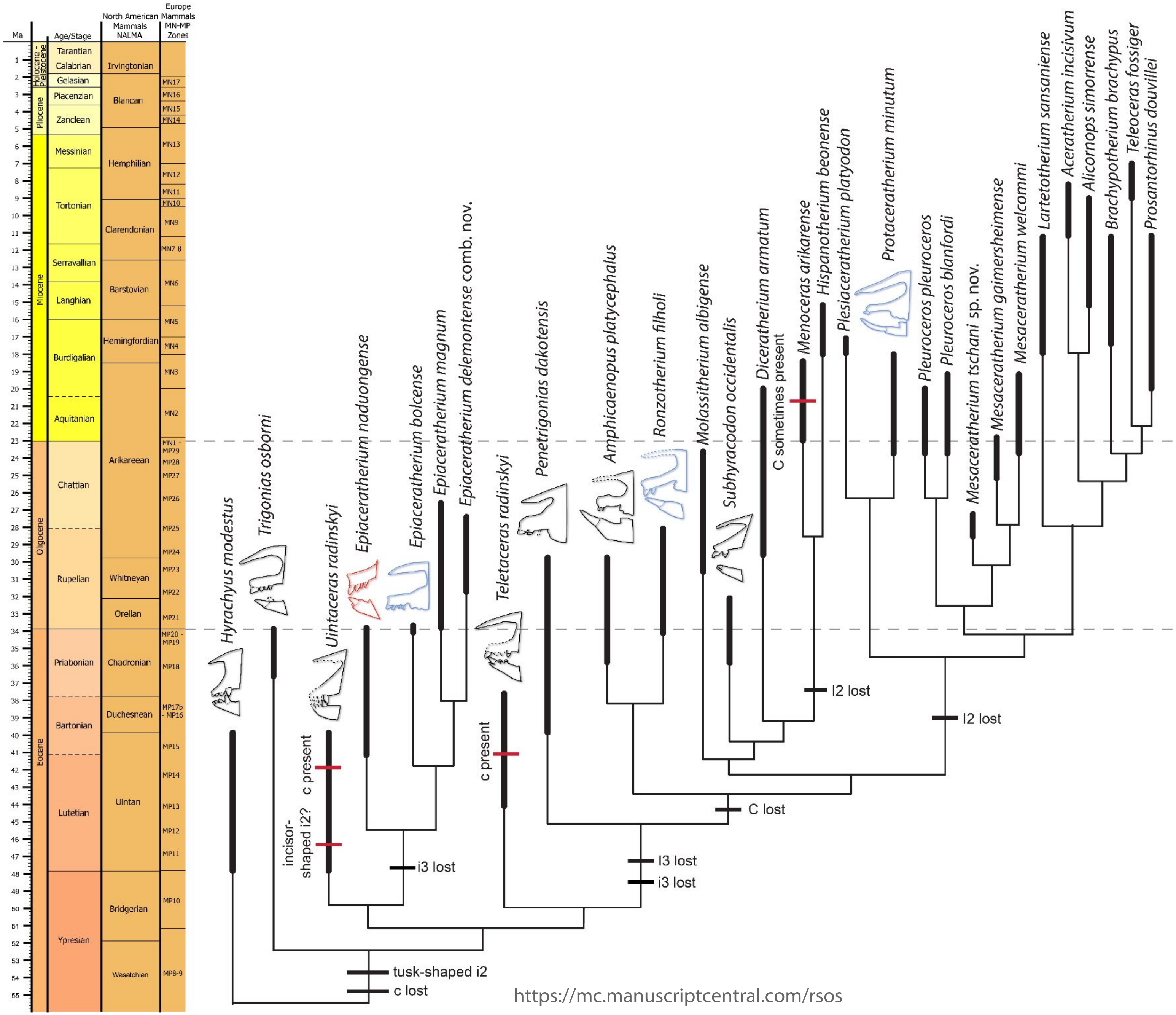
Ma
Age/Stage
North American Mammals NALMA
Europe Mammals MN-MP Zones
Holocene - Pleistocene
Pliocene
Miocene
Oligocene
Eocene
Tarantian
Calabrian
Gelasian
Piacenzian
Zanclean
Messinian
Tortonian
Serravallian
Langhian
Burdigalian
Aquitanian
Chattian
Rupelian
Priabonian
Bartonian
Lutetian
Ypresian
Irvingtonian
Blancan
Hemphilian
Clarendonian
Barstovian
Hemingfordian
Arikareean
Whitneyan
Orellan
Chadronian
Duchesnean
Uintan
Bridgerian
Wasatchian
Hyrachyus modestus
Trigonias osborni
Uintaceras radinskyi
Epiaceratherium naduongense
Epiaceratherium bolcense
Epiaceratherium magnum
Epiaceratherium delemontense comb. nov.
Teletaceras radinskyi
Penetrigonias dakotensis
Amphicaenopus platycephalus
Ronzotherium filholi
Molassitherium albigense
Subhyracodon occidentalis
Diceratherium armatum
Menoceras arikarense
Hispanotherium beonense
Plesiaceratherium platyodon
Protaceratherium minutum
Pleuroceros pleuroceros
Pleuroceros blanfordi
Mesaceratherium tschani sp. nov.
Mesaceratherium gaimersheimense
Mesaceratherium welcommi
Lartetotherium sansaniense
Aceratherium incisivum
Alicornops simorrense
Brachypotherium brachypus
Teleoceras fossiger
Prosantorhinus douvillei
C sometimes present
incisor-shaped i2?
c present
c present
i3 lost
I3 lost
i3 lost
C lost
I2 lost
I2 lost
tusk-shaped i2
c lost

Figure 7: Optimisation of characters 73-75, 79 and 81-82 with the ACCTRAN parameter in early-branching Rhinocerotidae, mapped on the single most parsimonious tree. Schematic depictions of anterior dentition are represented next to the taxon names where black lines indicate North American taxa, blue lines indicate European taxa and red lines indicate Asian taxa. Occurrences indicated by bold lines and are based on [1,12,19,25,39,49,50]. *Menoceras arikarense* and *Hispanotherium matritense* have been added to illustrate convergent reduction of anterior dentition among early Elasmotheriinae, and were placed according to previous formal phylogenies [19,25].

Another typical feature of the Rhinocerotidae is the tusk-shaped second lower incisor, which might have been acquired at the same time as the lower canine was lost (Fig. 7). It is indeed tusk-shaped in all Rhinocerotidae (when it is present), except in *Uintaceras* who might still have retained an incisor-shaped i2 [51]. However, the anterior dentition of this genus remains poorly known, and was not found in connection with the mandible, but the roots preserved in the lower jaw suggest nonetheless that i2 may have been smaller than i3.

Epiaceratherium is peculiar, as it possesses a complete upper anterior dentition, but has lost its i3 and c. This differs from the condition seen in all other rhinocerotids, in which I3 is lost at the same time as i3. Thus, i3 has been lost at least twice independently among Rhinocerotidae. I3 is also the first upper anterior tooth lost, before the successive loss of C and I2, and contrary to what occurs in the lower jaw. In *Penetrigonias dakotensis*, I3 is lost while C is unambiguously retained on the anterior tip of the maxilla [39]. However, Russell [42] identified I3 on the premaxilla in *Penetrigonias sagittatus*. Yet, Prothero [39] considers it as a canine. Thus, more investigation would be needed to determine the exact condition in this species. Finally, the loss of I2 is a distinguishing feature of Rhinocerotinae and Elasmotheriini, who retain only the typical chisel-tusk complex formed by I1/i2, as well as a non-functional i1 in many taxa.

Conclusion

Based on parsimony analyses of Rhinocerotidae, we identify an unpublished mandible (NMB.O.B.928) from the "middle" Oligocene of Rheinbetts (Basel Canton, Switzerland) as belonging to *Epiaceratherium magnum*. As the first mandible available for this species, it allows for assessing the absence of i3 and c. Along with this mandible, newly prepared specimens from Bumbach (MP25, early Late Oligocene, Switzerland) are referred to a new species: *Mesaceratherium tschani* sp. nov. These remains document the oldest occurrence of *Mesaceratherium*, which was previously only known from MP28 onward.

Our new phylogenetic analyses support a new combination: *Epiaceratherium delemontense* (Becker & Antoine, 2013) comb. nov. replaces "*Molassitherium delemontense* Becker & Antoine, 2013". Optimisation of concerned characters further allows for inferring that this species would have retained a complete upper anterior dentition, as in all other species assigned to *Epiaceratherium*. A new evolutionary scenario is proposed for tracking anterior dentition reduction in Rhinocerotidae. Based on this new scenario, the third lower incisor would have been lost at least twice independently, whereas I3 and C would have been lost only once, successively.

References

1. Antoine P-O, Ducrocq S, Marivaux L, Chaimanee Y, Crochet J-Y, Jaeger J-J, Welcomme J-L. 2003 Early rhinocerotids (Mammalia: Perissodactyla) from South Asia and a review of the Holarctic Paleogene rhinocerotid record. *Can. J. Earth Sci.* **40**, 365–374. (doi:10.1139/e02-101)

2. Wang H, Bai B, Meng J, Wang Y. 2016 Earliest known unequivocal rhinocerotoid sheds new light on the origin of Giant Rhinos and phylogeny of early rhinocerotoids. *Sci. Rep.* **6**. (doi:10.1038/srep39607)

3. Stehlin HG. 1909 Remarques sur les faunules de Mammifères des couches éocènes et oligocènes du Bassin de Paris. *Bull. la Société géologique Fr.* **4**, 488–520.

4. Brunet M. 1979 *Les grands mammifères chefs de file de l'immigration Oligocène et le problème de la limite Eocène-Oligocène en Europe*. Paris: Fondation Singer-Polignac.

5. Becker D. 2009 Earliest record of rhinocerotoids (Mammalia: Perissodactyla) from Switzerland: systematics and biostratigraphy. *Swiss J. Geosci.* **102**, 489–504. (doi:10.1007/s00015-009-1330-4)

6. Hooker JJ. 2010 The 'Grande Coupure' in the Hampshire Basin, UK: taxonomy and stratigraphy of the mammals on either side of this major Palaeogene faunal turnover. In *Micropalaeontology, Sedimentary Environments and Stratigraphy: A Tribute to Dennis Curry (1912–2001)*, pp. 147–215. (doi:10.1144/tms004.8)

7. Cerdeño E. 1998 Diversity and evolutionary trends of the Family Rhinocerotidae (Perissodactyla). *Palaeogeogr. Palaeoclimatol. Palaeoecol.* **141**, 13–34. (doi:10.1016/S0031-0182(98)00003-0)

8. Legendre S. 1989 Les communautés de mammifères du Paléogène (Eocène supérieur et Oligocène) d'Europe occidentale : structures, milieux et évolution. *Münchner Geowissenschaftliche Abhandlungen A* **16**, 1–110. (doi:10.1080/02724634.1991.10011409)

9. Jehenne Y, Brunet M. 1992 Intérêt biochronologique de quelques grands mammifères ongulés de l'éocène supérieur et de l'Oligocène d'Europe. *Geobios* **25**, 201–206. (doi:10.1016/S0016-6995(06)80329-3)

10. Uhlig U. 1999 Paleobiogeography of some Paleogene Rhinocerotoids (Mammalia) in Europe. *Acta Palaeontol. Rom.* **2**, 477–481.

11. Dashzeveg D. 1991 Hyracodontids and Rhinocerotids (Mammalia, Perissodactyla, Rhinocerotoidea) from the Paleogene of Mongolia. *Palaeovertebrata* **21**, 1–84.

12. Böhme M *et al.* 2013 Na Duong (northern Vietnam) - An exceptional window into Eocene ecosystems from Southeast Asia. *Zitteliana R. A Mitteilungen der Bayer. Staatssammlung fur Palaontologie und Geol.* **53**, 120–167.

13. Dal Piaz GB. 1930 *I mammiferi dell'oligocene veneto:* Trigonias ombonii. Padova: Societa Cooperativa Tipografica.

14. Uhlig U. 1999 *Die Rhinocerotoidea (Mammalia) aus der unteroligozänen Spaltenfüllung Möhren 13 bei Treuchtlingen in Bayern*. München: Verlag der Bayerischen Akademie der Wissenschaften Abhandlungen. Neue Folge, No. 170.

15. Pandolfi L *et al.* 2017 Reassessing the earliest Oligocene vertebrate assemblage of Monteviale (Vicenza, Italy). *J. Syst. Palaeontol.* **15**, 83–127. (doi:10.1080/14772019.2016.1147170)

16. Heissig K. 1969 Die Rhinocerotidae (Mammalia) aus der oberoligozänen Spaltenfüllung von Gaimersheim bei Ingolstadt in Bayern und ihre phylogenetische Stellung. *Abhandlungen der Bayer. Akad. der Wissenschaften, Math. Klasse* **138**.

17. Ménouret B, Guérin C. 2009 *Diaceratherium massiliae* nov. sp. des argiles oligocènes de Saint-André et Saint-Henri à Marseille et de Les Milles près d'Aix-en-Provence (SE de la France), premier grand Rhinocerotidae brachypode européen. *Geobios* **42**, 293–327. (doi:10.1016/j.geobios.2008.10.009)

18. Abel O. 1910 Kritische Untersuchungen über die paläogenen Rhinocerotiden Europas. *Abhandlungen der Geol. Reichsanstall, Wien* **20**, 1–52.

19. Becker D, Antoine P-O, Maridet O. 2013 A new genus of Rhinocerotidae (Mammalia, Perissodactyla) from the Oligocene of Europe. *J. Syst. Palaeontol.* **11**, 947–972. (doi:10.1080/14772019.2012.699007)

20. Roman F. 1912 Les Rhinocéridés de l'Oligocène d'Europe. *Arch. du Muséum d'histoire Nat. Lyon* **11**, 1–92.

21. Antoine PO, Métais G, Orliac MJ, Peigné S, Rafaÿ S, Solé F, Vianey-Liaud M. 2011 A new late Early Oligocene vertebrate fauna from Moissac, South-West France. *Comptes Rendus - Palevol* **10**, 239–250. (doi:10.1016/j.crpv.2011.01.004)

22. Blanchon M, Antoine P-O, Blondel C, de Bonis L. 2018 Rhinocerotidae (Mammalia, Perissodactyla) from the latest Oligocene Thézels locality, SW France, with a special emphasis on *Mesaceratherium gaimersheimense* Heissig, 1969. *Ann. Paléontologie* **104**, 217–229. (doi:10.1016/j.annpal.2018.06.001)

23. Tissier J, Becker D. 2018 Nouvelle occurrence du genre *Mesaceratherium* (Rhinocerotidae) dans l'Oligocène supérieur du Bassin molassique suisse. *Actes la Société jurassienne d'émulation* **120**, 57–74. (doi:10.5169/seals-772321)

24. Richard M. 1937 Une nouvelle espèce de Rhinocéridé aquitanien : *Diaceratherium pauliacensis*. *Bull. la Société d'histoire Nat. Toulouse.* **71**, 165–170.

25. Antoine P-O, Downing KF, Crochet JY, Duranthon F, Flynn LJ, Marivaux L, Métais G, Rajpar A rahim, Roohi G. 2010 A revision of *Aceratherium blanfordi* Lydekker, 1884 (Mammalia: Rhinocerotidae) from the Early Miocene of Pakistan: Postcranials as a key. *Zool. J. Linn. Soc.* **160**, 139–194. (doi:10.1111/j.1096-3642.2009.00597.x)

26. Tissier J, Antoine P-O, Becker D. In press. 3D models related to the publication: New material of *Epiaceratherium* and a new species of *Mesaceratherium* clear up the phylogeny of the early Rhinocerotidae (Perissodactyla). *MorphoMuseuM*

27. Antoine P-O. 2002 Phylogénie et évolution des Elasmotheriina (Mammalia, Rhinocerotidae). *Mémoires du Muséum Natl. d'Histoire Nat.* **188**, 1–359.

28. Swofford DL. 2002 PAUP*. Phylogenetic Analysis Using Parsimony (*and Other Methods). Version 4.

29. Owen R. 1848 *On the archetype and homologies of the vertebrate skeleton*. London: John Van Voorst. (doi:10.5962/bhl.title.118611)

30. Owen R. 1845 *Odontography*. London.

31. Gray JE. 1821 On the natural arrangement of vertebrose animals. *London Med. Repos.* **15**, 296–310.

32. Antoine P-O, Becker D. 2013 A brief review of Agenian rhinocerotids in Western Europe. *Swiss J. Geosci.* **106**, 135–146. (doi:10.1007/s00015-013-0126-8)

33. Yan D, Heissig K. 1986 Revision and Autopodial Morphology of the Chinese-European Rhinocerotid Genus *Plesiaceratherium* Young 1937. *Zitteliana* **14**, 81–109.

34. Roman F. 1924 Contribution à l'étude de la faune de Mammifères des Littorinenkalk (Oligocène supérieur) du bassin de Mayence. Les Rhinocéros. *Trav. Doc. des Lab. Géologie Lyon* **7**.

35. Antoine P-O, Duranthon F, Hervet S, Fleury G. 2006 Vertébrés de l'Oligocène terminal (MP30)

et du Miocène basal (MN1) du métro de Toulouse (Sud-Ouest de la France). *Comptes Rendus Palevol* (doi:10.1016/j.crpv.2006.03.007)

36. Lihoreau F, Ducrocq S, Antoine P-O, Vianey-Liaud M, Rafaÿ S, Garcia G, Valentin X. 2009 First complete skulls of *Elomeryx crispus* (Gervais, 1849) and of *Protaceratherium albigense* (Roman, 1912) from a new Oligocene locality near Moissac (SW France). *J. Vertebr. Paleontol.* **29**, 242–253. (doi:10.1671/039.029.0114)

37. Maridet O, Rauber G, Rochat M, Roch R, Tissier J, Anquetin J, Becker D. 2019 3D surface scan of the type specimen of *Molassitherium delemontense* Becker and Antoine, 2013: use of a 3D model for research and conservation issues. *MorphoMuseuM* **5**, e82. (doi:10.18563/journal.m3.82)

38. Prothero DR, Manning E, Hanson CB. 1986 The phylogeny of the Rhinocerotoidea (Mammalia, Perissodactyla). *Zool. J. Linn. Soc.* **87**, 341–366. (doi:10.1111/j.1096-3642.1986.tb01340.x)

39. Prothero DR. 2005 *The evolution of North American rhinoceroses*. Cambridge, UK: Cambridge University Press.

40. Lucas FA. 1900 A new rhinoceros, *Trigonias osborni*, from the Miocene of South Dakota. *Proc. United States Natl. Museum* **23**, 221–224. (doi:10.5479/si.00963801.23-1207.221)

41. Gregory WK, Cook HJ. 1928 New Material for the Study of Evolution. *Proc. Denver Museum Nat. Hist.* **8**.

42. Russell LS. 1982 Tertiary Mammals of Saskatchewan Part VI: The Oligocene Rhinoceroses. *Life Sci. Contrib. R. Ontario Museum, Toronto* **133**, 1–58.

43. Radinsky LB. 1966 The Families of The Rhinocerotoidea (Mammalia, Perissodactyla). *J. Mammal.* **47**, 631–639. (doi:10.2307/1377893)

44. Scott WB. 1941 The Mammalian Fauna of the White River Oligocene: Part V. Perissodactyla. *Trans. Am. Philos. Soc. New Ser.* **28**, 747–964.

45. Wood HE. 1927 Some early Tertiary rhinoceroses and hyracodonts. *Bull. Am. Paleontol.* **13**, 165–249.

46. Wood HE. 1931 Lower Oligocene Rhinoceroses of the Genus *Trigonias*. *J. Mammal.* **12**, 414–428.

47. Olsen SL. 2006 Early horse domestication on the Eurasian steppe. *Doc. Domest. New Genet. Archaeol. Paradig.* , 245–269.

48. Vautrin Q, Tabuce R, Laurent Y, Vidalenc D, Lihoreau F. 2019 Intraspecific variation of *Eolophiodon laboriense*, a basal Lophiodontidae (Mammalia, Perissodactyla) from the early Eocene of Southern France. *Geobios* **53**, 51–63. (doi:10.1016/j.geobios.2019.02.005)

49. Radinsky LB. 1967 *Hyrachyus*, *Chasmotherium*, and the Early Evolution of Helaletid Tapiroids. *Am. Museum Novit.* **2313**, 1–23.

50. Becker D, Tissier J. 2019 Rhinocerotidae from the early middle Miocene locality Gračanica (Bugojno Basin, Bosnia-Herzegovina). *Palaeobiodiversity and Palaeoenvironments* (doi:10.1007/s12549-018-0352-1)

51. Holbrook LT, Lucas SG. 1997 A new genus of Rhinocerotoid from the Eocene of Utah and the status of North American '*Forstercooperia*'. *J. Vertebr. Paleontol.* **17**, 384–396. (doi:10.1080/02724634.1997.10010983)

Supplementary Material 1: Morphological characters matrix in Nexus format, for PAUP.

Supplementary Material 2: Trees or consensus trees resulting from the parsimony analyses presented in Table 5.

Appendix B

Dear Editors,

We are pleased to resubmit our revised version of the manuscript RSOS-200633 entitled "New material of *Epiaceratherium* and a new species of *Mesaceratherium* clear up the phylogeny of the early Rhinocerotidae (Perissodactyla)". Thank you for having processed our submission so efficiently. Constructive remarks of the reviewers have been very appreciated. Please find below our response to their comments.

In addition to these changes, we also updated all collection numbers from the Natural History Museum of Bern to their definitive numbers and corrected measurements for the radius of *Mesaceratherium tschani* in Table 3. All changes can be visualised in the track-changes manuscript.

Associate Editor Comments to Author (Professor Allison Daley):

Both reviewers gave very positive assessments of the manuscript, commenting on the high quality of the descriptions and phylogenetic analysis. Please incorporate the reviewers requests to add additional images to Figures 2 and 3, and modifications to the data Tables 3 and 4.

As requested by reviewers, we added the dorsal view of the symphysis in Fig. 2 (Fig. 2B) as well as anterior views of the humerus (Fig. 3A and C) and proximal views of the metatarsals (Fig. 3R and S).

We also added the measurements for *M. welcommi* and *M. paulhiacense* in tables 3 and 4.

Please also add text to the manuscript addressing the comment about specimen NMB I.O.43, that is a hypothetical reconstruction of the snout (in gypsum) and it is not an original specimen. In addition, make the minor changes suggested in the annotated PDF files.

We added the text to specify that the snout specimen is reconstructed in gypsum and based on several specimens.

Reviewer 1:

This paper shows very good result for main part of description and comparisons.
I have found a few issues that, once addressed, will improve the manuscript.

- This paper shows various abbreviations for measurements of postcranials in tables 3, 4 and p. 14, i. 34. therefore, please add to the explanations of those abbreviations in full in Terminologyy section.

Agreed, we added these abbreviations in the terminology section.

- This paper discusses the reduction of the anterior dention among rhinocerotids. If it possible, I suggest to add a picture of dorsal view of the symphysis part of the Mesaceratherium tschani (NMBE-D1836/1837) in Fig. 2 because it would be difficult for some scholars to understand the detailed morphology of the part.

Agreed, we added the dorsal view of the symphysis in Fig. 2 (Fig. 2B).

- In this paper, various articular facets of the postcranials of Mesaceratherium tschani are triated

as key characters in the diagnosis and comparisons sections. Therefore, I suggest to add nomenclatures of those facets in Fig. 3.

Agreed, we added captions of the articular facets in Fig. 3.

We also made the minor changes suggested in the annotated PDF:

- We added a photo of the external rugosities in Fig 1.
- We added a reference for the source of the characters of Eggysodontidae, Hyracodontidae, or Paraceratheriidae, as well as *Ronzotherium* and *Diaceratherium*

Reviewer 2:

The manuscript deals with the description of new rhinocerotids remains from the Oligocene of Europe and their significative contribution in the understanding of the evolution of early Rhinocerotidae. The manuscript is well-written (but I' m not a native speaker); the descriptions are accompained by an exhaustive phylogenetic analysis and the data are available for the readers that will be enable to reproduce the analyses. The data reported in the manuscript will be very useful for future papers on early rhinocerotids. The conclusions are well-supported by data. I added only a few comments in the attached files. The main comment is regarding the specimen NMBIO43; I added a comment with infos that the authors could be added in the text if they want.

Thank you for this important information. We modified the text in consequence.

Minor comments are for tables and a figure and concerning lower teeth of Molassitherium delemontense. Anyway, these comments do not invalidate results, conclusions or analyses and I strongly recommend the publication of this manuscript.

We updated the tables and figures following these recommendations, and we also now compare the specimens to *Molassitherium delemontense* as suggested. (*"The lower teeth of Molassitherium delemontense are very poorly known [19, 35] and very similar, but they differ in having a slightly more oblique hypolophid on m1. Molassitherium cf. delemontense from Nuceto [35] have a more constricted paralophid on p2."*).

We also made the minor changes suggested in the annotated PDF:

- We added the text "belonging to three different families (Hyracodontidae, Amynodontidae and Rhinocerotidae)," as suggested
- We added mention of the lower teeth of *Molassitherium delemontense*
- Unfortunately, we could not add measurements of the lower cheek teeth of *Mesaceratherium paulhiacense* in Table 2, as they are unknown. The lower teeth of this species from "Métro de Toulouse" have now been reattributed to another species, *M. gaimersheimense* by Blanchon et al. 2018 (https://doi.org/10.1016/j.annpal.2018.06.001).
- We added photos of the anterior view of the two humerus and of the proximal view of the metatarsals.
- We added measurements of the postcranial measurements of *M. welcommi* and *M. paulhiacense*, for compaisons.
- We added the text to specify that the specimen NMB I.O.43 is a hypothetical reconstruction of the snout (in gypsum) and based on several specimens.
